# JMJD2C mediates the MDM2/p53/IL5RA axis to promote CDDP resistance in uveal melanoma

**DOI:** 10.1038/s41420-022-00949-y

**Published:** 2022-04-25

**Authors:** Qi Zhu, Han Chen, Xiaoying Li, Xi Wang, Hongtao Yan

**Affiliations:** Department of Ophthalmology, The Second Hospital of Jilin University, Changchun, 130000 People’s Republic of China

**Keywords:** Cell biology, Genetics

## Abstract

Chemotherapy resistance poses an obstacle for effective treatment of uveal melanoma. In this study, we aim to investigate the effects of jumonji domain containing 2C (JMJD2C)-mediated mouse double minute-2 homolog (MDM2)/p53/interleukin 5 receptor subunit alpha (IL5RA) axis on cisplatin (CDDP) resistance in uveal melanoma. RT-qPCR and Western blot assay were performed to determine their expression patterns in uveal melanoma cell line (MUM-2B) and CDDP-resistant cell line (MUM-2B/CDDP). The enrichment of H3K9me3 in MDM2 promoter region was examined by ChIP, and the binding between p53 and ubiquitin in MUM-2B cells testified by co-IP assay. Following overexpression or silencing of JMJD2C/MDM2/p53/IL5RA, the 50% concentration of inhibition (IC50) and the biological characteristics of MUM-2B and MUM-2B/CDDP cells were examined using CCK-8 assay, SA-β-gal staining, fluorescence-activated cell sorting analysis, and Transwell assay. Finally, the tumorigenicity of transplanted MUM-2B and MUM-2B/CDDP cells in nude mice was assessed. JMJD2C was documented to be highly expressed in uveal melanoma cells, promoting the CDDP resistance. Histone demethylase JMJD2C removed the H3K9me3 modification of MDM2 promoter, which promoted the expression of MDM2. MDM2 enhanced the IL5RA expression through stimulating the ubiquitination and degradation of p53, thus inducing CDDP resistance of uveal melanoma cells. Furthermore, the results of in vivo experiments revealed that JMJD2C mediated the MDM2/p53/IL5RA axis to expedite the growth of uveal melanoma and augment the CDDP resistance. Taken together, JMJD2C can induce histone demethylation to upregulate MDM2, thereby ubiquitinating p53 and upregulating IL5RA. As a consequence, CDDP resistance in uveal melanoma is ultimately accelerated.

## Introduction

Uveal melanoma is considered to be the most common ocular cancer among adults that accounts for approximately 5% of the total [1]. Clinical examination using the slit lamp and indirect ophthalmoscope is employed for the diagnosis of uveal melanoma, in combination with ultrasonography of patients’ eyes [2]. Unfortunately, about 50% of patients with uveal melanoma may suffer from metastatic disease after effective primary therapy [3], with liver being the most frequently occurring site of metastases [4]. Cisplatin (CDDP) is one of the recommended chemotherapy drugs for the treatment of metastatic uveal melanoma [5]. However, the therapeutic efficacy for this malignancy is still limited by chemotherapy resistance [6]. In this context, it is vitally important to seek novel target for the control of chemotherapy resistance in uveal melanoma.

Jumonji domain-containing 2C (JMJD2C) is regarded as a histone lysine demethylase that participates in histone methylation, an important process of epigenetic modification [7]. Of note, a previous study has found that enforced expression of JMJD2C could contribute to enhanced Braf-V600E-driven melanomagenesis in mouse and zebrafish models [8]. Intriguingly, it has been reported that JMJD2C can promote mouse double minute-2 homolog (MDM2) expression *via* removal of H3K9me3 from the promoter region of MDM2 [9]. The co-inhibited MDM2 with Bcl-2/XL/W has been highlighted to function as a promising target for treatment of uveal melanoma [10]. Moreover, reduced upregulation of MDM2 dependent on microRNA-17-3p could aid in the prevention against the progression of uveal melanoma [11]. To our knowledge, MDM2 is a type of E3 ubiquitin ligase that exerts regulatory functions on the stability of p53 [12]. p53 has been identified as a transcription factor capable of modulating tumor suppressor activity which is often activated upon DNA damage and other cellular stress forms [13]. Downregulation of p53 by overexpressed microRNA-21 could accelerate the proliferative, migratory, and invading processes of uveal melanoma cell lines [14]. Based on the bioinformatics prediction of this study, interleukin 5 receptor subunit alpha (IL5RA) is a key differentially expressed gene in uveal melanoma and is the upstream regulatory gene of p53. Interestingly, it was unveiled that IL5RA was upregulated in Hodgkin’s lymphoma cell lines resistant to cytotoxic drugs including CDDP [15]. Considering all the aforementioned reports, we then hypothesize in the current study that JMJD2C may participate in the regulation of CDDP resistance in uveal melanoma, with the involvement of the MDM2/p53/IL5RA axis.

## Results

### JMJD2C may participate in the initiation and development of uveal melanoma by mediating the MDM2/p53/IL5RA axis

**Fig. 1:**
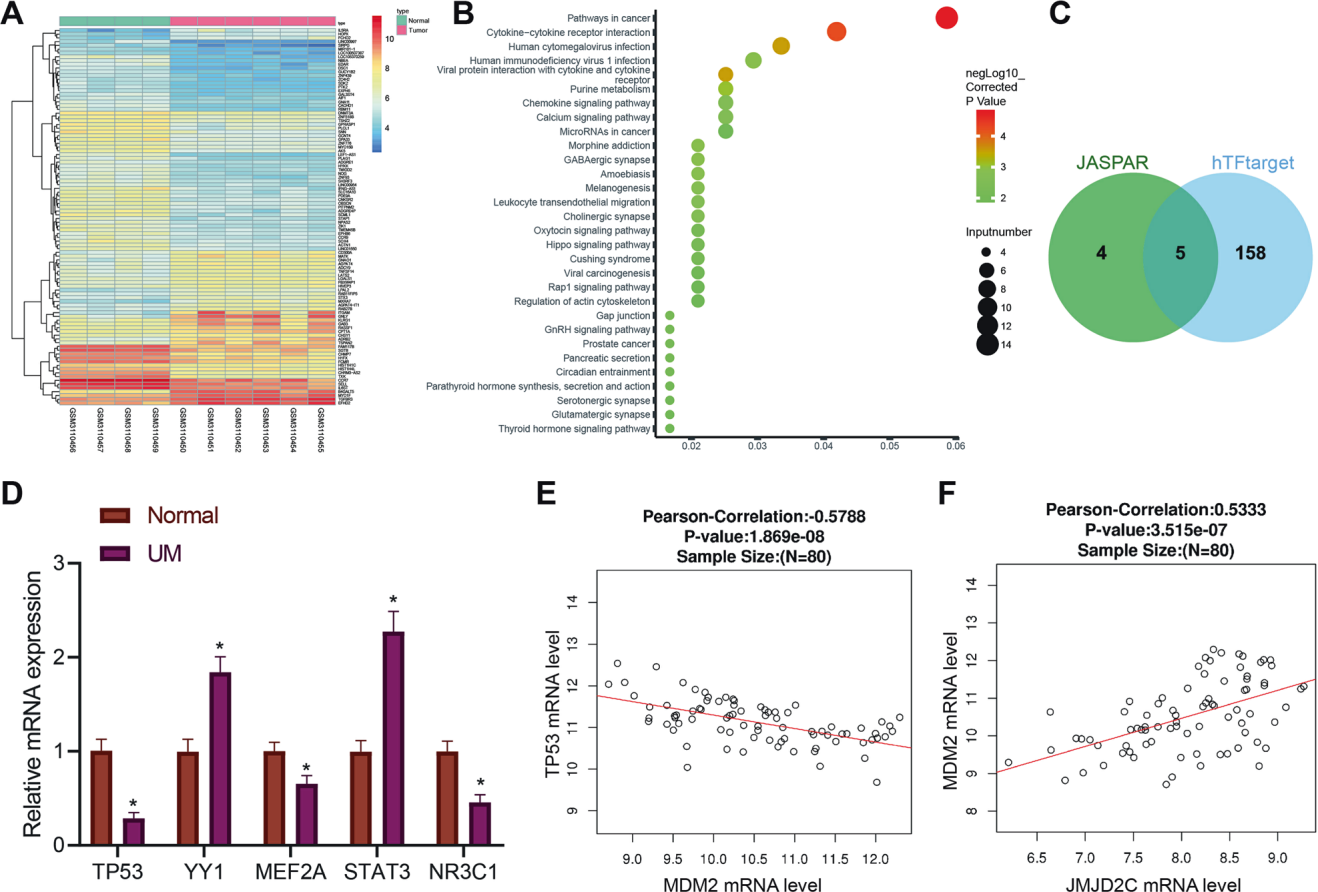
Possible molecular targets involved in the occurrence and development of uveal melanoma are screened by bioinformatics analysis. **A**The heatmap for differential gene expression in GSE113625 uveal melanoma dataset. *X*-axis represents sample number, *Y*-axis represents gene name, left dendrogram represents gene expression clustering, each small block in the figure represents the expression of a gene in a sample, and the upper right histogram is color scale. **B** KEGG pathway enrichment analysis of differentially expressed genes. *X*-axis represents GeneRatio, the *Y*-axis represents KEGG entry, the circle color represents the corrected enrichment *p* value, the size represents the number of genes enriched in the entry, and the right histogram is the color scale. **C** IL5RA upstream transcription factor prediction. Two circles in the figure represent the prediction results of JASPAR database and hTFtarget database, and the middle part represents the intersection of the two groups of data. **D** The expression of TP53, YY1, MEF2A, STAT3, and NR3C1 in uveal melanoma (*n* = 32) and normal controls (*n* = 30) determined by RT-qPCR. * *p* < 0.05. **E** Correlation analysis between TP53 and MDM2 mRNA expression in the uveal melanoma samples from TCGA database. The abscissa represents the expression value of MDM2, the ordinate represents the expression value of p53, and the *p* value the correlation and the Pearson’s correlation coefficient are presented in the top. **F** Correlation analysis between JMJD2C (KMD4C) and MDM2 mRNA expression in the uveal melanoma samples from TCGA database. *X*-axis represents JMJD2C expression value, *Y*-axis represents MDM2 expression value, and the *p* value for the correlation and Pearson’s correlation coefficient are presented in the top.

Through the GEO database, the GSE113625 microarray involving uveal melanoma was obtained. A total of 240 differentially expressed genes (Fig. 1A) were characterized by differential analysis of this microarray. Further KEGG pathway enrichment analysis exhibited a main enrichment of these differentially expressed genes in the “pathway in cancer” pathway (Fig. 1B). Then, the differentially expressed genes in the “pathway in cancer” pathway were selected, and their differential expression patterns in GSE113625 are shown in Table S1. Among them, IL5RA was considerably upregulated in uveal melanoma, exhibiting the largest fold change.

JASPAR and hTFtarget databases were subsequently adopted to predict the upstream transcription factors of IL5RA. The predicted results were intersected and five candidate transcription factors, TP53, YY1, MEF2A, STAT3 and NR3C1, were presented in the intersection (Fig. 1C). Further RT-qPCR determination of these five transcription factors revealed that TP53 was poorly expressed in uveal melanoma with the most significant difference (Fig. 1D). By searching the JASPAR database, we found the existence of TP53 binding domains in the promoter region of IL5RA (Table S2).

Published literature has demonstrated that MDM2 can degrade p53 through the ubiquitin proteasome pathway [12], and that p53 can inhibit the occurrence of uveal melanoma [14]. Further analysis was performed on the correlation between MDM2 and p53 mRNA expression in the uveal melanoma included in TCGA, which suggested a significant inverse correlation between MDM2 and p53 mRNA expression (Fig. 1E). An existing study has illuminated that JMJD2C can augment the expression of MDM2 by removing H3K9me3 in the promoter region of MDM2 [9]. Here, we analyzed their correlation in uveal melanoma included in TCGA, and demonstrated a significant positive correlation between JMJD2C and MDM2 mRNA expression in uveal melanoma (Fig. 1F). In light of the findings mentioned above, it is reasonable to suppose that JMJD2C may participate in the development of uveal melanoma.

### JMJD2C was overexpressed in uveal melanoma and promoted CDDP resistance of uveal melanoma

**Fig. 2:**
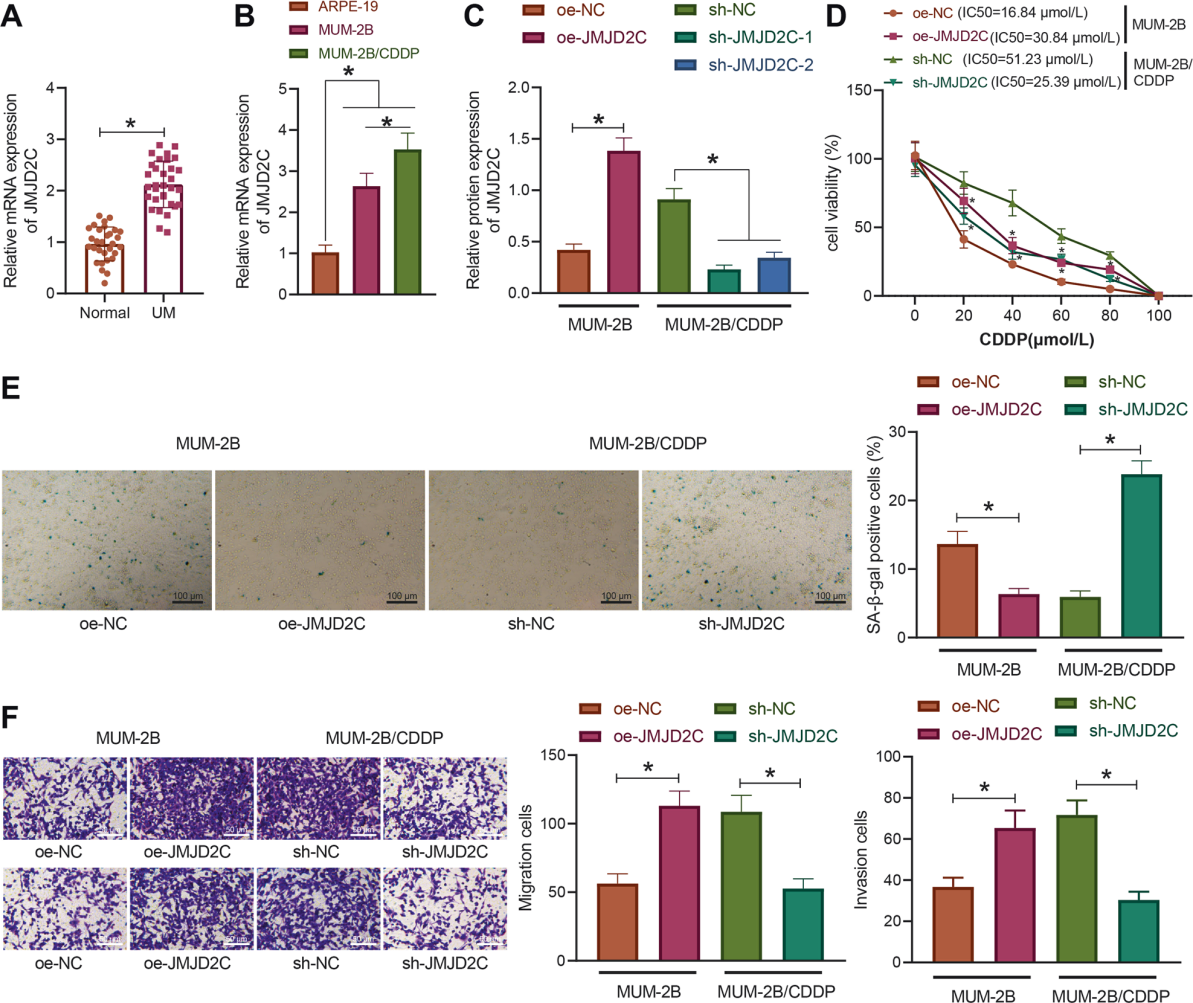
JMJD2C is overexpressed in uveal melanoma and promotes CDDP resistance. **A** The expression of JMJD2C in uveal melanoma tissues as determined by RT-qPCR (uveal melanoma = 32, normal = 30). **B** The expression of JMJD2C in normal uveal epithelial cells ARPE-19, uveal melanoma cells MUM-2B, and drug-resistant cell line MUM-2B/CDDP as determined by RT-qPCR. **C** The protein expression of JMJD2C after sh-JMJD2C or oe-JMJD2C treatment in the MUM-2B/CDDP cells and MUM-2B cells, respectively, as measured by Western blot assay. **D** The cell viability and IC50 in MUM-2B/CDDP cells after JMJD2C silencing and MUM-2B cells after JMJD2C overexpression as assessed by CCK-8 assay. **E** The senescence rate of MUM-2B/CDDP cells after JMJD2C silencing and MUM-2B cells after JMJD2C overexpression as assessed by SA-β-gal staining. **F** The migration and invasion abilities of MUM-2B/CDDP cells after JMJD2C silencing and MUM-2B cells after JMJD2C overexpression as assessed by Transwell assay. * *p* < 0.05. Cell experiments were repeated three times independently.

According to a recent report, JMJD2A can promote CDDP resistance in ovarian cancer cells [16]. Here, we aimed to examine the effect of JMJD2C on the drug resistance of uveal melanoma cells. RT-qPCR results displayed that the expression of JMJD2C in uveal melanoma tissues was significantly increased versus that in normal uveal tissues (Fig. 2A). In addition, the expression of JMJD2C was notably increased in the MUM-2B and MUM-2B/CDDP cells relative to the normal uveal epithelial cells ARPE-19, with MUM-2B/CDDP cells showing a more pronounced increase of JMJD2C expression (Fig. 2B). Collectively, JMJD2C is highly expressed in uveal melanoma as well as in CDDP-resistant cells.

Next, we overexpressed JMJD2C by overexpression plasmid in the MUM-2B cells and silenced JMJD2C with shRNAs in the MUM-2B/CDDP cells, respectively, and validated their efficiency utilizing Western blot assay (Figs. 2C and S4A). Meanwhile, sh-JMJD2C-1 showing the superior silencing efficiency (Fig. 2C) and was thus selected for subsequent experiments.

Furthermore, CCK-8 assay exhibited that in the context of CDDP treatment, the cell viability and IC50 were significantly increased in MUM-2B cells after overexpressing JMJD2C while decreasing in MUM-2B/CDDP cells upon silencing of JMJD2C (Fig. 2D). However, overexpression or silencing of JMJD2C without CDDP treatment had little effect on the viability of MUM-2B or MUM-2B/CDDP cells (Fig. S1). The results of SA-β-gal staining, fluorescence-activated cell sorting (FACS) analysis, and Transwell assay (Figs. 2E, F, and S2) found that elevation of JMJD2C contributed to reduced senescence rate of MUM-2B cells, more cells arrested in the S and G2/M phases, less cells arrested in the G1 phase, and notably boosted migration and invasion abilities. In contrast, JMJD2C knockdown in MUM-2B/CDDP cells markedly resulted in enhancement in cell senescence, promotion of cell cycle arrest, and suppression of migration and invasion abilities. It was unveiled that JMJD2C, highly expressed in uveal melanoma, reinforced CDDP resistance.

### JMJD2C upregulated MDM2 expression by removing histone methylation of MDM2 promoter and promoted CDDP resistance in uveal melanoma cells

**Fig. 3:**
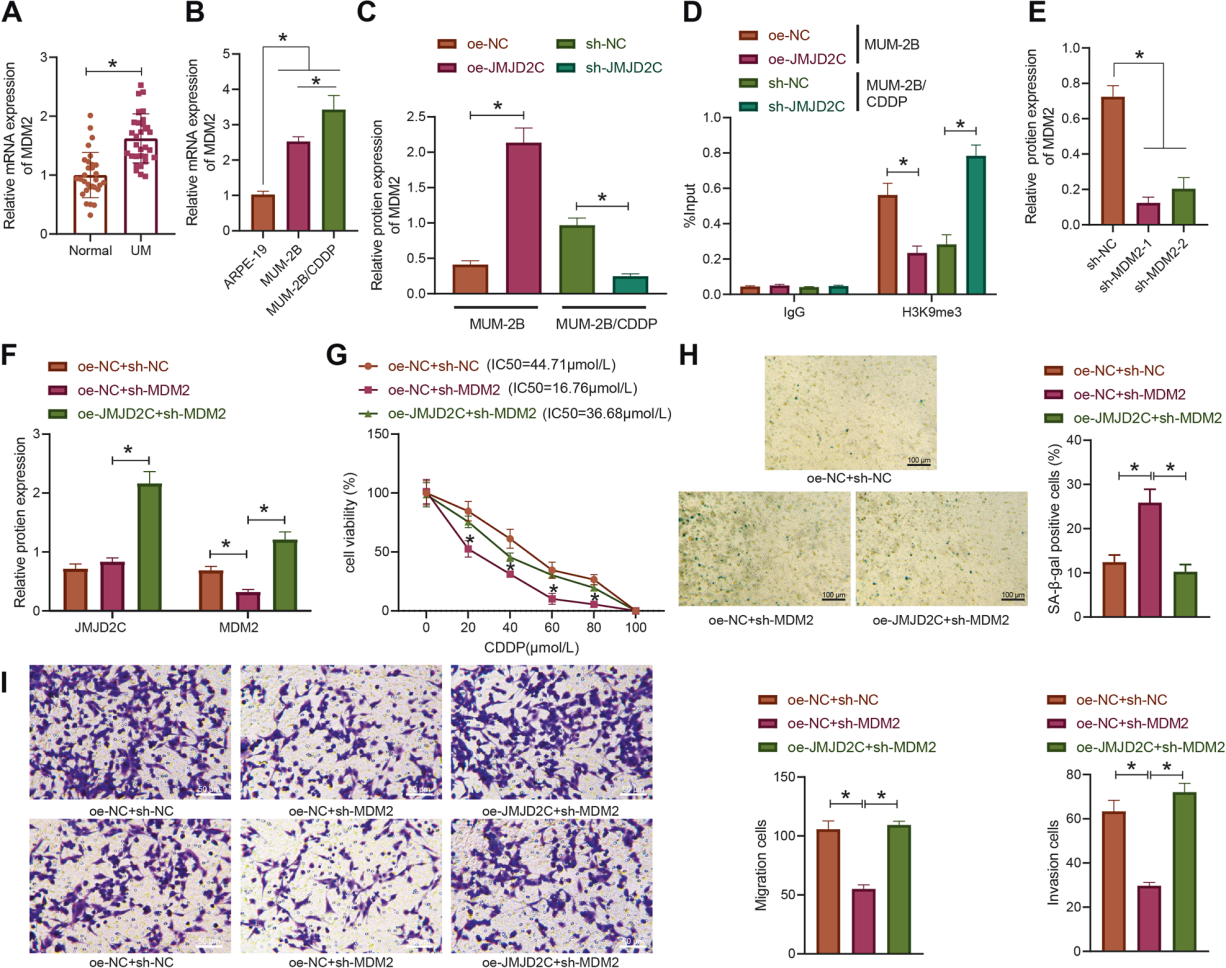
JMJD2C promotes CDDP resistance in uveal melanoma cells by removing histone methylation of MDM2 promoter. **A** The expression of MDM2 in uveal melanoma tissues as determined by RT-qPCR (uveal melanoma = 32, normal = 30). **B** The expression of MDM2 in normal uveal epithelial cells ARPE-19, uveal melanoma cells MUM-2B, and drug-resistant cell line MUM-2B/CDDP as determined by RT-qPCR. **C** The expression of MDM2 in MUM-2B/CDDP cells after JMJD2C silencing and MUM-2B cells after JMJD2C overexpression as measured by Western blot assay. **D** Enrichment degree of H3K9me3 in MDM2 promoter region in MUM-2B/CDDP cells after JMJD2C silencing and MUM-2B cells after JMJD2C overexpression as determined by ChIP. **E** The expression of MDM2 silenced by shRNAs in MUM-2B/CDDP cells as measured by Western blot assay. **F** The protein expression of JMJD2C and MDM2 in MUM-2B/CDDP cells treated with sh-MDM2 or combined with oe-JMJD2C as measured by Western blot assay. **G** The viability and IC50 of MUM-2B/CDDP cells treated with sh-MDM2 or combined with oe-JMJD2C as assessed by CCK-8 assay. **H** The senescence rate of MUM-2B/CDDP cells treated with sh-MDM2 or combined with oe-JMJD2C as assessed by SA-β-gal staining. **I** The migration and invasion abilities of MUM-2B/CDDP cells treated with sh-MDM2 or combined with oe-JMJD2C as assessed by Transwell assay. * *p* < 0.05. Cell experiments were repeated three times independently.

In the next experiment, we moved to elucidate the mechanism of JMJD2C in the CDDP resistance of uveal melanoma. RT-qPCR results demonstrated that the expression of MDM2 was noticeably higher in uveal melanoma tissues than in the normal uveal tissues (Fig. 3A). Consistently, the expression of MDM2 was also increased in MUM-2B and MUM-2B/CDDP cells versus the ARPE-19 cells, of which MUM-2B/CDDP cells showed much higher expression of MDM2 (Fig. 3B).

In attempt to further study the regulatory mechanism between JMJD2C and MDM2, JMJD2C was overexpressed in MUM-2B cells and silenced in MUM-2B/CDDP cells. Western blot assay results suggested that the expression of MDM2 was significantly increased in MUM-2B cells after overexpression of JMJD2C, but it was decreased in MUM-2B/CDDP cells following JMJD2C silencing (Figs. 3C and S4B), suggesting that JMJD2C can positively regulate the expression of MDM2. Additionally, the enrichment of H3K9me3 in MDM2 promoter region was detected by ChIP. It was found that after overexpression of JMJD2C in MUM-2B cells, the enrichment of H3K9me3 in MDM2 promoter had a marked decrease, which was reversed upon silencing of JMJD2C in MUM-2B/CDDP cells (Fig. 3D), suggesting that JMJD2C can promote MDM2 expression by suppressing H3K9me3 in the MDM2 promoter region.

Next, MUM-2B/CDDP cells were subjected to different treatments to study the effect of JMJD2C on drug resistance of uveal melanoma cells by regulating MDM2. Western blot assay results (Figs. 3E, F and S4C, D) showed that the expression of MDM2 was markedly repressed in response to manipulation with sh-MDM2, which was reversed following additional oe-JMJD2C treatment. Moreover, the results of CCK-8 assay (Fig. 3G) revealed that MDM2 knockdown contributed to significantly decreased cell viability and IC50, which effects were negated by enhanced JMJD2C expression. According to the results of SA-β-gal staining and Transwell assay (Fig. 3H, I), MDM2 loss-of-function brought about a marked increase in the senescence rate of MUM-2B/CDDP cells, accompanied by reduced migration and invasion abilities. However, oe-JMJD2C counteracted the effects of sh-MDM2 on the aforesaid abilities of MUM-2B/CDDP cells. Overall, JMJD2C can promote the expression of MDM2 by removing histone methylation of MDM2 promoter, thus promoting CDDP resistance in uveal melanoma cells.

### MDM2 degraded p53 through ubiquitination and promoted the expression of IL5RA

**Fig. 4:**
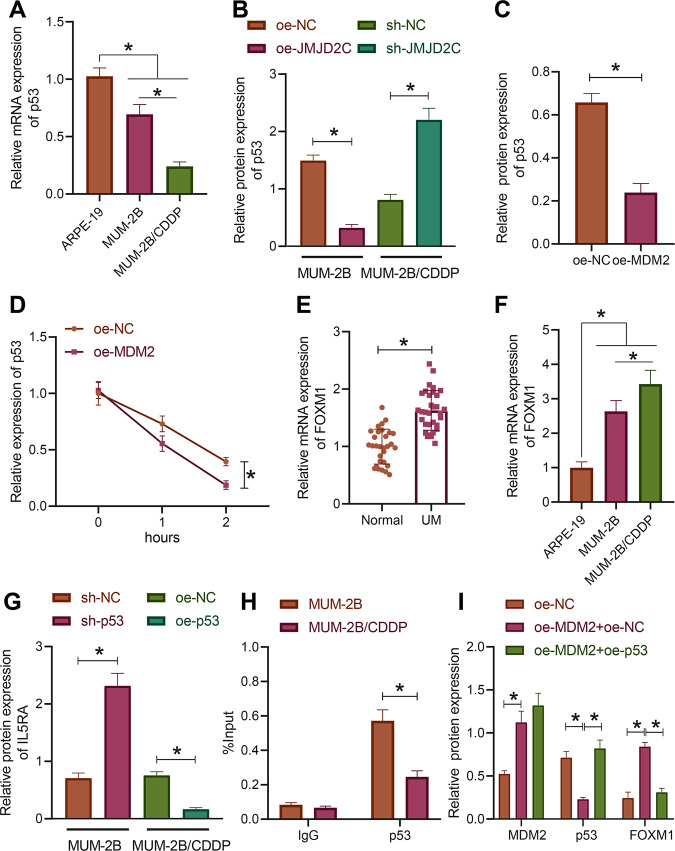
MDM2 degrades p53 through ubiquitination and promotes the expression of IL5RA. **A** The expression of p53 in normal uveal epithelial cells ARPE-19, uveal melanoma cells MUM-2B, and drug-resistant cell line MUM-2B/CDDP as determined by RT-qPCR. **B** The expression of p53 in MUM-2B/CDDP cells upon JMJD2C silencing and MUM-2B cells upon JMJD2C overexpression as measured by Western blot assay. **C** The expression of p53 in MUM-2B cells upon MDM2 overexpression as measured by Western blot assay. **D** The stability of p53 protein after CHX treatment. **E** The expression of IL5RA in uveal melanoma tissues as determined by RT-qPCR (uveal melanoma = 32, normal = 30). **F** The expression of IL5RA in normal uveal epithelial cells ARPE-19, uveal melanoma cells MUM-2B and drug-resistant cell line MUM-2B/CDDP as determined by RT-qPCR. **G** IL5RA expression in MUM-2B/CDDP cells upon p53 overexpression and MUM-2B cells upon p53 silencing measured by Western blot assay. **H** The enrichment of p53 in the IL5RA promoter region in MUM-2B/CDDP cells and MUM-2B cells analyzed by ChIP. **I** The expression of MDM2, p53, and IL5RA in MUM-2B cells treated with oe-MDM2 or combined with oe-p53 as measured by Western blot assay. * *p* < 0.05. Cell experiments were repeated three times independently.

We then proceeded to analyze the downstream mechanism of MDM2 participating in the CDDP resistance in uveal melanoma. RT-qPCR detection results indicated reductions in the expression of p53 in MUM-2B and MUM-2B/CDDP cells versus the normal uveal epithelial cells ARPE-19, with MUM-2B/CDDP cells showing much lower expression of p53 (Fig. 4A). Additionally, Western blot assay results showed a decline in the expression of p53 in MUM-2B cells overexpressing JMJD2C while an enhancement of that was noted in MUM-2B/CDDP cells following JMJD2C silencing (Figs. 4B and S4E). The above results indicate that p53 is poorly expressed in uveal melanoma cells MUM-2B and drug-resistant cells MUM-2B/CDDP, and that JMJD2C may negatively regulate p53 in uveal melanoma cells.

Western blot assay results showed that after overexpression of MDM2, the expression of p53 decreased significantly (Figs. 4C and S4F). Co-IP demonstrated that after MDM2 overexpression in the cells added with MG132, the ubiquitination level of p53 was increased (Fig. S3). Furthermore, the protein stability of p53 was markedly impeded after overexpression of MDM2 (Figs. 4D and S4G). These results suggest that E3 ubiquitin ligase MDM2 can promote the ubiquitination degradation of p53 and thus inhibit its expression.

RT-qPCR results displayed remarkably higher expression of IL5RA in uveal melanoma tissues than in normal uveal tissues (Fig. 4E, F). Compared with ARPE-19 cells, the expression of IL5RA was markedly increased in MUM-2B and MUM-2B/CDDP cells; the expression of IL5RA was significantly higher in MUM-2B/CDDP cells than that in MUM-2B cells. The above-mentioned results demonstrate that IL5RA highly expresses in uveal melanoma and correlates with drug resistance.

As shown in Figs. 4G and S4H, silencing of p53 in MUM-2B cells induced an increase in the expression of IL5RA while upon overexpression of p53 in MUM-2B/CDDP cells, the expression of IL5RA was reduced. ChIP test results showed that in MUM-2B and MUM-2B/CDDP cells, p53 could be significantly enriched in the IL5RA promoter region, with a more obvious enrichment noted in MUM-2B cells (Fig. 4H). It is thus supposed that p53 can transcriptionally repress the expression of IL5RA in uveal melanoma cells.

MUM-2B cells were subjected to different treatments. The protein expression patterns of MDM2 and IL5RA were notably increased while that of p53 was markedly decreased after manipulation with oe-MDM2 + oe-NC. Re-expression of p53 failed to lead to significant difference in MDM2 expression, but appreciably reduced the IL5RA protein level (Figs. 4I and S4I). Taken together, MDM2 can degrade p53 and promote the expression of IL5RA through ubiquitin proteasome pathway.

### MDM2 degraded p53 through ubiquitination to promote IL5RA expression, thereby promoting CDDP resistance in uveal melanoma cells

**Fig. 5:**
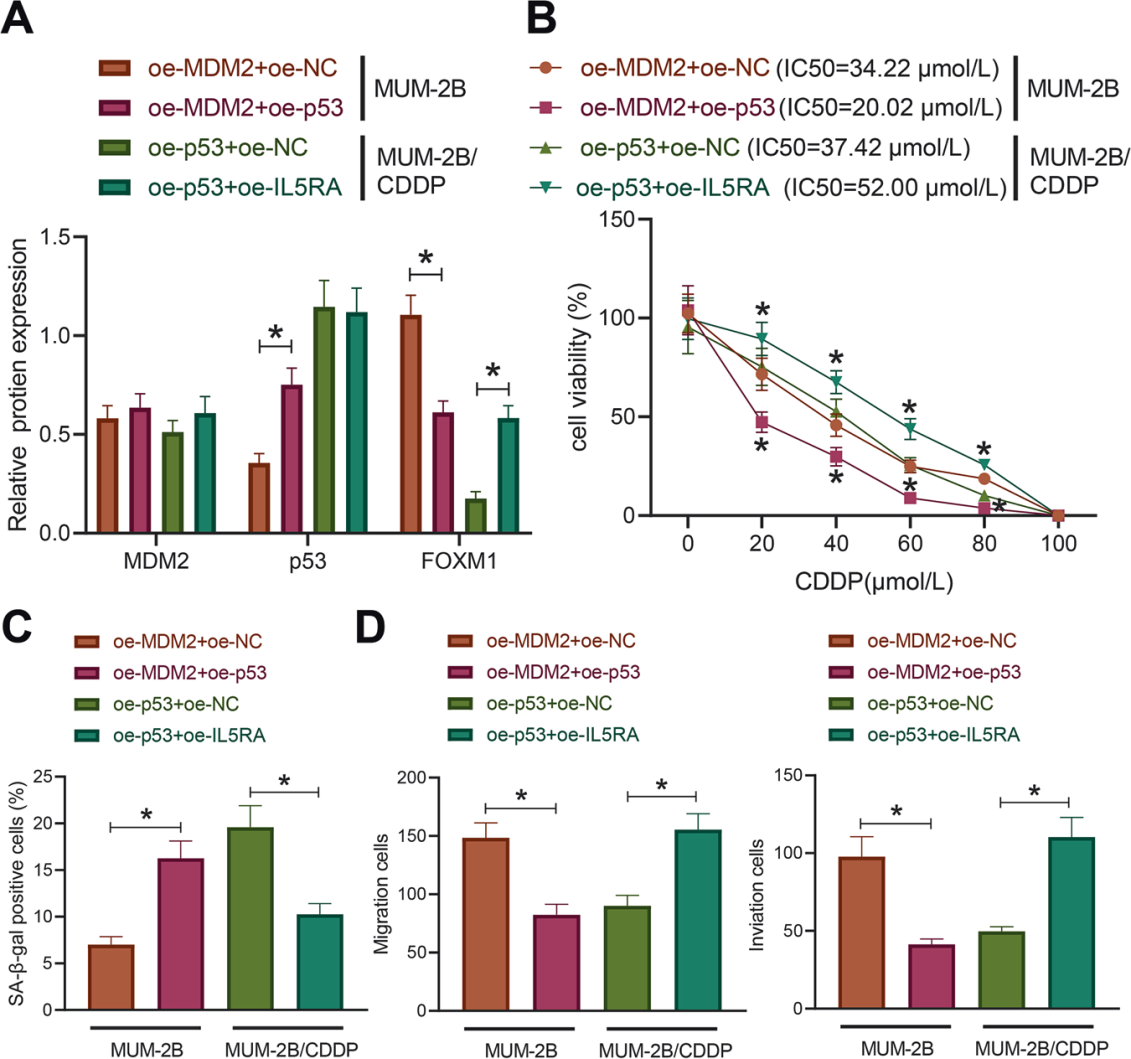
MDM2 promotes CDDP resistance in uveal melanoma cells through regulation of the p53/IL5RA axis. **A** The expression of MDM2, p53, and IL5RA in MUM-2B and MUM-2B/CDDP cells with different treatments as measured by Western blot assay. **B** The viability and IC50 of MUM-2B and MUM-2B/CDDP cells with different treatments as examined by CCK-8 assay. **C** The senescence rate of MUM-2B and MUM-2B/CDDP cells with different treatments as assessed by SA-β-gal staining. **D** The migration and invasion abilities of MUM-2B and MUM-2B/CDDP cells with different treatment as examined by Transwell assay. * *p* < 0.05. Cell experiments were repeated three times independently.

For further exploration of the effect of MDM2-mediated p53/IL5RA on the drug resistance in uveal melanoma cells, we treated the MUM-2B cells with oe-MDM2 + oe-NC or oe-MDM2 + oe-p53 and MUM-2B/CDDP cells with oe-p53 + oe-NC or oe-p53 + oe-IL5RA in combination. The expression of p53 was successfully restored by oe-p53 at protein level in MUM-2B cells in the presence of MDM2, leading to a notably reduced protein level of IL5RA (Figs. 5A, B and S4J). In MUM-2B/CDDP cells, restoration of the IL5RA expression had no significant effect on the protein expression pattern of p53 (Figs. 5A, B and S4J). In the presence of MDM2, p53 re-expression significantly decreased the MUM-2B cell viability and IC50. In the presence of p53, restoration of IL5RA contributed to markedly increased MUM-2B/CDDP cell viability and IC50.

The results of SA-β-gal staining and Transwell assay (Fig. 5C, D) showed that oe-p53 evoked a significant increase in the senescence rate of MUM-2B cells overexpressing MDM2 while impeding their migration and invasion abilities. On the contrary, oe-IL5RA significantly reduced the senescence rate of MUM-2B/CDDP cells overexpressing p53 and boosted their migration and invasion abilities. In conclusion, MDM2 can degrade p53 *via* ubiquitination and promote the expression of IL5RA, thus promoting CDDP resistance in uveal melanoma cells.

### JMJD2C accelerated the growth of uveal melanoma in nude mice *via* regulation of the MDM2/p53/IL5RA axis

**Fig. 6:**
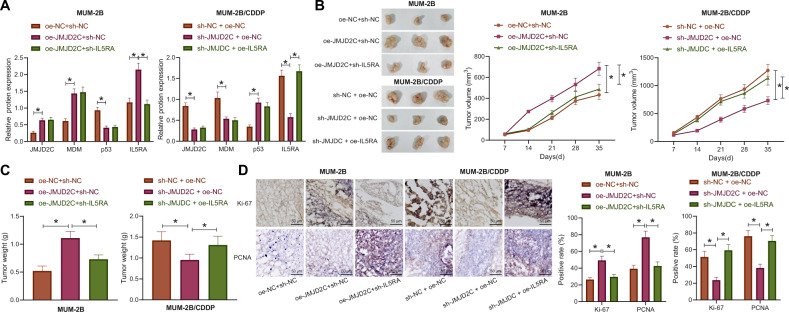
JMJD2C accelerates the growth of uveal melanoma and increases CDDP resistance in nude mice *via* regulation of the MDM2/p53/IL5RA axis. **A** The expression of JMJD2C, MDM2, p53, and IL5RA in tumors derived from MUM-2B cells and MUM-2B/CDDP cells as measured by Western blot assay. **B** Representative images showing xenografted tumors in derived from MUM-2B cells and MUM-2B/CDDP cells and tumor growth curves. **C** The statistical plot for tumor weight of MUM-2B- and MUM-2B/CDDP-implanted nude mice. **D** Positive rates of Ki67 and PCNA in MUM-2B cell- and MUM-2B/CDDP cell-derived tumor tissues of nude mice as detected by immunohistochemistry. Scale bar = 50 μm. *n* = 8 for mice following each treatment. * *p* < 0.05.

The study focus was shifted onto the effect of JMJD2C-mediated MDM2/p53/IL5RA axis on the growth of uveal melanoma and the resistance to CDDP in vivo, we carried out nude mouse tumorigenicity assay. The results of Western blot assay exhibited increases in the expression patterns of JMJD2C, MDM2, and IL5RA yet a decrease in that of p53 in the tumor tissues of nude mice xenografted with the MUM-2B cells that had stably transfected with oe-JMJD2C + sh-NC, as compared to those xenografted with the MUM-2B cells that had stably transfected oe-NC + sh-NC. Relative to the mice inoculated with oe-JMJD2C + sh-NC-treated MUM-2B cells, the mice inoculated with oe-JMJD2C + sh-IL5RA-treated MUM-2B cells showed insignificant change in the expression of JMJD2C, MDM2, and p53 but a reduced IL5RA protein level in the tumor tissues (Figs. 6A and S4K). The expression of JMJD2C, MDM2, and IL5RA were noticeably lower while that of p53 was higher in the tumors derived from the MUM-2B/CDDP cells stably transfected with sh-JMJD2C + oe-NC than in the tumors derived from the sh-NC + oe-NC-treated MUM-2B/CDDP cells. No alterations were measured in the expression of JMJD2C, MDM2, and p53 while that of IL5RA was restored in the tumors derived from the sh-JMJD2C + oe-IL5RA-treated MUM-2B/CDDP cells than in the tumors derived from the sh-JMJD2C + oe-NC-treated MUM-2B/CDDP cells (Figs. 6A and S4K).

As shown in Fig. 6B, C, upregulation of JMJD2C markedly increased the volume and weight of MUM-2B cell-derived tumors, while loss of IL5RA contributed to marked decreases in tumor volume and weight in the presence of JMJD2C. JMJD2C deficiency decreased the volume and weight of MUM-2B/CDDP cell-derived tumors while reversed effects were noted upon restoration of IL5RA expression.

Moreover, the positive rates of Ki-67 and PCNA were significantly elevated by JMJD2C gain-of-function in the MUM-2B cell-derived tumors; whereas the elevation of Ki-67 and PCNA positive rates induced by JMJD2C gain-of-function were notably lowered when IL5RA was knocked down (Fig. 6D). In the MUM-2B/CDDP cell-derived tumors, the positive rates of Ki-67 and PCNA were decreased in response to silencing of JMJD2C, which were conversely increased upon restoration of IL5RA (Fig. 6D). Taken together, JMJD2C can facilitate the growth of uveal melanoma in nude mice by mediating the MDM2/p53/IL5RA axis.

## Discussion

Uveal melanoma in advanced stages presents with an unsatisfactory prognosis due to tumor metastasis and drug resistance [17]. We conducted the current study to explore the role of JMJD2C in uveal melanoma and found that JMJD2C contributed to promoting CDDP resistance in uveal melanoma by regulating the MDM2/p53/IL5RA axis.

Importantly, our study unraveled an upregulation of JMJD2C in uveal melanoma and demonstrated its promoting effect on CDDP resistance. In consistency with this finding, a previous study conducted by Yu et al. revealed that in mouse and zebrafish, the enforced expression of JMJD2C could regulate melanoma cell senescence and in vivo tumor growth to augment melanomagenesis driven by Braf-V600E, with H3K9 demethylase activities observed in melanoma cell lines as well as primary human melanoma samples [8]. Of note, several previous studies have unveiled the significance of JMJD2C in the chemotherapy resistance. As suggested by Hamada et al., inhibition of JMJD2C in combination with suppressed lysine-specific demethylase 1 might serve as a potential direction for anticancer chemotherapy [18]. Moreover, it was revealed that JMJD2C could promote CDDP resistance in ovarian cancer cells and thus might serve as a promising target for the treatment of ovarian cancer [16]. Collectively, the aforementioned findings are supportive of our result in regard to the promoting role of JMJD2C in CDDP resistance in uveal melanoma.

In addition, we found in this study that JMJD2C promoted CDDP resistance in uveal melanoma cells *via* histone demethylation of the MDM2 promoter. It is noteworthy that JMJD2C is an important gene accountable for oncogenesis by regulating its downstream target MDM2. JMJD2C could be recruited to the P2 promoter region of MDM2 gene to evoke the demethylation of histone H3 lysine 9. Hence, overexpressed JMJD2C could upregulate MDM2, in a demethylase activity-dependent manner [9]. Notably, mounting evidence has demonstrated the oncogenic role of MDM2 in melanoma. For instance, MDM2 could regulate JMJD6 degradation to diminish H2A.X phosphorylation, thereby resulting in uncontrolled uveal melanoma cell migration [19]. Stabilized MDMX-MDM2 complex by AXL receptor signal inhibited p53 in melanoma, which could reduce the sensitivity of tumor cells to CDDP [20]. Combined with our experimental data, it is rational to conclude that the effect of JMJD2C in CDDP resistance in uveal melanoma cells is promisingly achieved through its upregulation on MDM2.

It was also validated in this study that MDM2 can promote the expression of IL5RA by ubiquitination-dependent degradation of p53 in uveal melanoma cells. In fact, a growing number of studies have highlighted the involvement of MDM2 in melanoma, with the interaction with p53. p53 signaling can be disrupted in uveal melanoma by overexpression of MDM2 [21]. Additionally, it was revealed that inhibition of MDM2 could produce cytotoxic activity in cutaneous melanoma cells, which was achieved by increased p53 stabilization [22]. The mutational status of p53 is a very complicated problem that missense mutations in the Tp53 gene have been widely found in human cancers, which may produce mutant p53 proteins that lose tumor suppressive activities, and some of which exert trans-dominant inhibition on the wild-type counterpart [23]. However, p53 mutation exerts tumor-promoting effects in melanoma by upregulating the oncogenes and downregulating the tumor-suppressor genes. An existing study has reported that TAP73 interacted with MDM2 and mutant p53 to exert anti-tumor activity against neuroblastoma [24]. A negative correlation was revealed between MDM2 and p53 expression in uveal melanoma samples collected in the TCGA and the regulation of MDM2 in p53 expression was demonstrated in this study to be achieved in ubiquitination-dependent manner. Intriguingly, MDM2-mediated ubiquitination and degradation of p53 was found in about 40% of melanomas in the presence of loss or mutation of CDKN2A [25]. Strikingly, Staege and his partners found that IL5RA was overexpressed in Hodgkin’s lymphoma cell lines which were resistant to CDDP [15]. Of note, the regulatory relationship between p53 and IL5RA has been rarely reported. In the current study, our results demonstrated that degraded p53 by MDM2 could upregulate the expression of IL5RA, thereby promoting CDDP resistance in uveal melanoma cell lines.

**Fig. 7:**
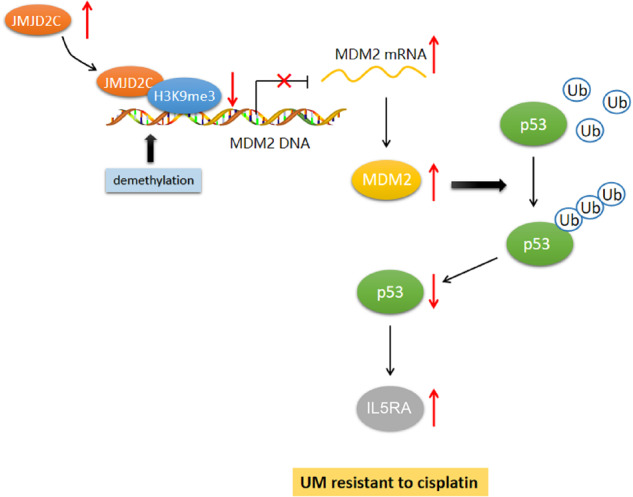
The molecular mechanism by which JMJD2C affects CDDP resistance in uveal melanoma. JMJD2C promotes the expression of MDM2 by removing the H3K9me3 modification of the MDM2 promoter, thus promoting the ubiquitination and degradation of p53, increasing the expression of IL5RA, and ultimately promoting CDDP resistance in uveal melanoma.

From the results obtained in the present study, we came to a conclusion that JMJD2C induced upregulation of MDM2 by removing the H3K9me3 modification of MDM2 promoter, thereby promoting the ubiquitination of p53 and thus increasing the expression of IL5RA, which increases CDDP resistance in uveal melanoma (Fig. 7). The contribution of JMJD2C deficiency to limiting the growth of MUM-2B/CDDP cell-derived tumors was also uncovered in this study. This finding may provide a potential strategy for treatment of uveal melanoma. However, further study is warranted for verifying the clinical feasibility, and the specific regulatory mechanisms regarding the interaction between p53 and IL5RA and the regulator role of IL5RA in uveal melanoma still lack elucidation.

## Materials and methods

### Ethical approval

The study was approved by ethics committee of The Second Hospital of Jilin University and the patients’ informed consent was obtained. The study also follows *Declaration of Helsinki*. All animal experiments were conducted in accordance with the guidelines for the care and use of laboratory animals issued by the National Institutes of Health.

### Clinical sample collection

A total of 32 cases of samples were collected from uveal melanoma patients with complete data who underwent surgery from April 2015 to December 2018 at The Second Hospital of Jilin University. Meanwhile, 30 cases of samples were collected from patients who underwent ophthalmectomy at The Second Hospital of Jilin University during the same period but had normal uveal membrane.

### Cell treatment

The uveal melanoma cell line MUM-2B and normal uveal membrane epithelial cell line ARPE-19, which were purchased from American Type Culture Collection (ATCC, Manassas, VA), were cultured in Roswell Park Memorial Institute-1640 (RPMI-1640) (PM150110, Procell Life Science & Technology, Wuhan, China) at 37 °C with 5% CO_2_ for 48 h. Cells in the logarithmic growth were used in the experiment. CDDP was purchased from Sigma-Aldrich (St Louis, MO; C2210000). The CDDP-resistant cell line (MUM-2B/CDDP) was constructed by treating MUM-2B cells with continuously increased CDDP concentration. The cells were followingly cultured in the medium containing low concentration of CDDP. A drug resistance assessment of the MUM-2B/CDDP cells was conducted before each use.

MUM-2B cells were manipulated with negative control plasmid of overexpression vector (oe-NC), JMJD2C overexpression plasmid (oe-JMJD2C), MDM2 overexpression plasmid (oe-MDM2), negative control shRNA (sh-NC), and shRNA targeting p53 (sh-p53) alone, or with oe-NC + sh-NC, oe-MDM2 + oe-NC, and oe-MDM2 + p53 overexpression plasmid (oe-p53) in combination.

The MUM-2B/CDDP cells were introduced with oe-NC, oe-JMJD2C, sh-NC, shRNA targeting JMJD2C (sh-JMJD2C), and shRNA targeting MDM2 (sh-MDM2) alone, or with oe-NC + sh-NC, oe-NC + sh-MDM2, oe-JMJD2C + sh-MDM2, oe-p53 + oe-NC, oe-p53 + IL5RA overexpression plasmid (oe-IL5RA), oe-JMJD2C + sh-NC, oe-JMJD2C + shRNA targeting IL5RA (sh-IL5RA) in combination, which was carried out based on the protocols of Lipofectamine 2000 kit (1166819, Thermo Fisher Scientific, Rockford, IL). Next, 4 μg of target plasmids and 10 μL Lipofectamine 2000 were respectively diluted with 250 μL serum-free Opti-MEM medium (Gibco, Carlsbad, CA), the two of which were mixed evenly and allowed to rest for 20 min. Later, the mixture was added to the culture wells; the previous medium was renewed with a complete medium after 6 h. After 48 h of further culture, the cells were harvested for determining the transfection efficiency qualified for subsequent experiments.

### RT-qPCR

TRIzol reagent (Invitrogen, Carlsbad, CA) was utilized for the extraction of total RNA sourced from tissues and cells. A Nanodrop2000 micro-ultraviolet spectrophotometer (1011U, NanoDrop Technologies Inc., Wilmington, DE) was adopted for measuring the RNA concentration and purity. According to the instructions of the PrimeScript RT reagent kit (RR047A, Takara Holdings Inc., Kyoto, Japan), RNA was reverse-transcribed into complementary DNA. Primers for YY1, MEF2A, STAT3, NR3C1, JMJD2C, MDM2, p53, and IL5RA were designed and synthesized by Takara (Table S3). Real-time fluorescent qPCR was operated with the assistance of an ABI7500 qPCR instrument (ABI Company, Oyster Bay, NY). With glyceraldehyde-3-phosphate dehydrogenase (GAPDH) serving as loading control, the relative transcription expression of target genes was quantitated utilizing 2^−ΔΔCT^ method.

### Western blot assay

The total protein was extracted by radio-immunoprecipitation assay (RIPA) lysis buffer containing phenylmethylsulfonyl fluoride (PMSF; P0013C, Beyotime, Shanghai, China). After bicinchoninic acid protein quantitation, 50 µg protein was dissolved in 2 × sodium dodecyl sulfate sample buffer and boiled at 100 °C for 5 min. The above samples were subjected to sodium dodecyl sulfate polyacrylamide gel electrophoresis, and the protein was transferred to a polyvinylidene fluoride membrane by wet transfer and sealed with 5% skim milk powder at ambient temperature 1 h. The membrane was mixed with diluted rabbit antibodies against JMJD2C (1: 2000, ab226480), MDM2 (1: 1200, ab260074), p53 (1: 1000, ab183544), IL5RA (1: 1000, ab134935), and GAPDH (ab9485, 1: 2500) sourced from Abcam (Cambridge, UK). The membrane was incubated with horseradish peroxidase-labeled goat anti-rabbit against immunoglobulin G (IgG) (H&L; ab97051, 1: 2000, Abcam) for 1 h. The same amounts of liquid A and liquid B in the enhanced chemiluminescence detection kit (BB-3501, Amersham, Little Chalfont, UK) were collected, mixed in darkness, and dropped onto the film. The samples were photographed in the Bio-Rad image analysis system (Bio-Rad Laboratories, Hercules, CA), and analyzed using the Quantity One v4.6.2 software. The relative protein expression was expressed by the ratio between the gray value of the corresponding protein band to that of GAPDH protein band.

### ChIP

MUM-2B and MUM-2B/CDDP cells were obtained and fixed with 1% formaldehyde for 10 min at room temperature when the cell confluency reached 70–80%. DNA and protein were fixed and crosslinked, after which they were ultrasonicated into fragments. Following centrifugation at 4 °C and 13,000 × *g*, the obtained supernatant was placed into two tubes, which were respectively incubated with negative control rabbit anti-IgG (1: 100, ab172730, Abcam) and antibody to H3K9me3 (1: 100, ab176916, Abcam, UK) or p53 (1:100, ab1101, Abcam) overnight at 4 °C. Agarose/Sepharose was used to precipitate the endogenous DNA protein complex. After a short time of centrifugation, the supernatant was removed, and the non-specific complex was washed, followed by de-crosslinking overnight at 65 °C. The DNA fragments were purified by phenol/chloroform and retrieved. The enrichment of JMJD2C in the promoter region of MDM2 gene and that of p53 in the IL5RA promoter region were detected by PCR. The MDM2 promoter primer sequences were F: 5′-AGCACTGAGTCTATTAGAAACCCC-3′ and R: 5′-TCCCAGCCTACCAGAAAGGA-3′. IL5RA promoter primer sequences were: 5′-CCTTCTCTTCCAGCTTTGCAC-3′ and R: 5′-AGTCCTTGACGCACACAACA-3′.

### co-IP

MUM-2B/CDDP cells were lysed on ice with IP lysate containing protease inhibitor MG-132. Next, 1 mg protein was harvested from each sample, the volume of which was made equal to that of IP lysate. p53 monoclonal antibody was added to the cells for IP, followed by incubation at 4 °C overnight. In the morning of the next day, 20 μL protein A + G beads were added to the cells for incubation for 2 h. After centrifugation, the supernatant was removed carefully, followed by addition of 2 × loading buffer (20 μL/well). The gained samples were adopted for subsequent sodium dodecyl sulfate polyacrylamide gel electrophoresis and Western blot assay. The antibodies from Abcam utilized in this test were anti-IgG (1: 100, ab172730), anti-Ub (1: 100, ab7780), and anti-p53 (1: 100, ab183544).

### Protein stability test

To assess the stability of p53 protein, MDM2 was overexpressed in MUM-2B/CDDP cells and incubated with 20 μg/mL CHX (protein synthesis inhibitor, sourced from Sigma-Aldrich). MUM-2B/CDDP cells were lysed with RIPA lysis buffer (P0013B, Beyotime) and centrifuged at 12,000 × *g* for protein extraction, followed by protein quantitation utilizing Western blot assay. The expression of p53 was quantified by ImageJ and standardized to GAPDH.

### Cisplatin treatment and CCK-8 assay

CCK-8 kits (CK04, Dojindo, Kumamoto, Japan) were utilized for evaluating the proliferative potential of MUM-2B and MUM-2B/CDDP cells. MUM-2B and MUM-2B/CDDP cells in the logarithmic growth phase were seeded in a 96-well plate (1 × 10^4^ cells per well) and cultured for 1, 2, 3, 4, and 5 days. During this period, 10 μL CCK-8 reagent was loaded at the same time every day, and incubation was made at 37 °C for 3 h. The OD value of each well at 450 nm was measured using a microplate reader. The values were proportional to the number of proliferated cells in the medium, and the cell growth curve was plotted.

MUM-2B and MUM-2B/CDDP cells in logarithmic growth phase were seeded in a 96-well plate (1 × 10^4^ cells per well) for 24 h. After 24 h, cells were exposed to different concentrations of CDDP. After 48 h, 10 μL CCK-8 reagent was loaded for another 3 h of incubation at 37 °C. The OD value of each well at 450 nm wavelength was recorded on a Microplate reader and finally presented in the cell growth curve. IC50 refers to the concentration of cisplatin inhibiting the cell viability by 50%. A higher IC50 value indicates higher resistance to chemotherapy [26].

### SA-β-gal staining

The cell culture medium in the six-well plate was aspirated, and 1 mL of SA-β-gal staining fixative was added to each well for 20 min of fixation at ambient temperature. Next, 1 mL of staining working solution was added to each well. The six-well plate was sealed with sealing film to prevent evaporation and incubated overnight at 37 °C. The following day, cells were visualized and counted under microscopical observation.

### FACS analysis

The concentration of MUM-2B and MUM-2B/CDDP cell suspension was adjusted to 1–5 × 10^6^ cells/mL. According to the instructions of Cell Cycle Staining Kit (70-CCS012, MultiSciences(Lianke) Biotechnology Corporate Limited, Hangzhou, Zhejiang, China), the cells were stained with PI and collected. Following PBS washing, the cells were mixed with 1 mL DNA Staining solution and 10 μL Permeabilization solution by shaking for 5–10 s, and incubated for 30 min at ambient temperature in the dark. Finally, a flow cytometer was adopted to detect cell cycle.

### Transwell assay

At 4 °C, 200 μL Matrigel (YB356234, Yubo, Shanghai, China) was diluted with 200 μL serum-free medium. Next, 50 μL of the diluted mixture was added into each Transwell chamber, and incubated for 2–3 h until the gel became solid. Subsequently, 200 μL cell suspension was loaded into each well of the apical chamber, which was incubated together with 800 μL conditioned medium containing 20% FBS in the basolateral chamber at 37 °C for 24 h. The Transwell plate was taken out and the cells on the upper surface were removed with cotton balls. The cells inside the Transwell plate were fixed with formaldehyde for 10 min, stained with 0.1% crystal violet, and allowed to rest at room temperature for 30 min. The cells were observed, photographed, and counted under an inverted microscope. Matrigel was not used in the migration assay, and the incubation time was 24 h. Cells in at least four areas were randomly selected under a microscope selected for cell counting.

### Tumorigenesis in nude mice

Forty-eight specific-pathogen-free BALB/c nude mice (aged 6 weeks, 15–18 g) were purchased from SLAC Company (Changsha, China). The mice were subcutaneously injected *via* left posterior abdomen, with suspension (2 × 10^6^ cells/mL) containing MUM-2B cells stably transfected with oe-NC + sh-NC, oe-JMJD2C + sh-NC, and oe-JMJD2C + sh-IL5RA, or MUM-2B/CDDP cells stably transfected with sh-NC + oe-NC, sh-JMJD2C + oe-NC, and sh-JMJD2C + oe-IL5RA (8 mice following each treatment) to construct a subcutaneous xenotransplanted tumor model. After one week, 3.0 mg/kg CDDP [27] was intraperitoneally injected into the nude mice every 3 days. On the 7th, 14th, 21st, 28th, and 35th days after inoculation, the tumor growth was observed and the data were recorded. *V* (mm^3^) = *A*^2^ × *B*/2 (*A* is the long diameter and *B* is the short diameter). On the 36th day of culture, the mice were euthanized by neck dislocation, and the tumor tissues were isolated. The tumor weight was weighed with a balance, and the protein was extracted from the transplanted tumor tissues for Western blot assay and immunohistochemistry.

### Immunohistochemistry

Paraffin-embedded sections of tumor tissues were taken for immunohistochemistry. The samples were washed with tap water for 2 min, 3% methanol for 20 min, distilled water for 2 min, and 0.1 M PBS for 3 min. The samples were subjected to antigen retrieval and cooled down with tap water; normal goat serum sealing solution (C-0005, Shanghai Haoran Biotechnology Co., Ltd., Shanghai, China) was added to the tissue slices, which were then allowed to rest at ambient temperature for 20 min. The slices were then probed utilizing primary antibodies against Ki67 (1: 200, ab15580, Abcam) and PCNA (1100, ab92552, Abcam) overnight at 4 °C. Re-probing was conducted with secondary antibody goat anti-rabbit against IgG (1500, ab150077) at 37 °C for 20 min and with horseradish peroxidase-tagged streptavidin (0343-10000U, Imunbio, Beijing, China) at 37 °C for 20 min. Following color development by 3,3-diaminobenzidine (ST033, Whiga, Guangzhou, China), the cells were counterstained with hematoxylin (PT001, Bogoo Biotechnology Co., Ltd., Shanghai, China) for 1 min and immediately reacted with ammonia to return blue, followed by dehydration, clearing, and mounting. Finally, microscopic images were captured in 5 high-power randomly selected visual fields from each section, with 100 cells in each field; positive cells < 10% indicated negative, positive cells ≥ 10% and < 50% positive, and positive cells > 50% strong positive.

### Statistical analysis

Data were processed by SPSS 19.0 statistical software (IBM Corp. Armonk, NY). The measurement data, obtained from three independent experiments, were expressed as mean ± standard deviation. Data between two groups were compared utilizing unpaired *t*-test, and those among multiple groups by one-way analysis of variance (ANOVA) combined with Tukey’s post hoc test. Data comparison at different time points between multiple groups was achieved using repeated measures ANOVA, followed by Tukey’s post hoc test. *p* < 0.05 indicates the statistically significant difference.

## Supplementary information

Figure S1

Figure S2

Figure S3

Figure S4

Supplementary figure legends

Supplementary tables

Original Data File

author-contribution

## Data Availability

The authors confirm that the data supporting the findings of this study are available within the article.
