# Supplementary figures and images for: JMJD2C mediates the MDM2/p53/IL5RA axis to promote CDDP resistance in uveal melanoma
Source: Cell Death Discov. 2022 Apr 25;8:227. doi: 10.1038/s41420-022-00949-y (PMC9039082; doi:10.1038/s41420-022-00949-y)

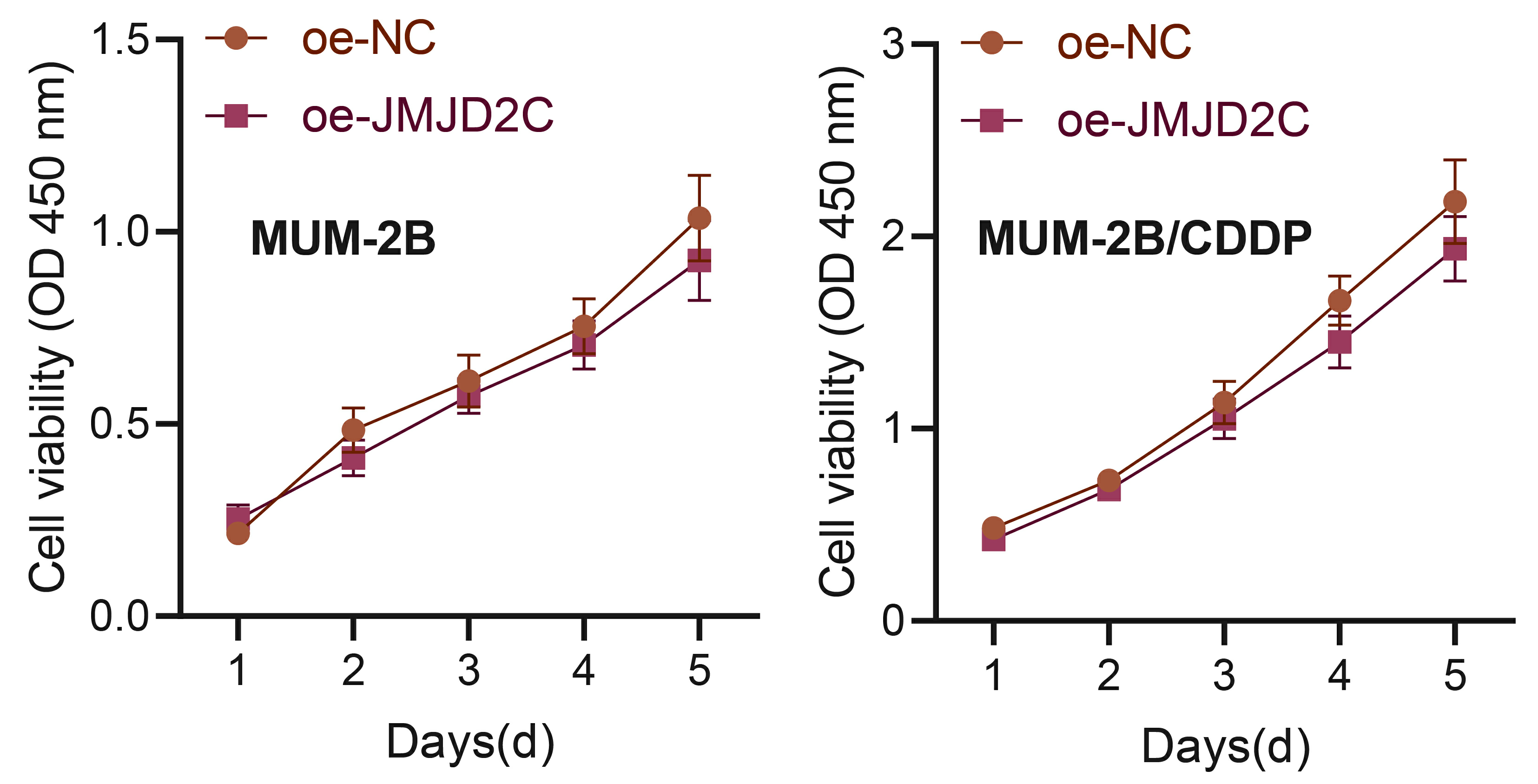

Supplement: Supplementary file 1 — Figure S1 [file 41420_2022_949_MOESM1_ESM.tif]

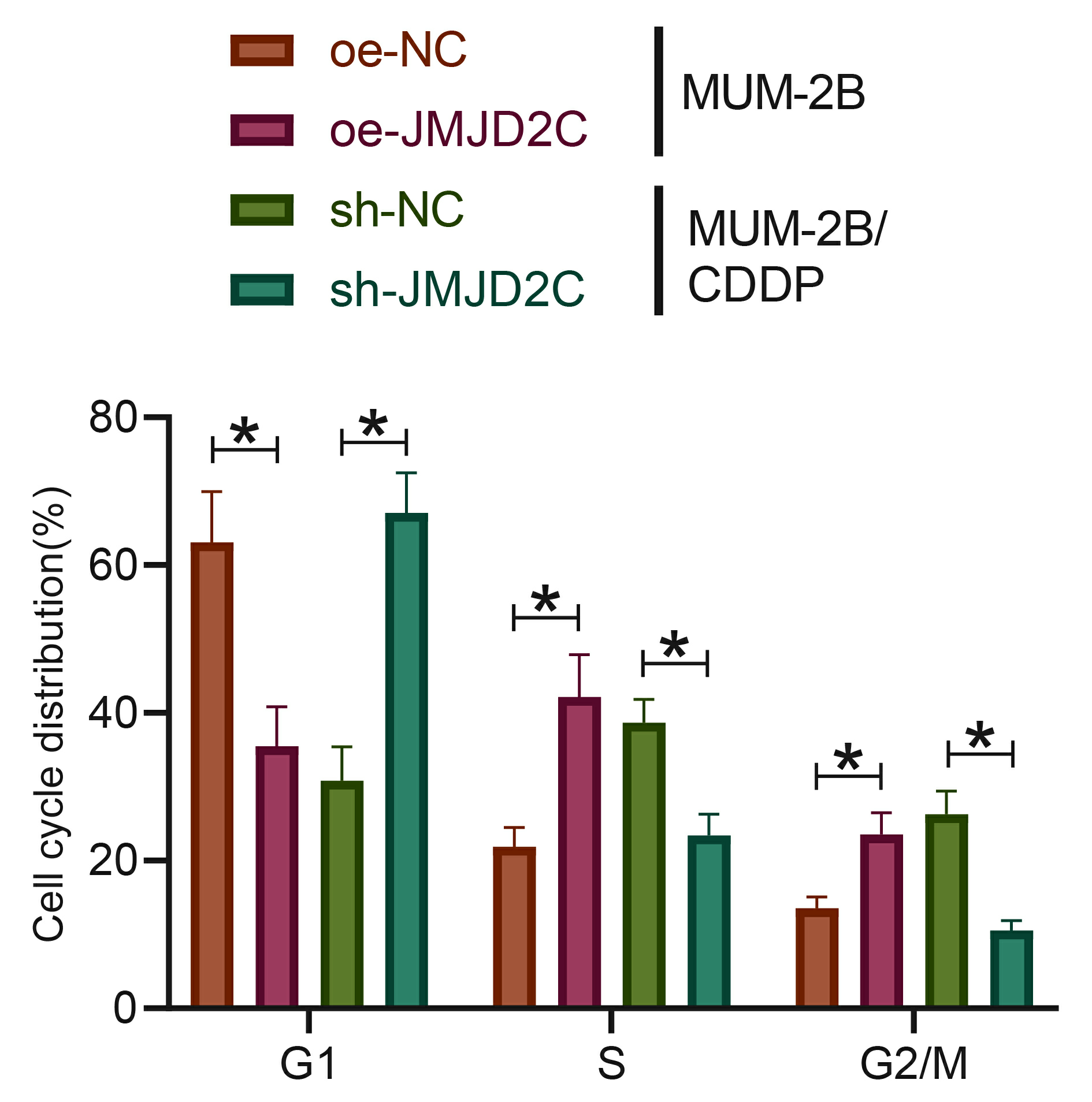

Supplement: Supplementary file 2 — Figure S2 [file 41420_2022_949_MOESM2_ESM.tif]

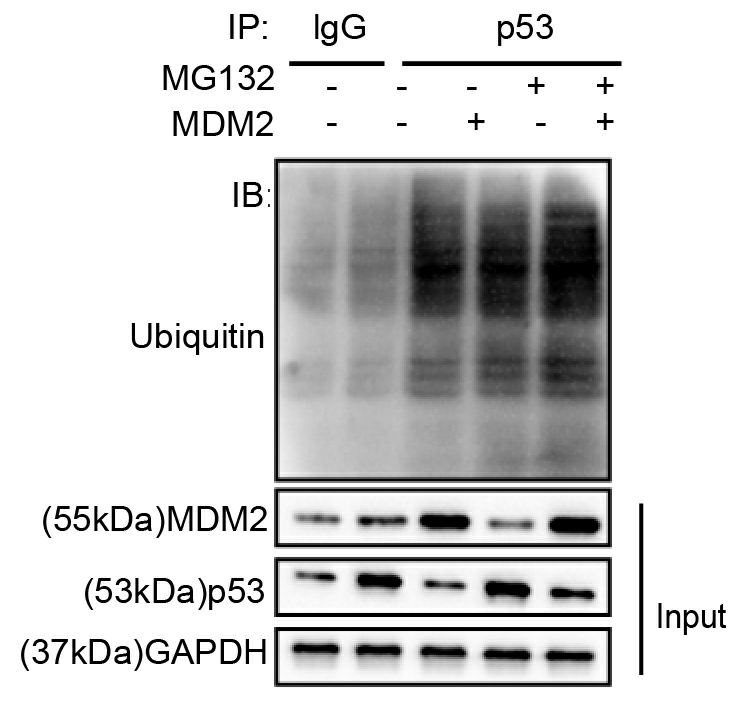

Supplement: Supplementary file 3 — Figure S3 [file 41420_2022_949_MOESM3_ESM.tif]

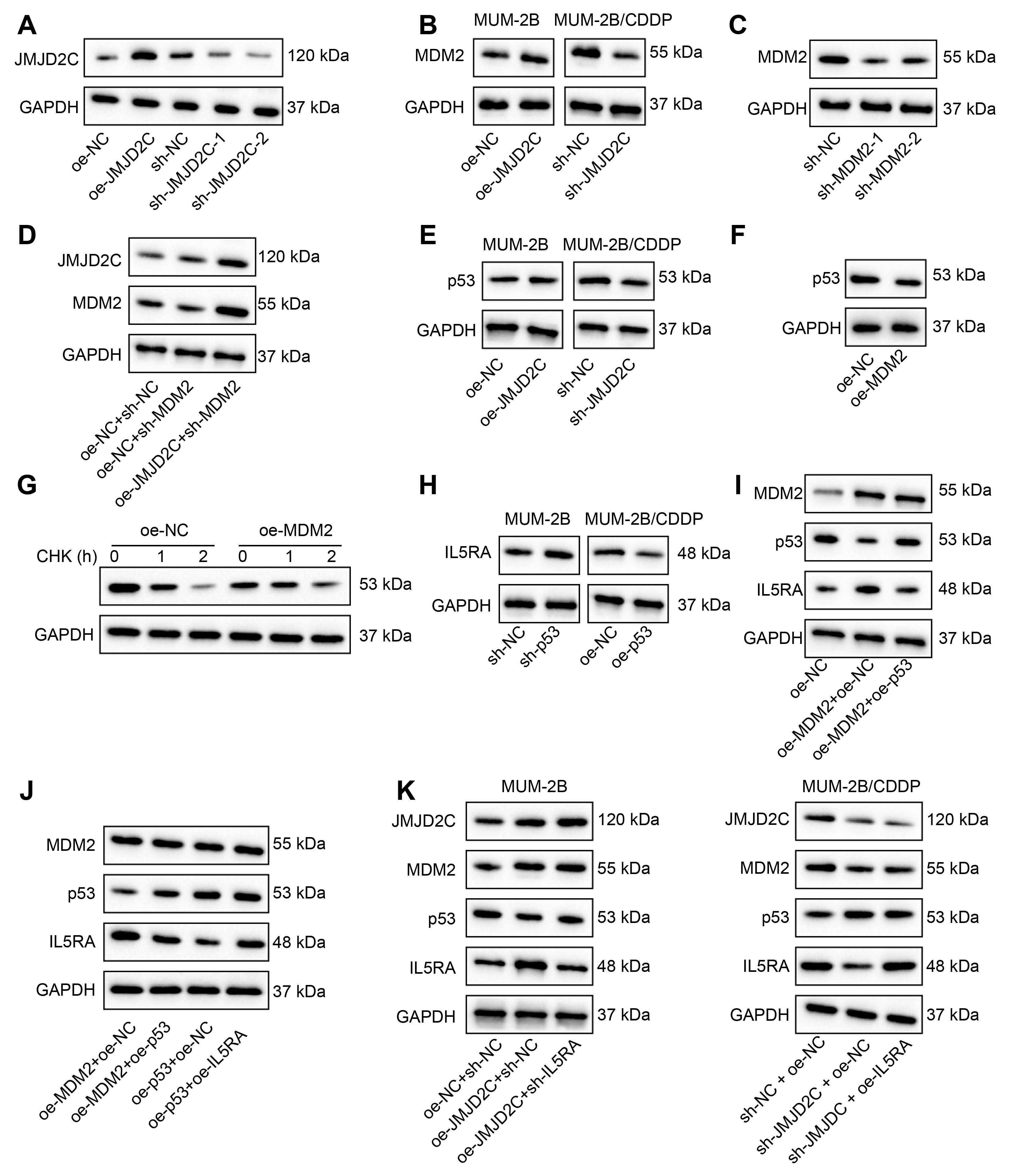

Supplement: Supplementary file 4 — Figure S4 [file 41420_2022_949_MOESM4_ESM.tif]
