# Supplementary material for: JMJD2C mediates the MDM2/p53/IL5RA axis to promote CDDP resistance in uveal melanoma
Source: Cell Death Discov. 2022 Apr 25;8:227. doi: 10.1038/s41420-022-00949-y (PMC9039082; doi:10.1038/s41420-022-00949-y)
Supplement: Supplementary file 5 — Supplementary figure legends [file 41420_2022_949_MOESM5_ESM.docx]

**Fig. S1.** Viability of MUM-2B cells upon JMJD2C overexpression or MUM-2B/CDDP cells upon JMJD2C silencing without CDDP treatment.

**Fig. S2.** Cell cycle distribution of MUM-2B cells upon JMJD2C overexpression or MUM-2B/CDDP cells upon JMJD2C silencing without CDDP treatment.

**Fig. S3.** The binding of p53 to ubiquitin as determined by co-IP.

**Fig. S4.** Representative images of Western blots. A, Representative images of Western blots for Fig. 2C. B, Representative images of Western blots for Fig. 3C. C, Representative images of Western blots for Fig. 3E. D, Representative images of Western blots for Fig. 3F. E, Representative images of Western blots for Fig. 4B. F, Representative images of Western blots for Fig. 4C. G, Representative images of Western blots for Fig. 4D. H, Representative images of Western blots for Fig. 4G. I, Representative images of Western blots for Fig. 4I. J, Representative images of Western blots for Fig. 5A. K, Representative images of Western blots for Fig. 6A.
