# Supplementary material for: JMJD2C mediates the MDM2/p53/IL5RA axis to promote CDDP resistance in uveal melanoma
Source: Cell Death Discov. 2022 Apr 25;8:227. doi: 10.1038/s41420-022-00949-y (PMC9039082; doi:10.1038/s41420-022-00949-y)
Supplement: Supplementary file 6 — Supplementary tables [file 41420_2022_949_MOESM6_ESM.docx]

**Table S1** Differential expression of genes enriched in tumor pathway

| symbol | logFC | AveExpr | P.Value | adj.P.Val |
| --- | --- | --- | --- | --- |
| PTK2 | -1.192171933 | 4.255151215 | 5.11E-07 | 0.000655402 |
| IL6ST | -1.09579625 | 9.81935665 | 1.47E-05 | 0.002683462 |
| ADCY9 | 1.443427875 | 6.97749335 | 2.10E-05 | 0.003301334 |
| GNA11 | -1.050147192 | 4.95581076 | 3.43E-05 | 0.004174695 |
| IL5RA | 2.102991442 | 4.75651029 | 4.38E-05 | 0.005049382 |
| RASSF1 | 1.426867425 | 8.00440853 | 7.68E-05 | 0.007148077 |
| LEF1 | -1.322501183 | 8.81491429 | 0.000519608 | 0.018535503 |
| DLL1 | -1.031846008 | 4.91248792 | 0.000593289 | 0.019620099 |
| GNG2 | 1.049339275 | 9.25683289 | 0.000713348 | 0.021489035 |
| CCNE1 | -1.1169407 | 6.12028718 | 0.000815813 | 0.023380828 |
| TCF7 | -1.5175165 | 8.79881325 | 0.000845602 | 0.023769897 |
| PTCH1 | 1.832104833 | 6.4334456 | 0.00092005 | 0.024664421 |
| PTGER2 | 1.957417775 | 8.096343515 | 0.001614216 | 0.033912815 |
| PRKCA | -1.140196875 | 8.9854013 | 0.001760848 | 0.035383042 |

**Table S2** The binding domain of TP53 in the promoter region of IL5RA

| **TF** | **Pattern name** | **Sequence name** | **Start** | **Stop** | **Strand** | **Score** | **P value** | **Q value** | **Matched motif** |
| --- | --- | --- | --- | --- | --- | --- | --- | --- | --- |
| TP53 | m-dataset-3640-1 | NG_029547.1:3001-5001 | 691 | 706 | + | 10.4545 | 0.0000507 | 0.102 | AGACAAGCCTGGCCAA |
| TP53 | m-dataset-3650-1 | NG_029547.1:3001-5001 | 390 | 407 | + | -6.38889 | 0.000088 | 0.082 | CAGAAACATAGAGACAGT |
| TP53 | m-dataset-3645-3 | NG_029547.1:3001-5001 | 298 | 313 | + | 14.4458 | 0.00000615 | 0.0241 | ATTCCATCTCAAAACA |
| TP53 | m-dataset-3629-4 | NG_029547.1:3001-5001 | 416 | 431 | + | 12.4342 | 0.0000189 | 0.074 | ATGAGCATGTGTTTGC |
| TP53 | m-dataset-3651-4 | NG_029547.1:3001-5001 | 818 | 829 | + | 12.1938 | 0.000019 | 0.0363 | GGGAGGCAGAGG |
| TP53 | m-dataset-3650-1 | NG_029547.1:3001-5001 | 388 | 405 | + | 0.319444 | 0.0000205 | 0.05 | TACAGAAACATAGAGACA |
| TP53 | m-dataset-3650-4 | NG_029547.1:3001-5001 | 390 | 405 | + | 12.2353 | 0.000024 | 0.0309 | CAGAAACATAGAGACA |
| TP53 | m-dataset-3650-4 | NG_029547.1:3001-5001 | 388 | 403 | + | 12.2059 | 0.0000246 | 0.0309 | TACAGAAACATAGAGA |
| TP53 | m-dataset-3627-2 | NG_029547.1:3001-5001 | 1594 | 1615 | + | 10.6154 | 0.0000277 | 0.105 | AACAGAAATGGAACAAAGGACA |
| TP53 | m-dataset-3650-2 | NG_029547.1:3001-5001 | 1959 | 1978 | + | 2.77465 | 0.0000282 | 0.0588 | GAGACAGAGGCACAGTAACT |
| TP53 | m-dataset-3651-2 | NG_029547.1:3001-5001 | 386 | 404 | + | 4.50704 | 0.0000299 | 0.0377 | TATACAGAAACATAGAGAC |
| TP53 | m-dataset-3635-2 | NG_029547.1:3001-5001 | 342 | 353 | + | 12.3462 | 0.0000334 | 0.127 | CAGAAAAAAAAA |

**Table S3** Primer sequences for RT-qPCR

| Gene | Sequence |
| --- | --- |
| JMJD2C | F: 5’-AGGCCTAAGGCTGATGAGGA-3’ |
|  | R: 5’-TTGGCCATGAAAGCTCGGAT-3’ |
| MDM2 | F: 5’-TGTAAGTGAACATTCAGGTG-3’ |
|  | R: 5’-TTCCAATAGTCAGCTAAGGA-3’ |
| p53 | F: 5’-TGCGTGTGGAGTATTTGGATG-3’ |
|  | R: 5’-TGGTACAGTCAGAGCCAACCTC-3’ |
| IL5RA | F: 5’-TGACTGGCTTGCGGTGCTTGTT-3’ |
|  | R: 5’-CTGCTGTGACATTCAGTGGAGG-3’ |
| YY1 | F: 5’-AACAGGCATCCCGAGTTCAG-3’ |
|  | R: 5’-GGGGGCTAAAATCACAGCCT-3’ |
| MEF2A | F: 5’-ACGTCGCCTCCTTGAAAGTT-3’ |
|  | R: 5’-TCCGCCCCATTTTCAGTCAA-3’ |
| STAT3 | F: 5’-CATCCTGAAGCTGACCCAGG-3’ |
|  | R: 5’-TCCTCACATGGGGGAGGTAG-3’ |
| NR3C1 | F: 5’-GTTGCCAAGCGTCACCAAC-3’ |
|  | R: 5’-GTCTTCGCTGCTTGGAGTCT-3’ |
| GAPDH | F: 5’-ATGGCACCGTCAAGGCTGAG-3’ |
|  | R: 5’-GCAGTGATGGCATGGACTGT-3’ |

Note: F, forward; R, reverse; JMJD2C, jumonji domain containing 2C; MDM2, mouse double minute-2 homolog; IL5RA, interleukin 5 receptor subunit alpha; GAPDH, glyceraldehyde-3-phosphate dehydrogenase; RT-qPCR, reverse transcription-quantitative polymerase chain reaction
