# Supplementary material for: JMJD2C mediates the MDM2/p53/IL5RA axis to promote CDDP resistance in uveal melanoma
Source: Cell Death Discov. 2022 Apr 25;8:227. doi: 10.1038/s41420-022-00949-y (PMC9039082; doi:10.1038/s41420-022-00949-y)
Supplement: Supplementary file 7 — Original Data File [file 41420_2022_949_MOESM7_ESM.docx]

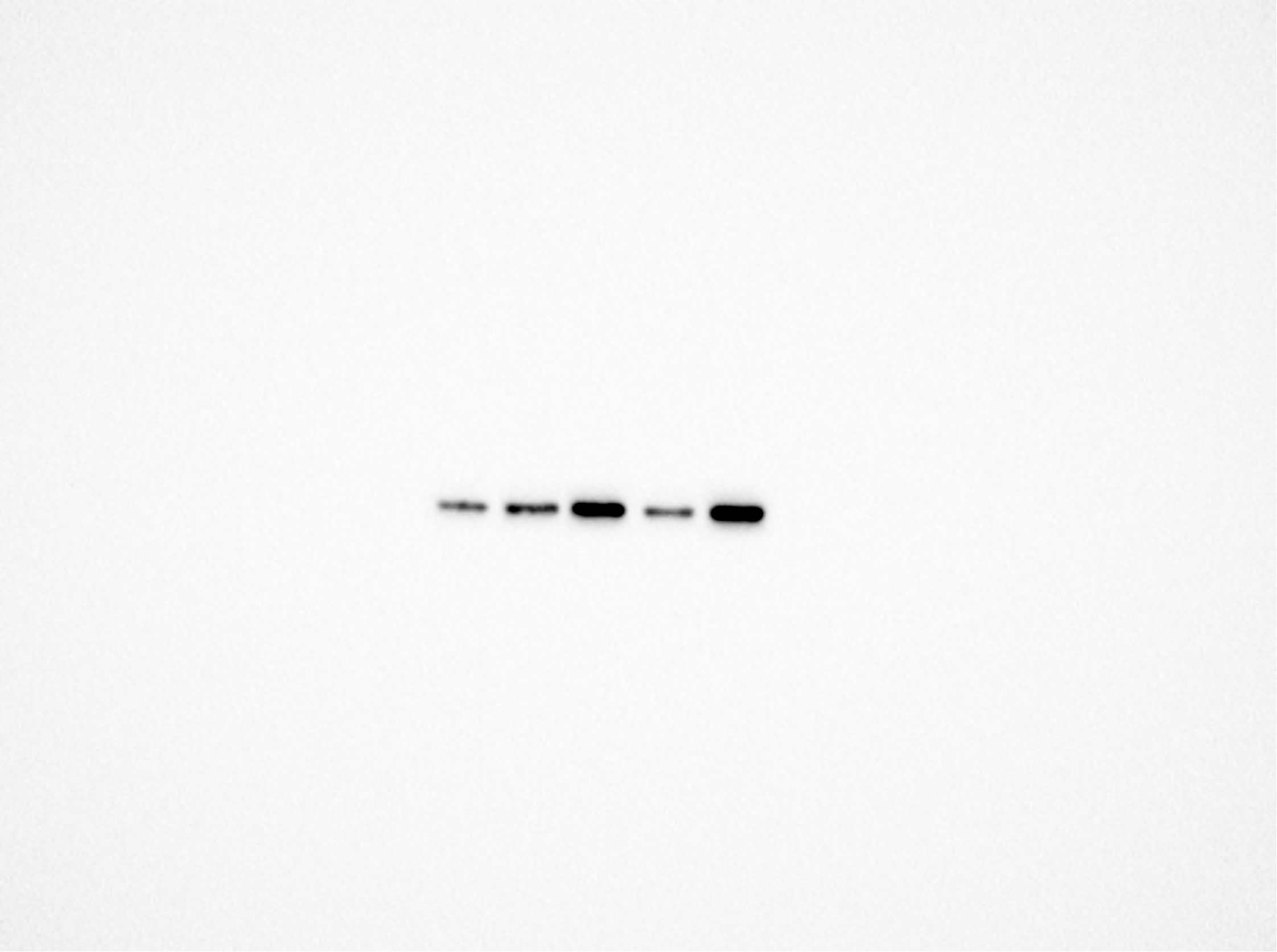


**Figure S3 MDM2**


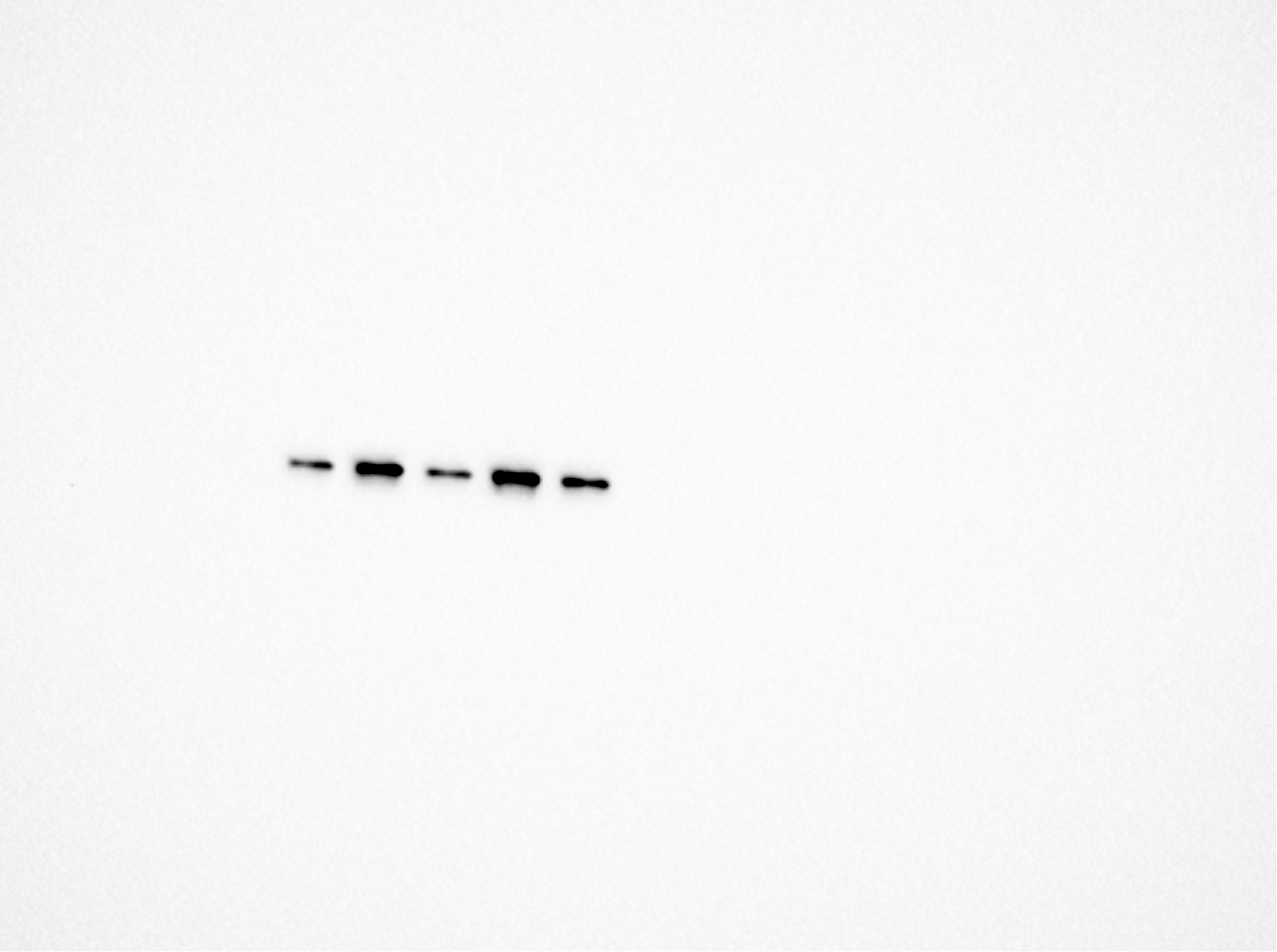


**Figure S3 p53**


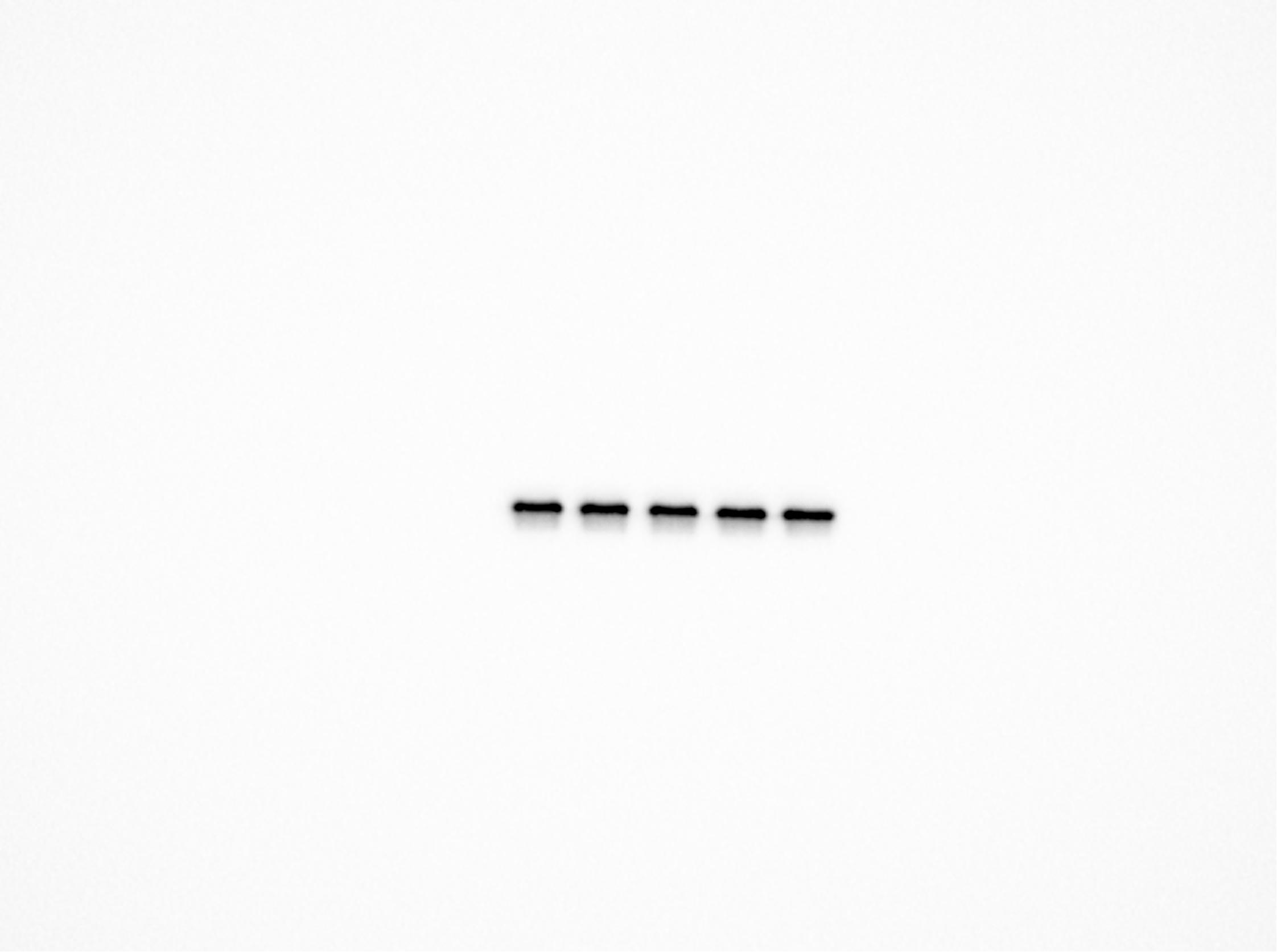


**Figure S3 GAPDH**


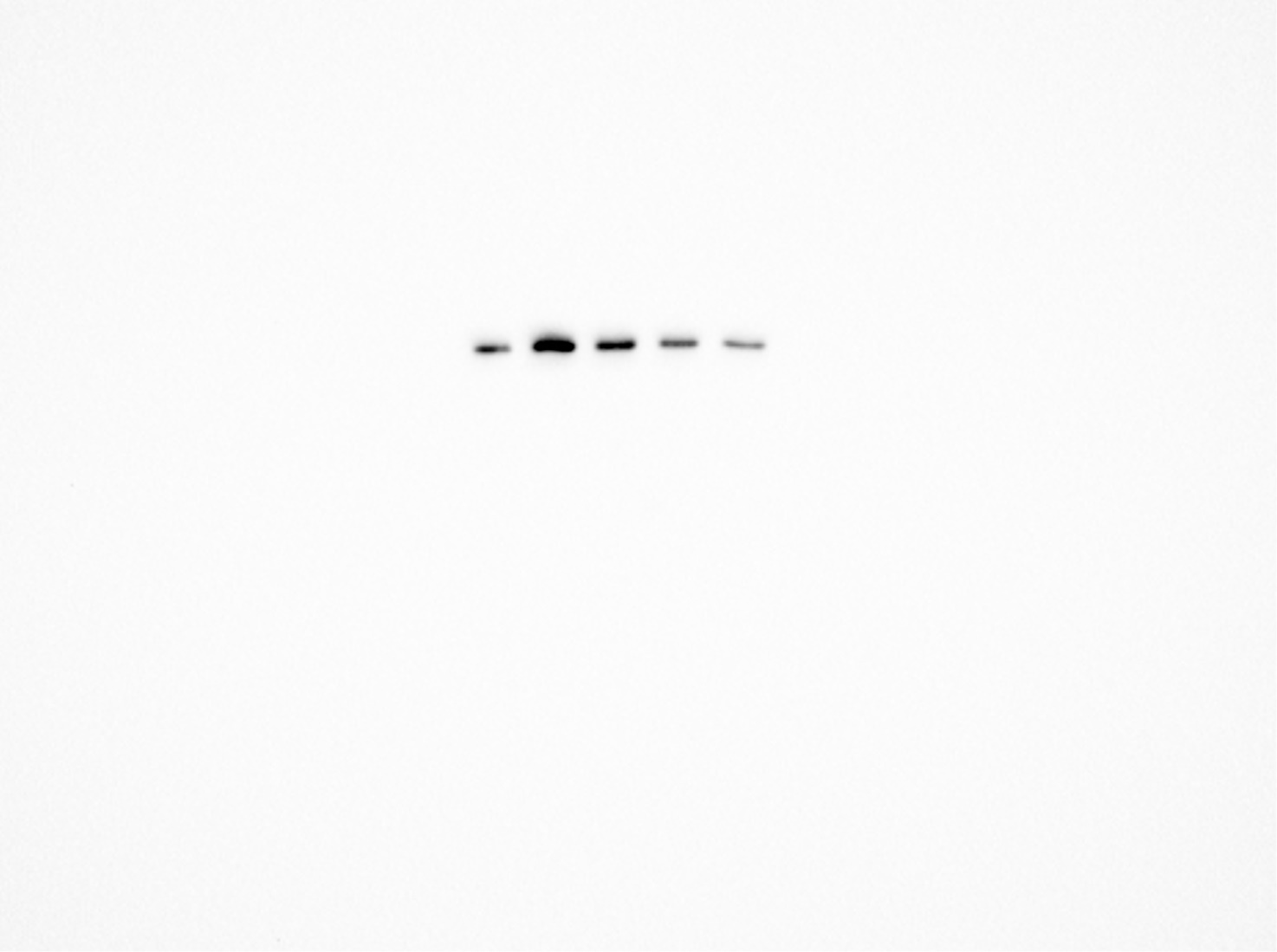


**Figure S4A 1-JMJD2C**


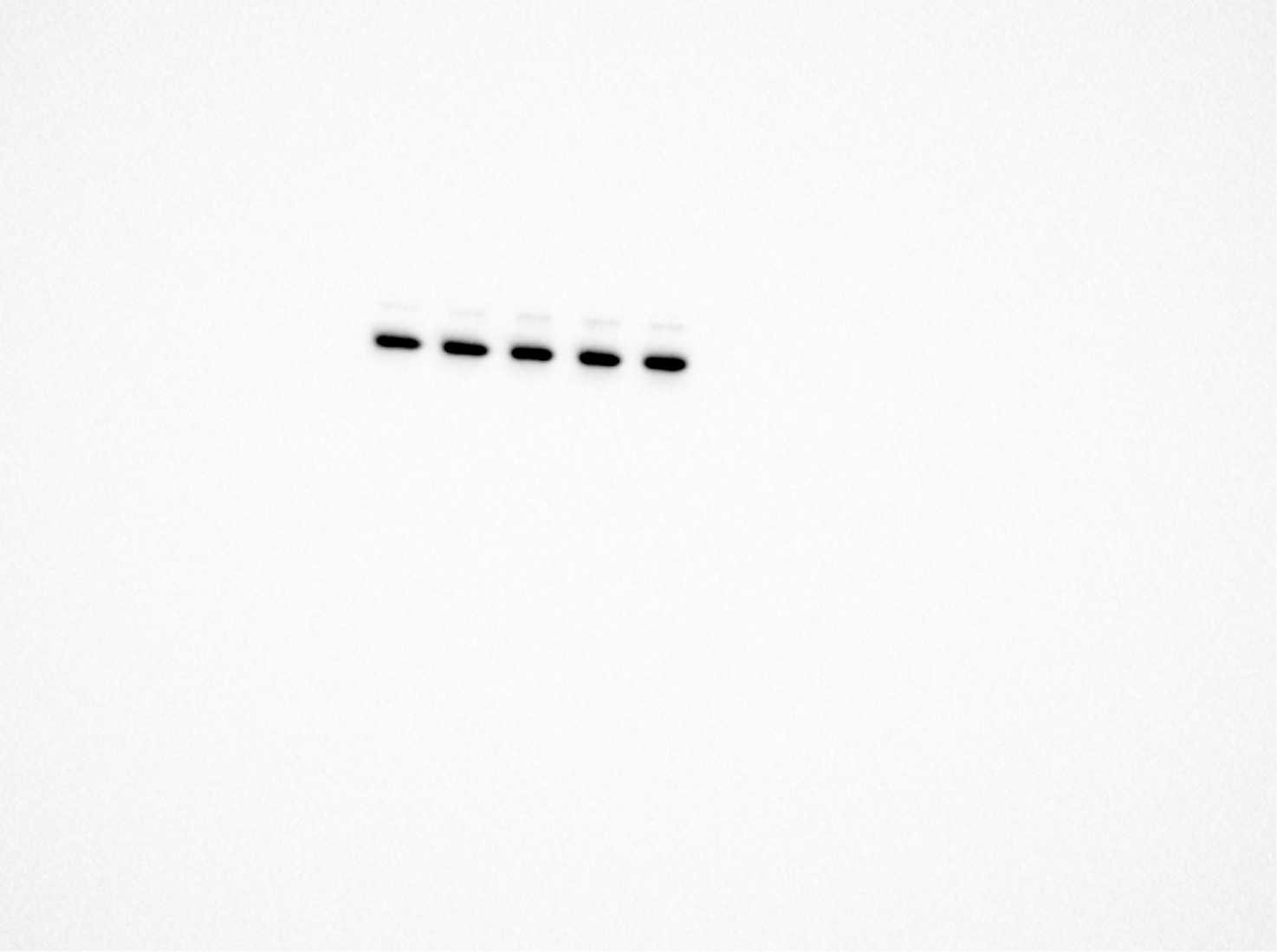


**Figure S4A 2-GAPDH(1)**


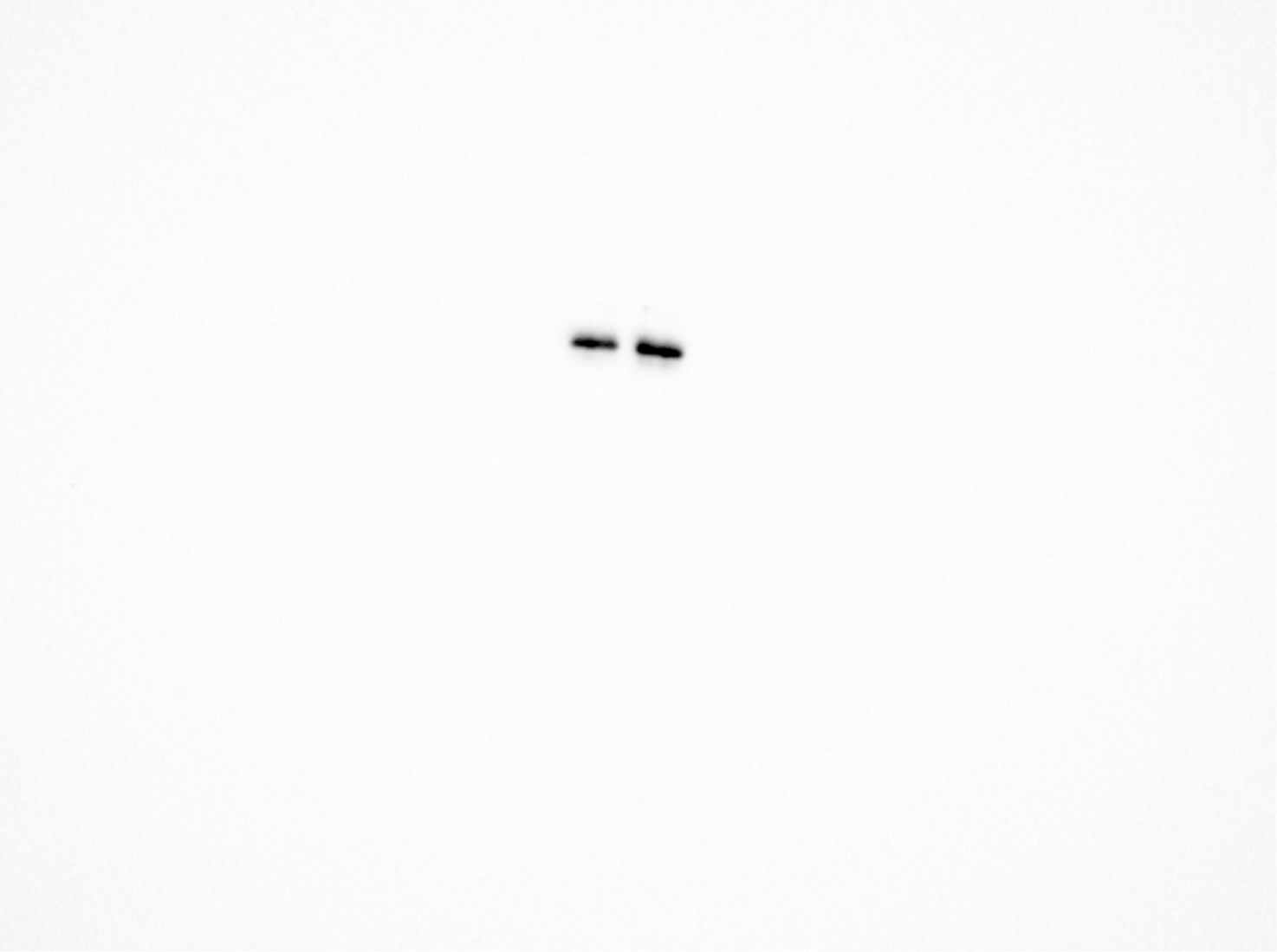


**Figure S4B 1-MDM2**


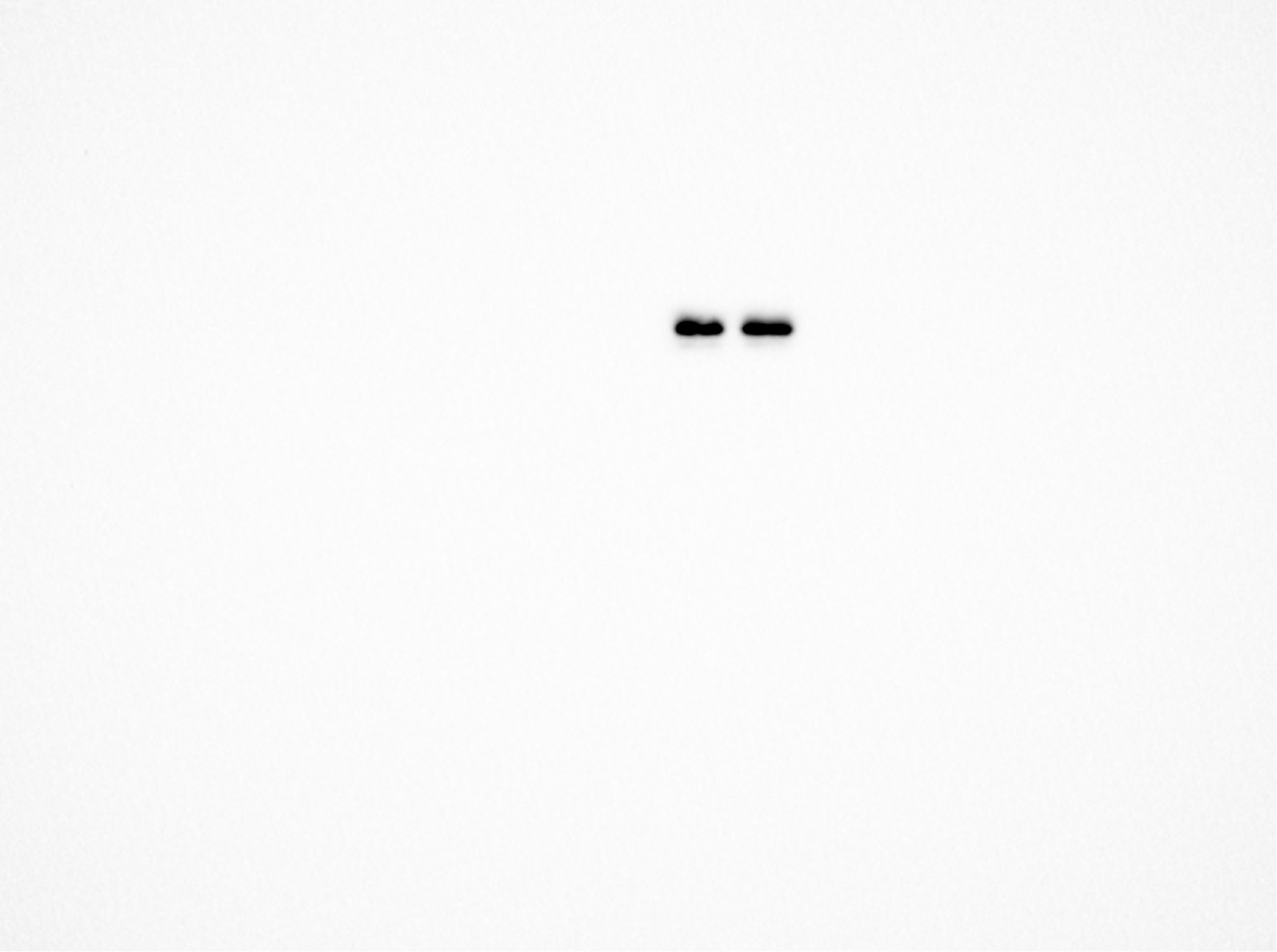


**Figure S4B 2-GAPDH**


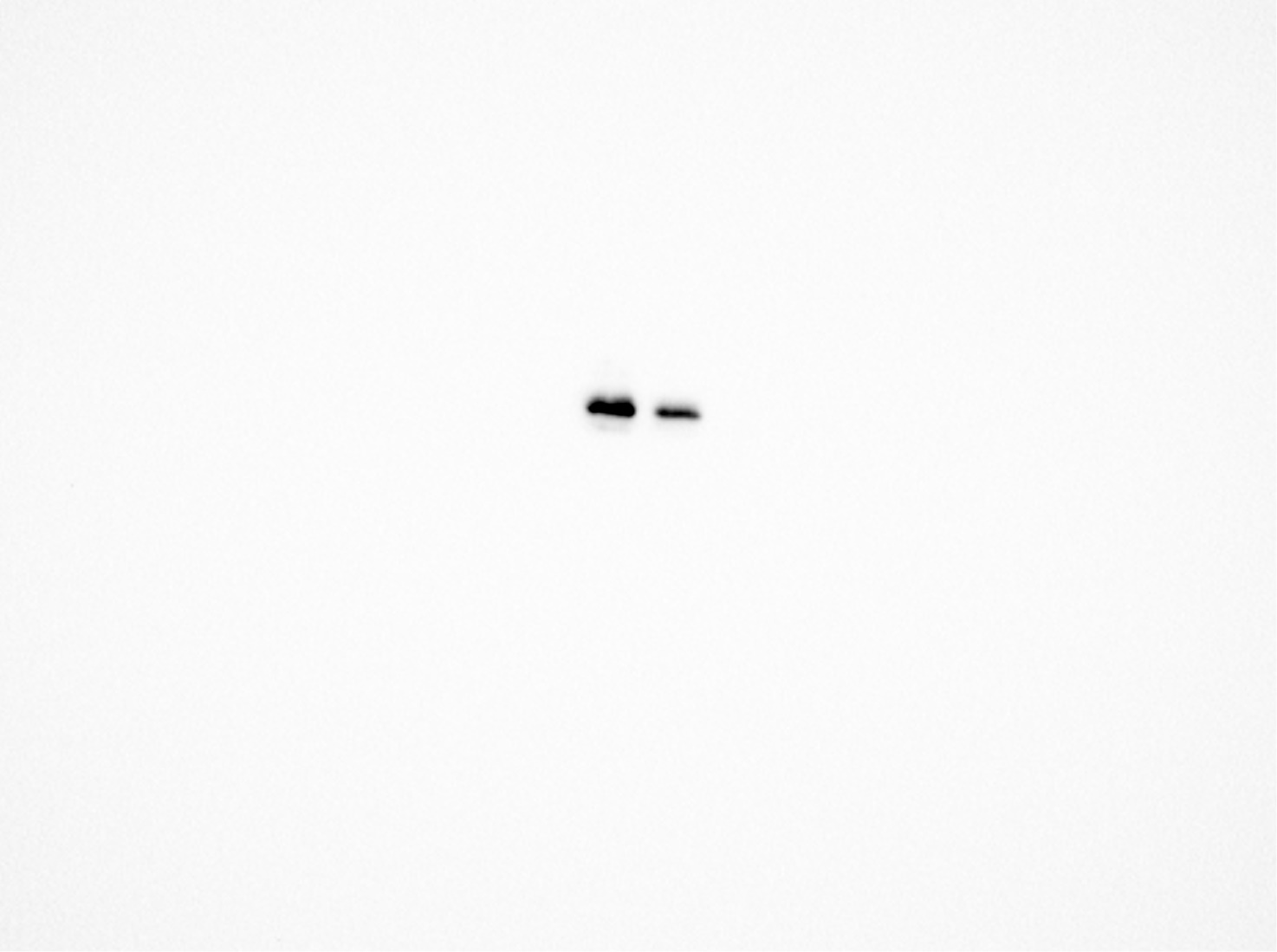


**Figure S4B 3-MDM2**


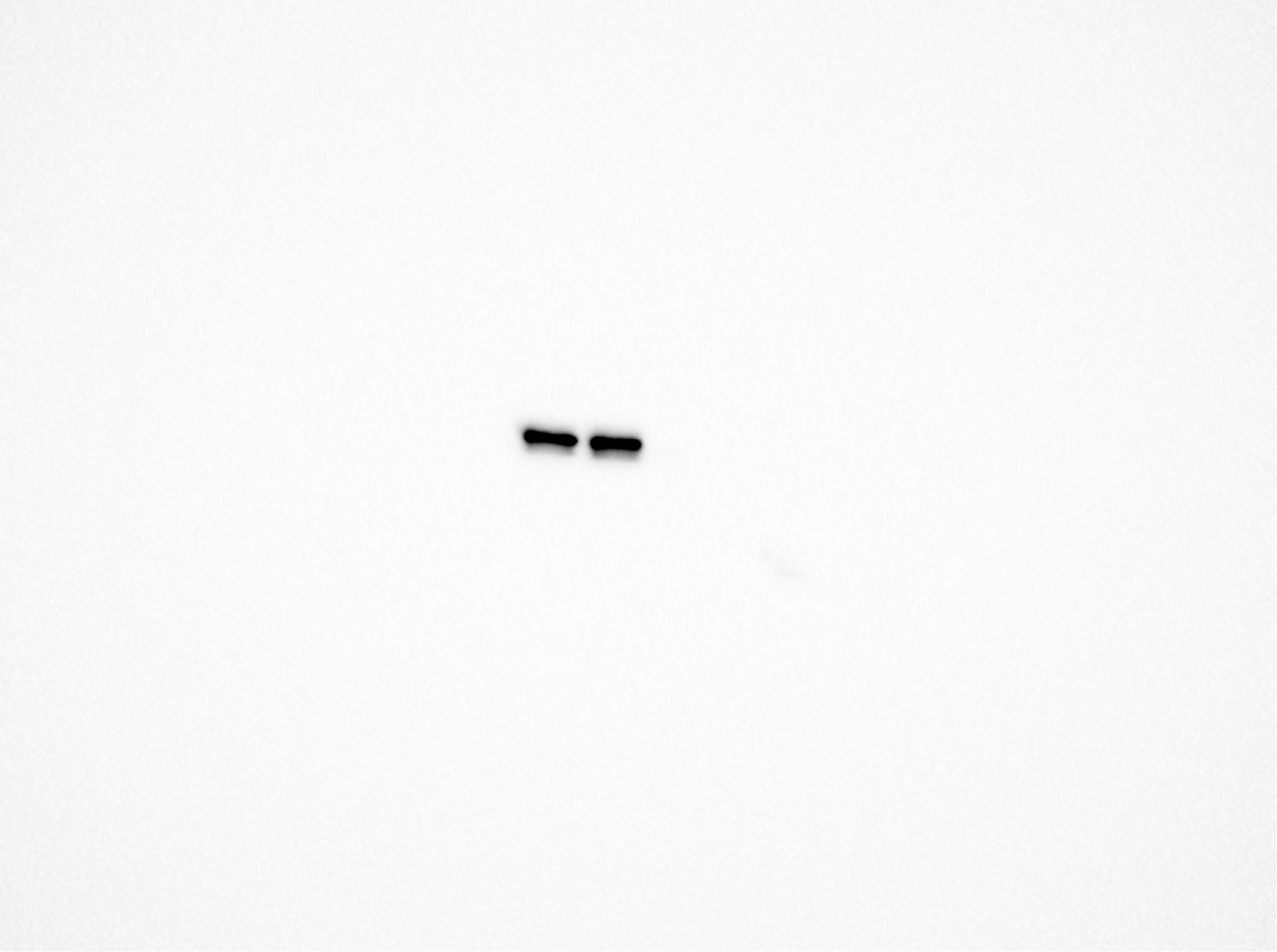


**Figure S4B 4-GAPDH**


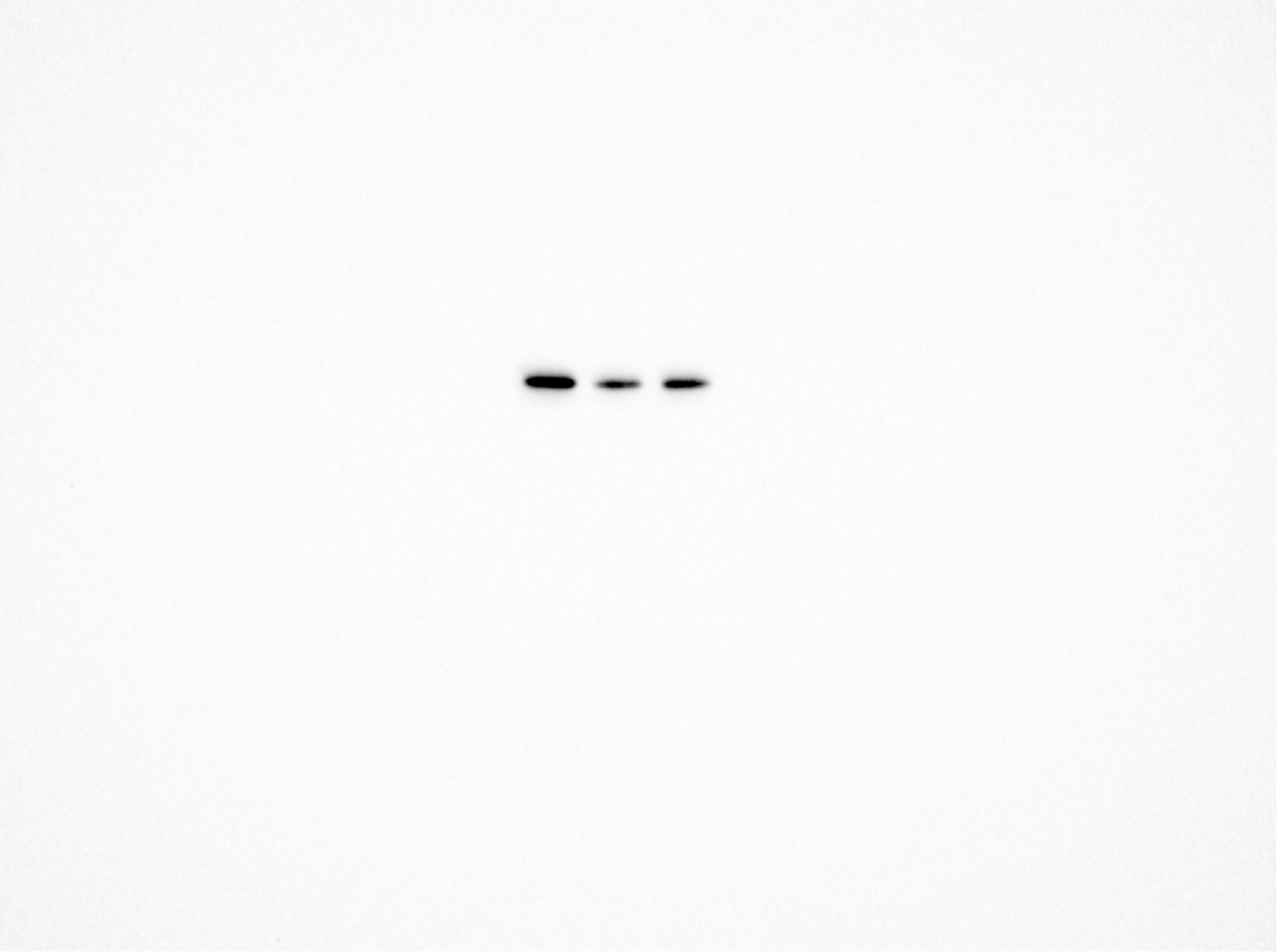


**Figure S4C 1-MDM2**


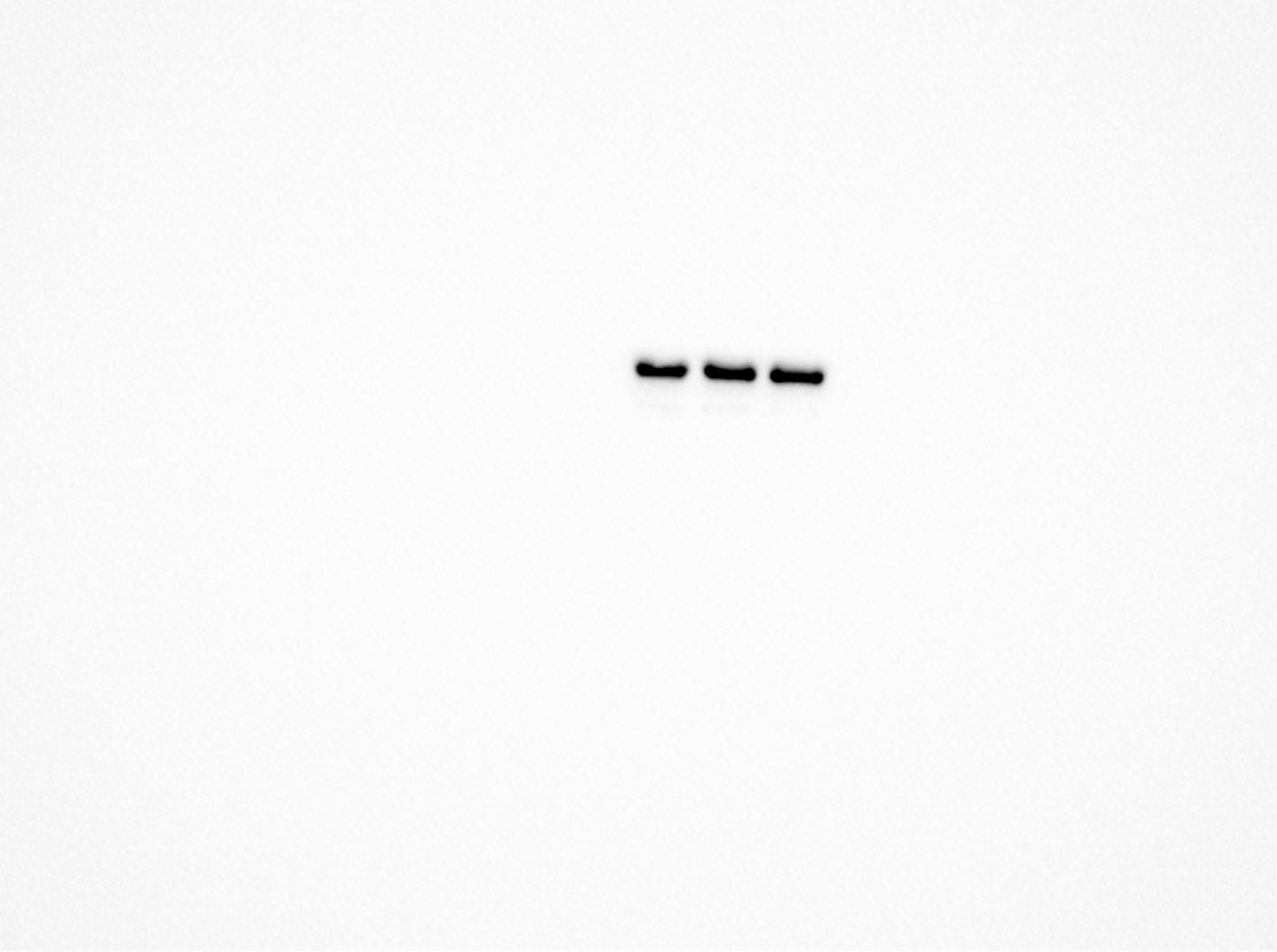


**Figure S4C 2-GAPDH**


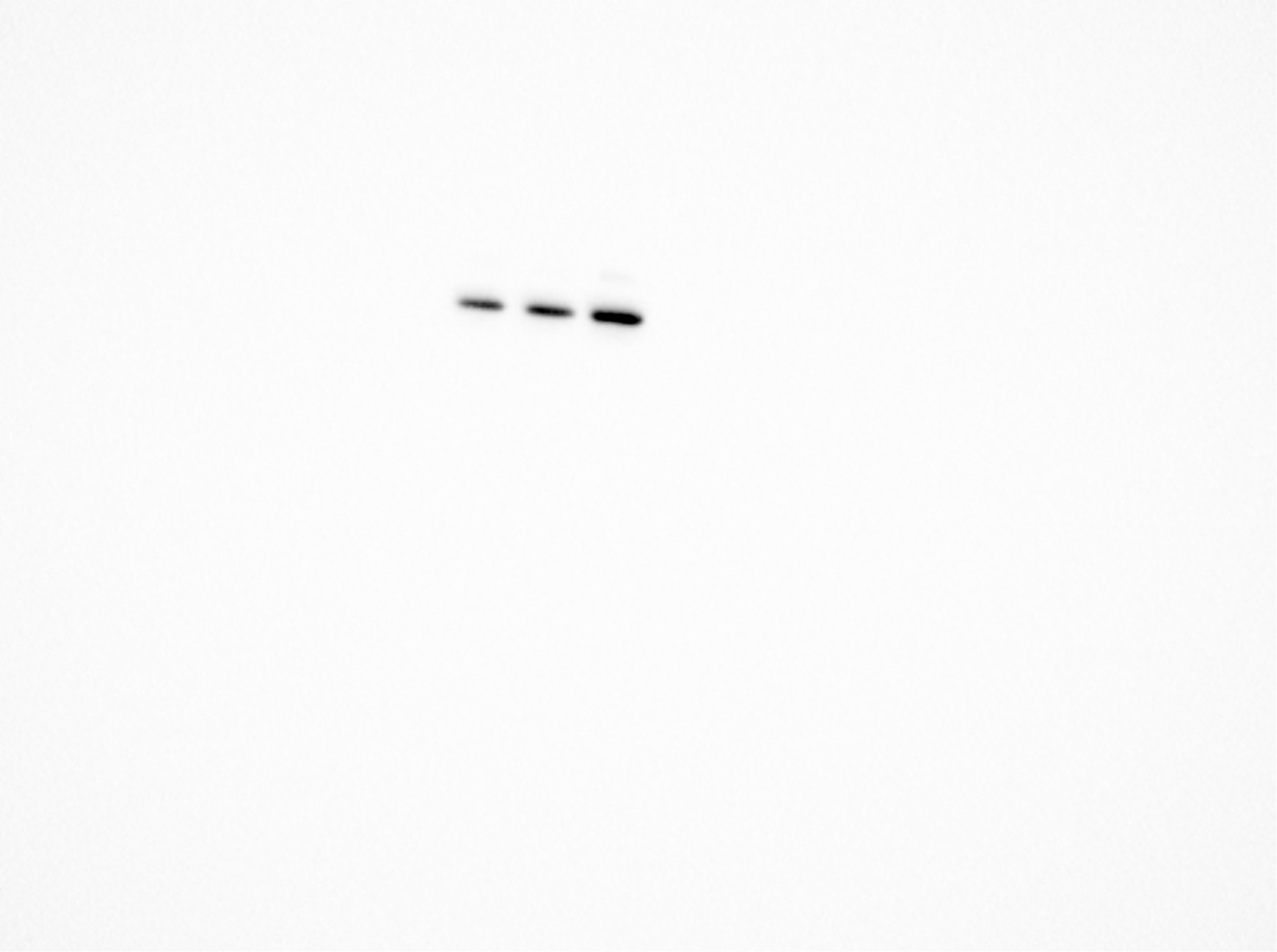


**Figure S4D 1-JMJD2C**


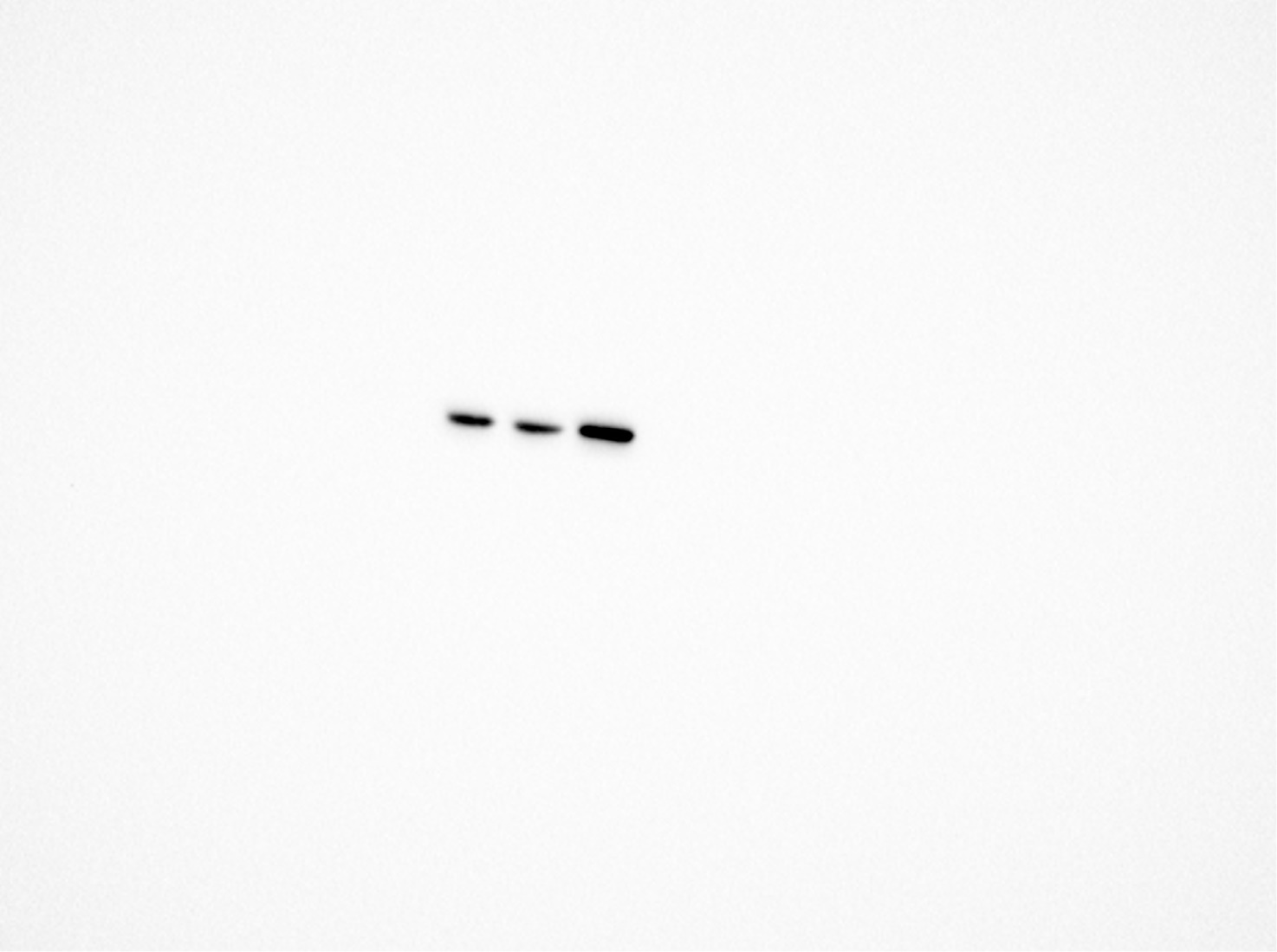


**Figure S4D 2-MDM2**


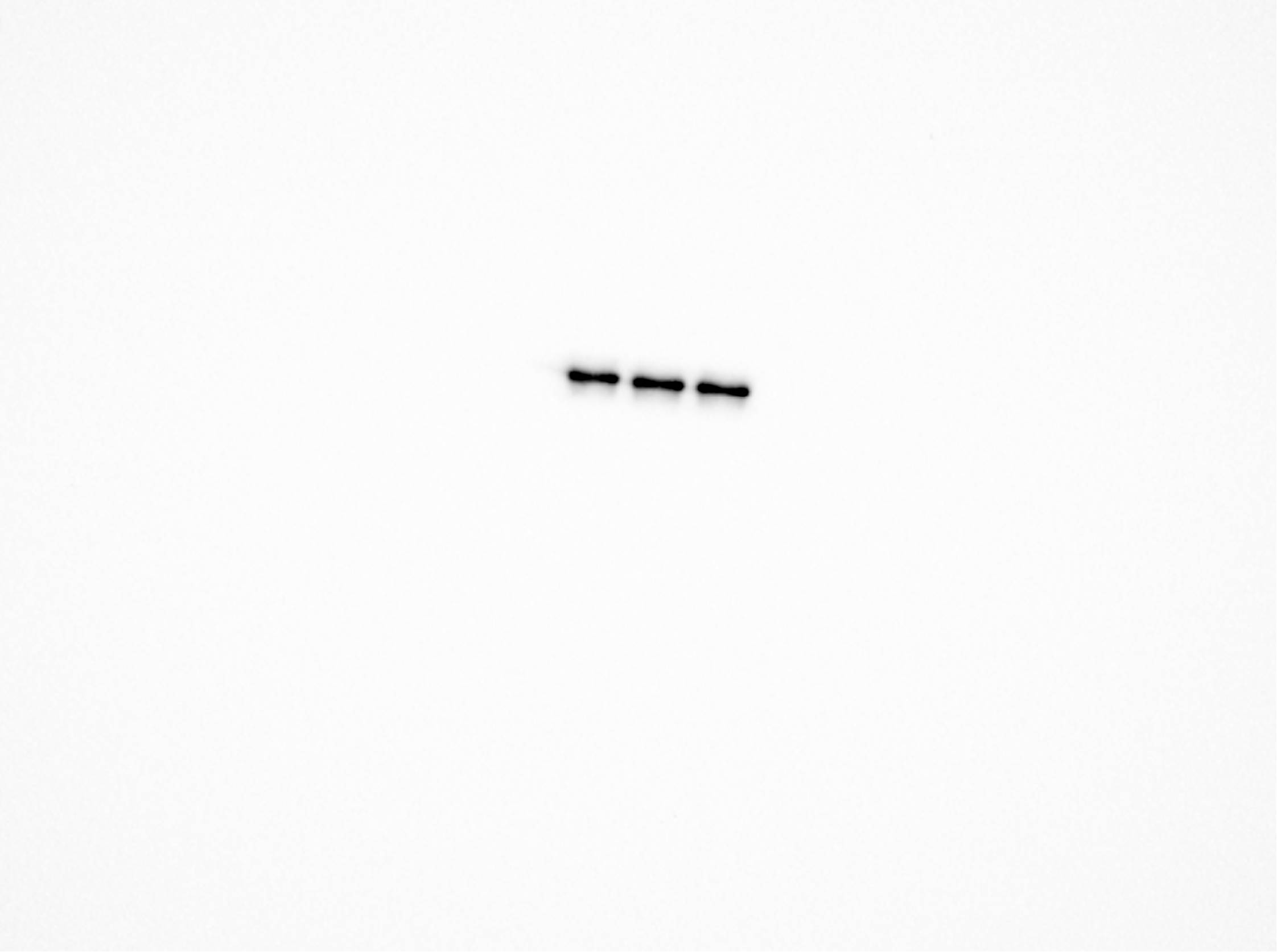


**Figure S4D 3-GAPDH**


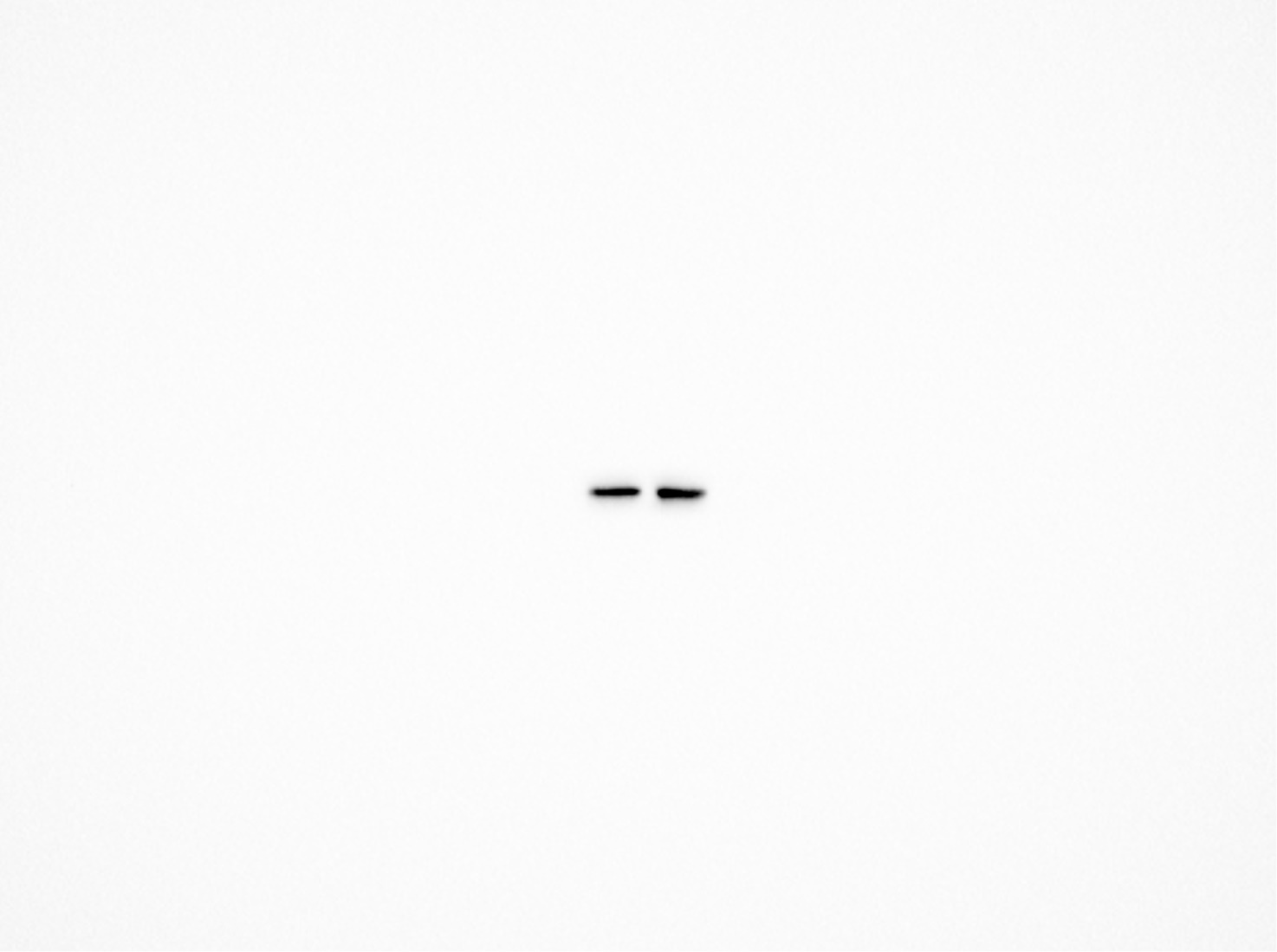


**Figure S4E 1-p53**


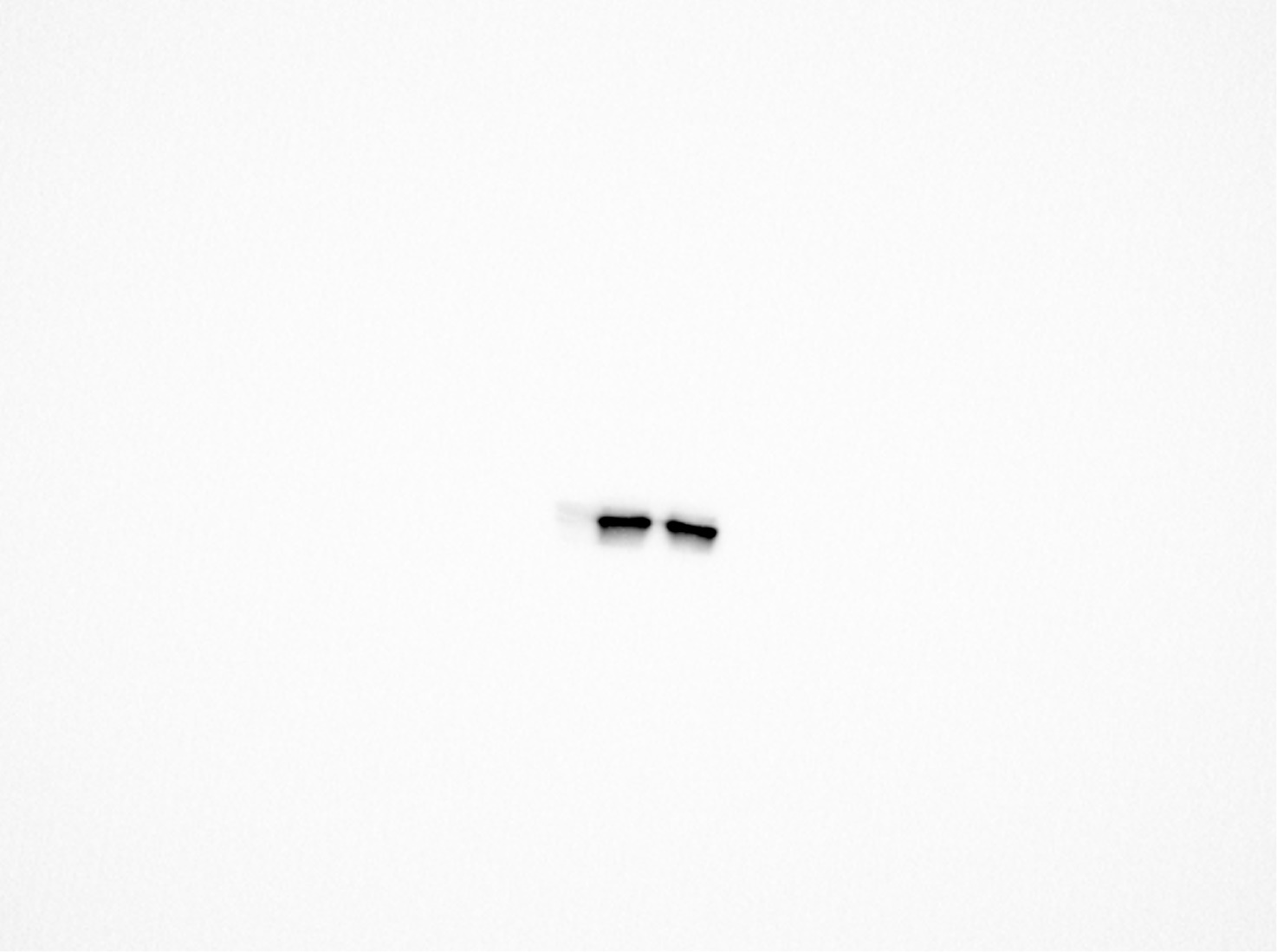


**Figure S4E 2-GAPDH**


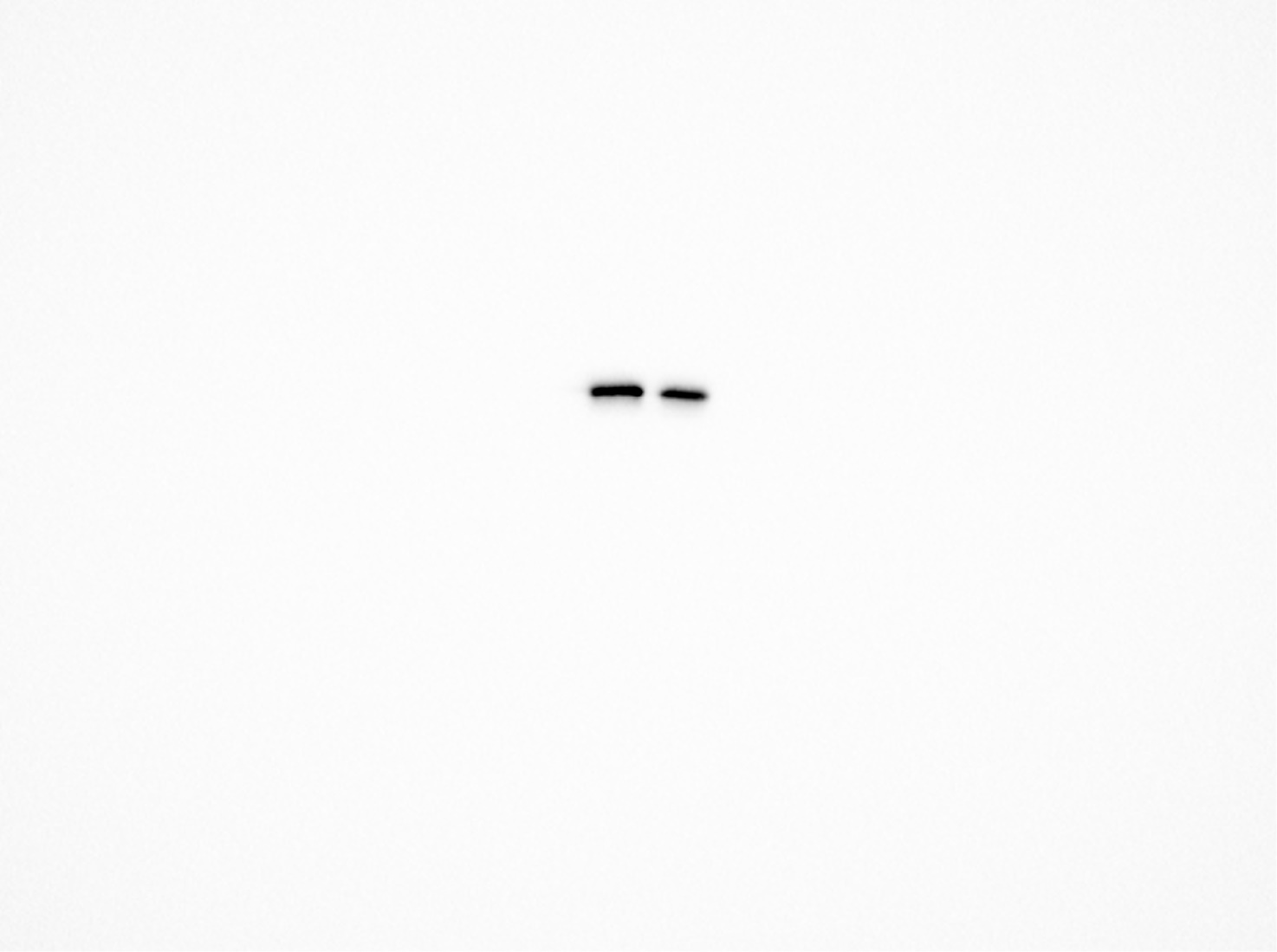


**Figure S4E 3-p53**


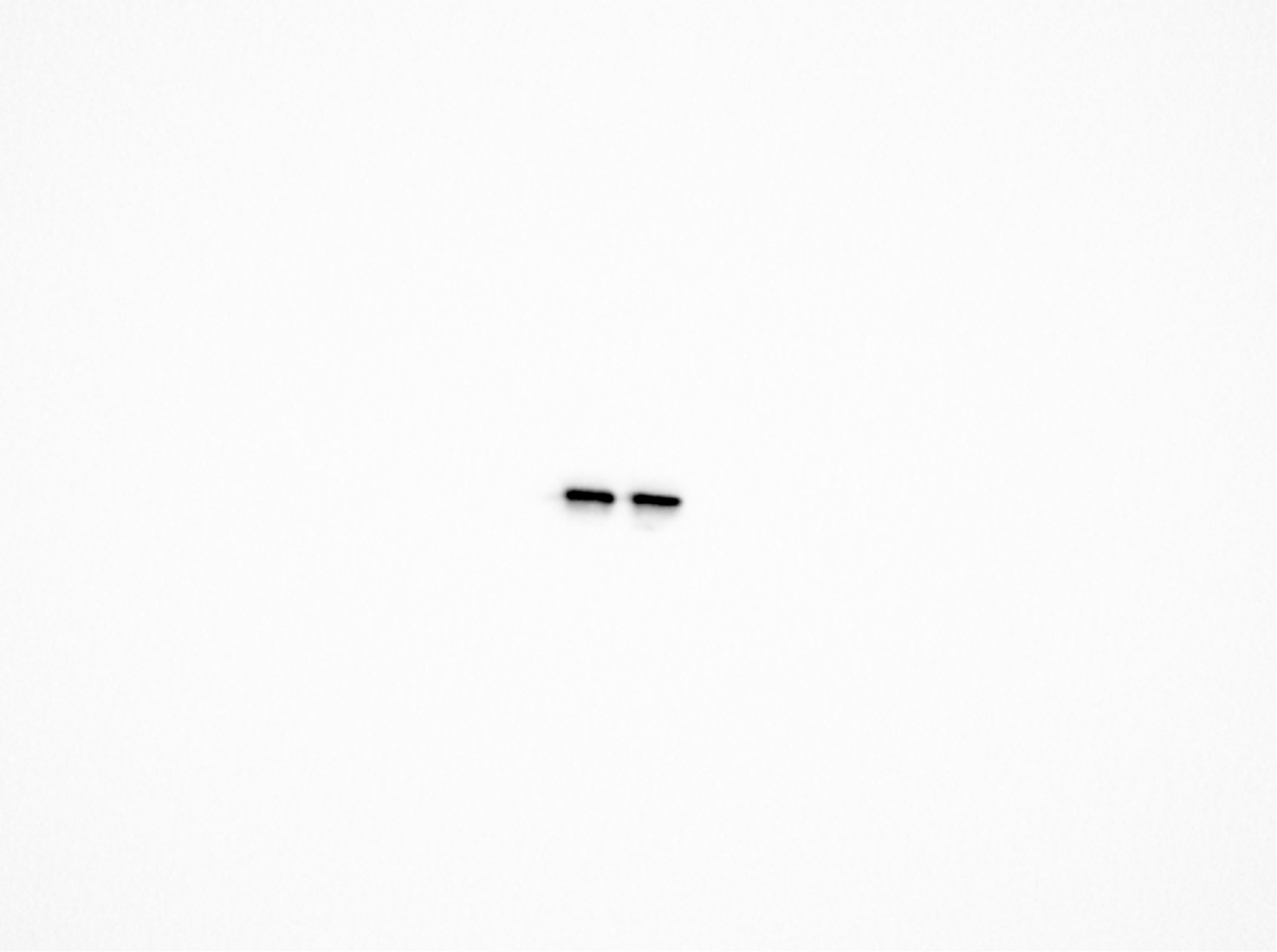


**Figure S4E 4-GAPDH**


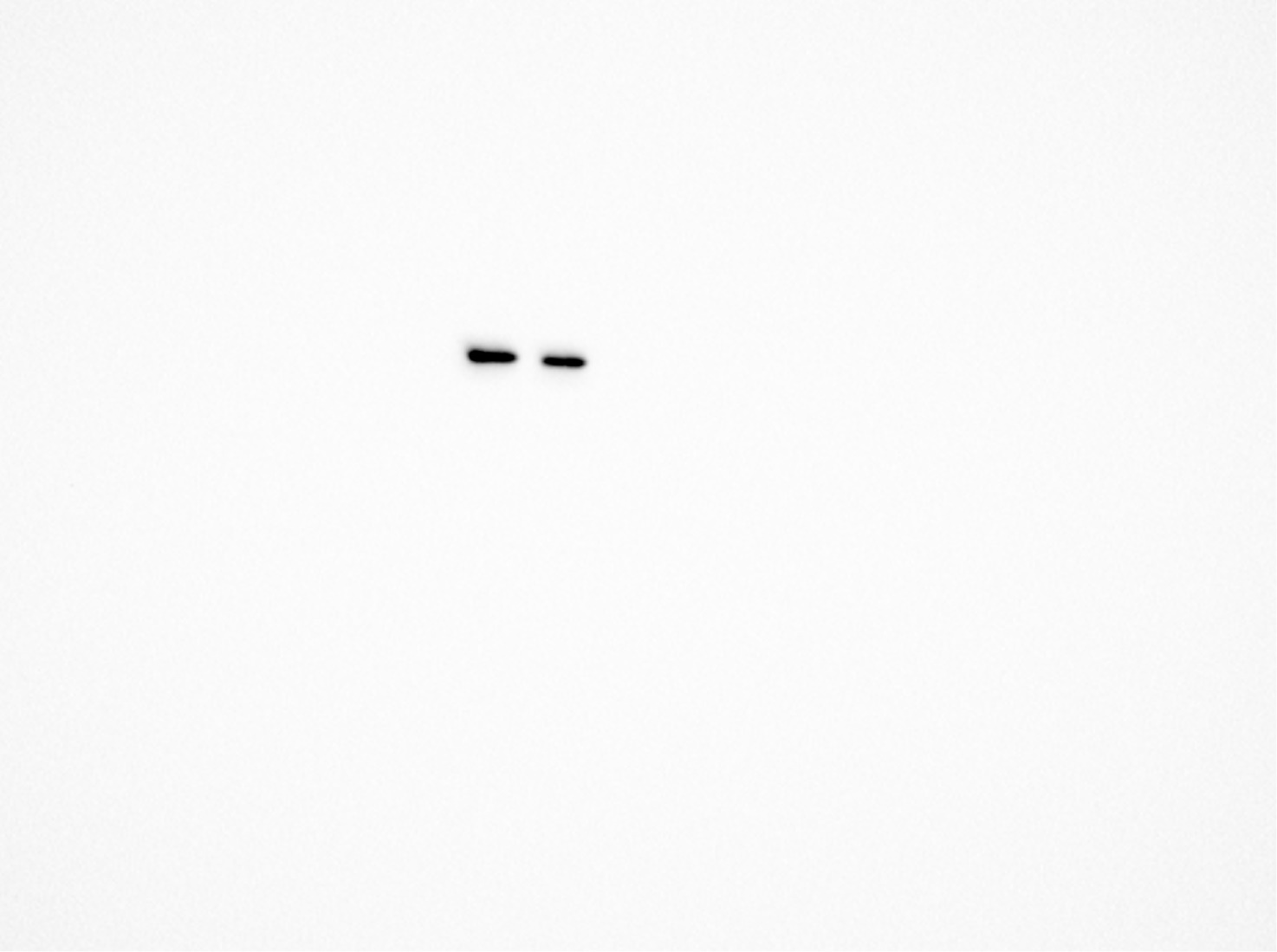


**Figure S4F 1-P53**


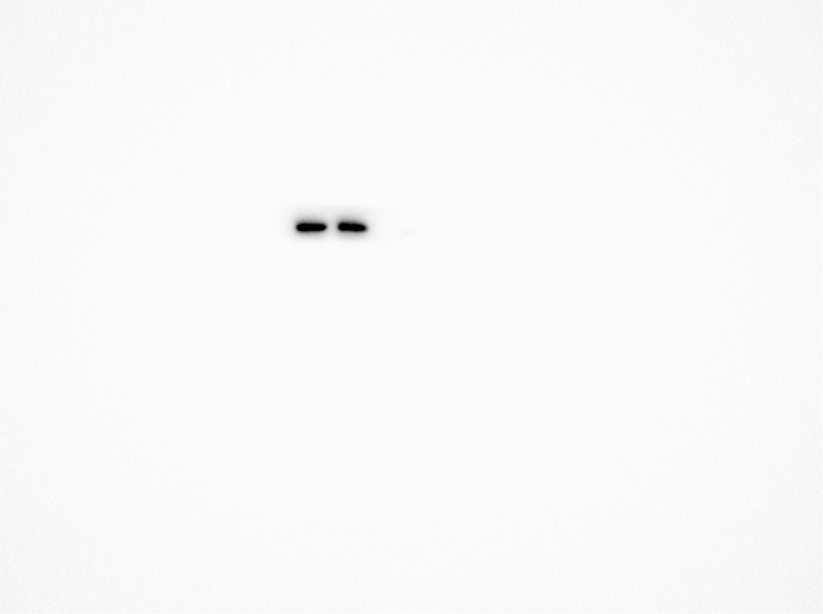


**Figure S4F 2-GAPDH**


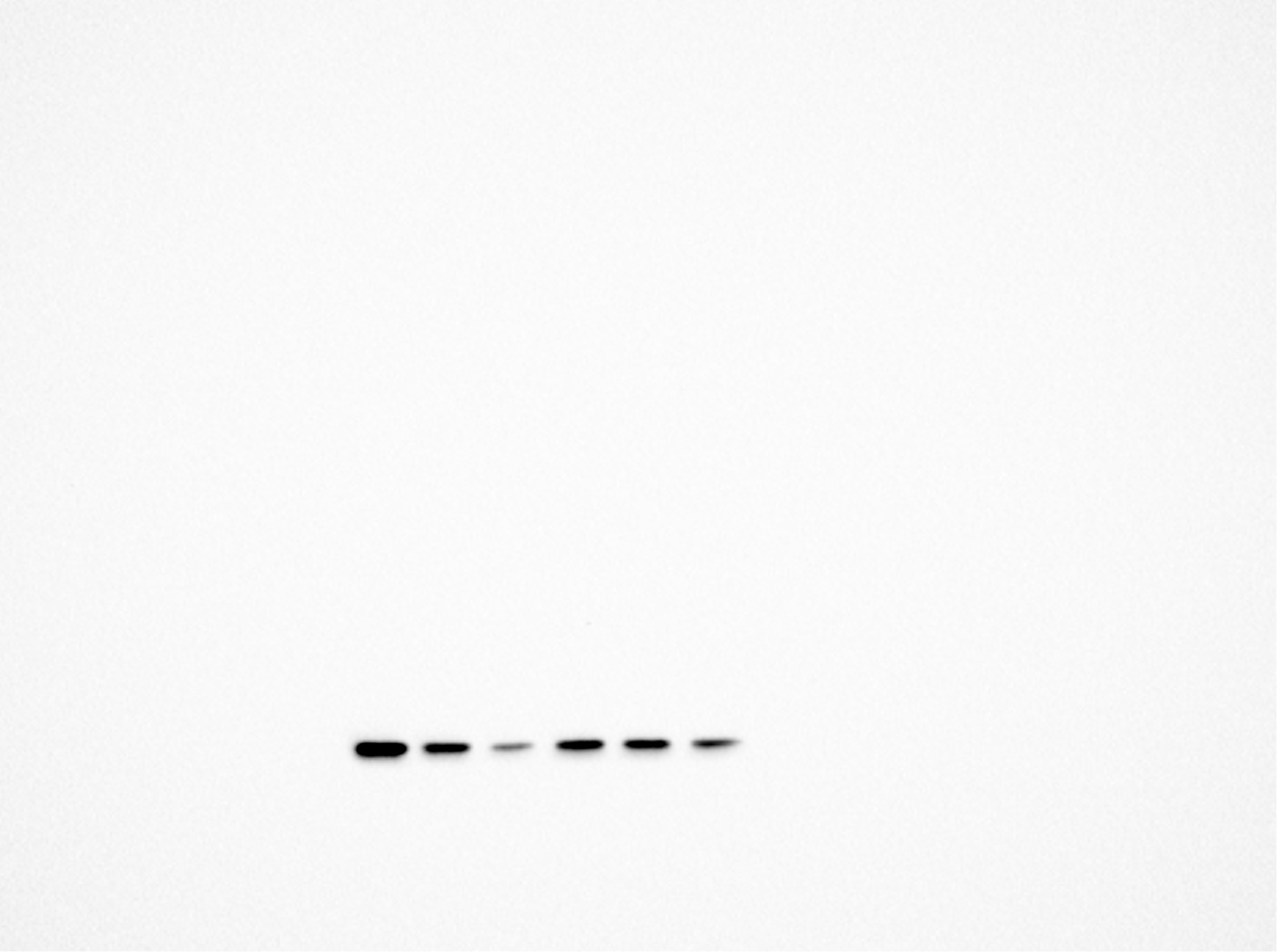


**Figure S4G 1-p53**


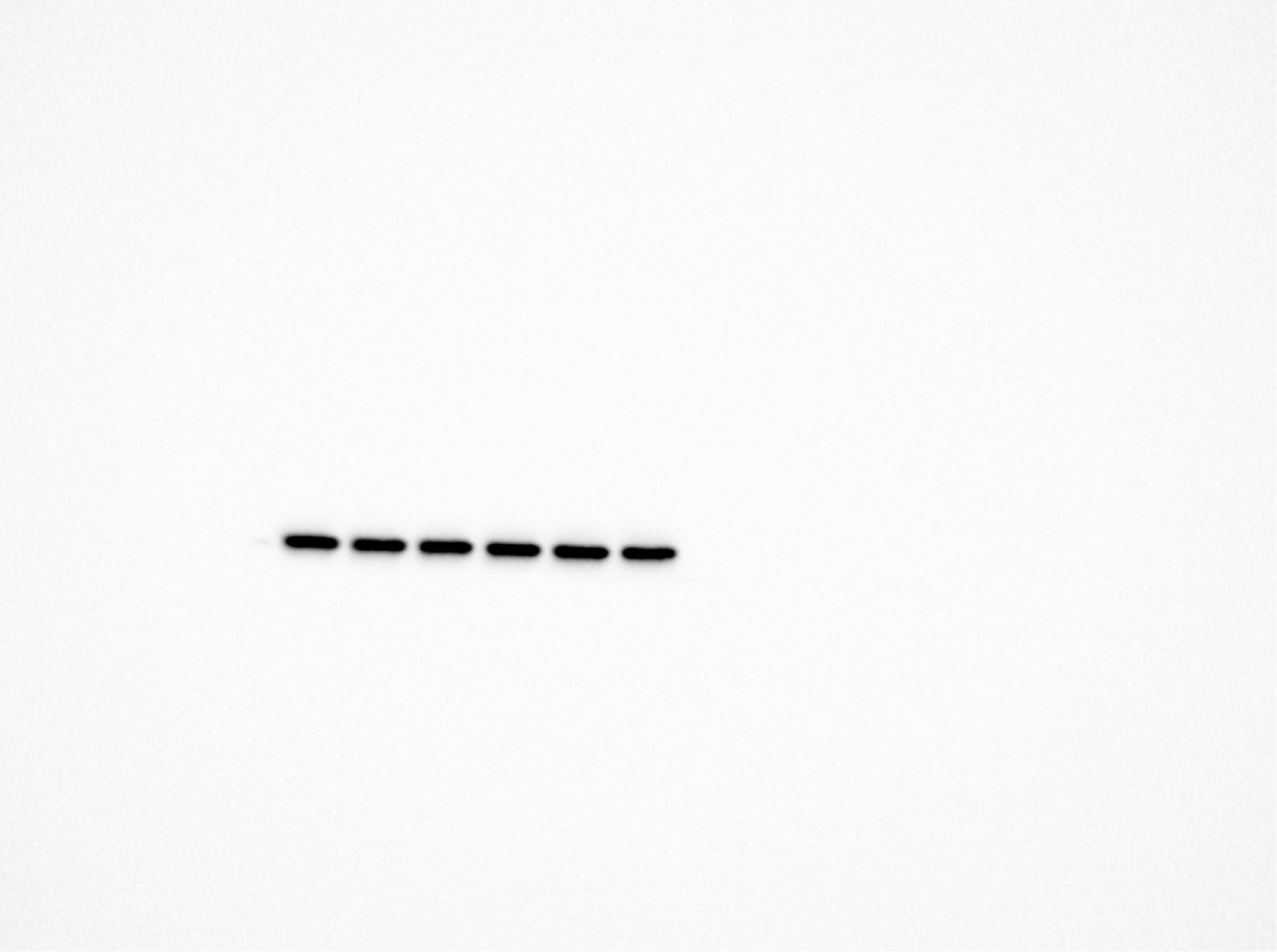


**Figure S4G 2-GAPDH**


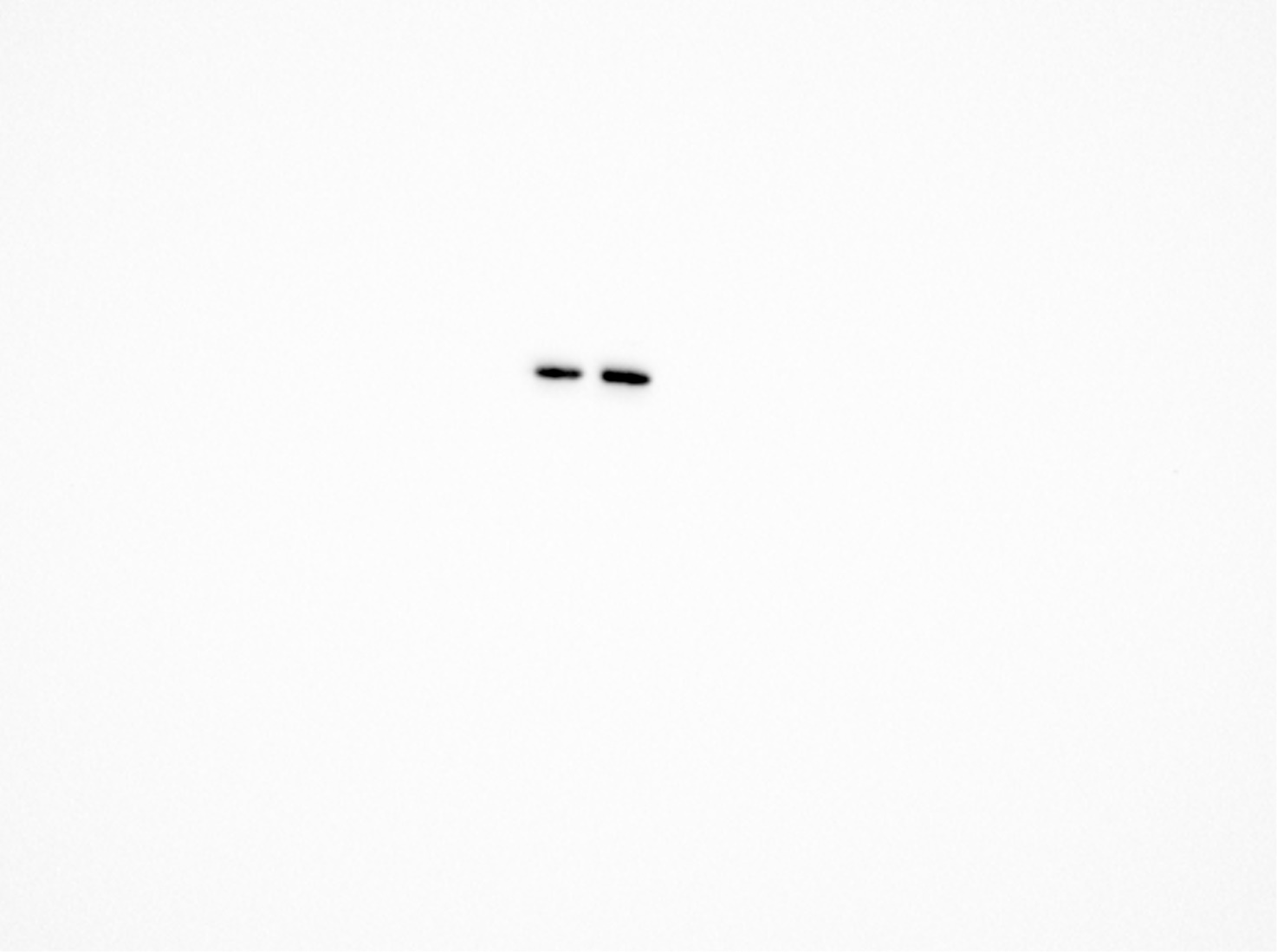


**Figure S4H 1-IL5RA**


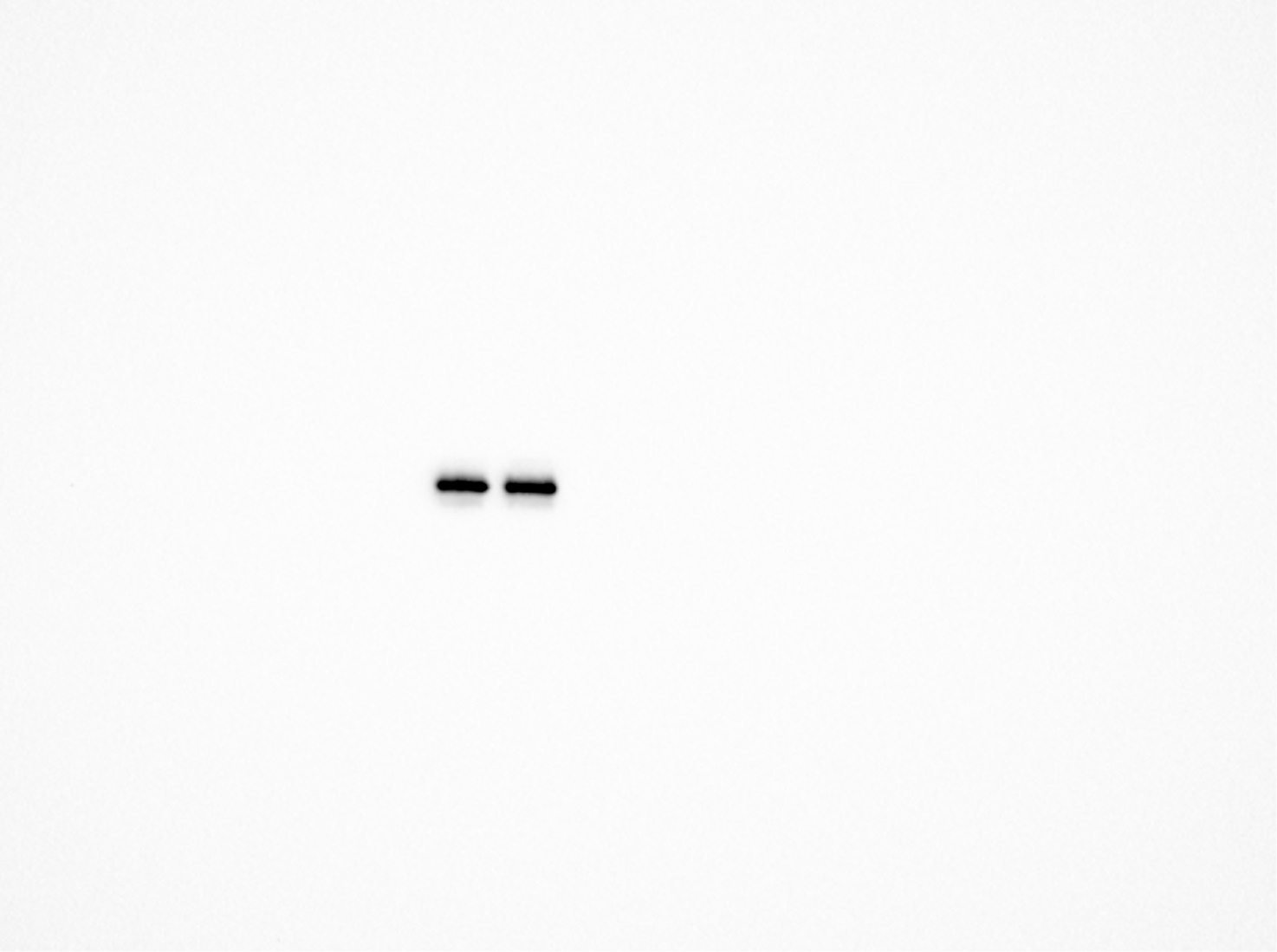


**Figure S4H 2-GAPDH**


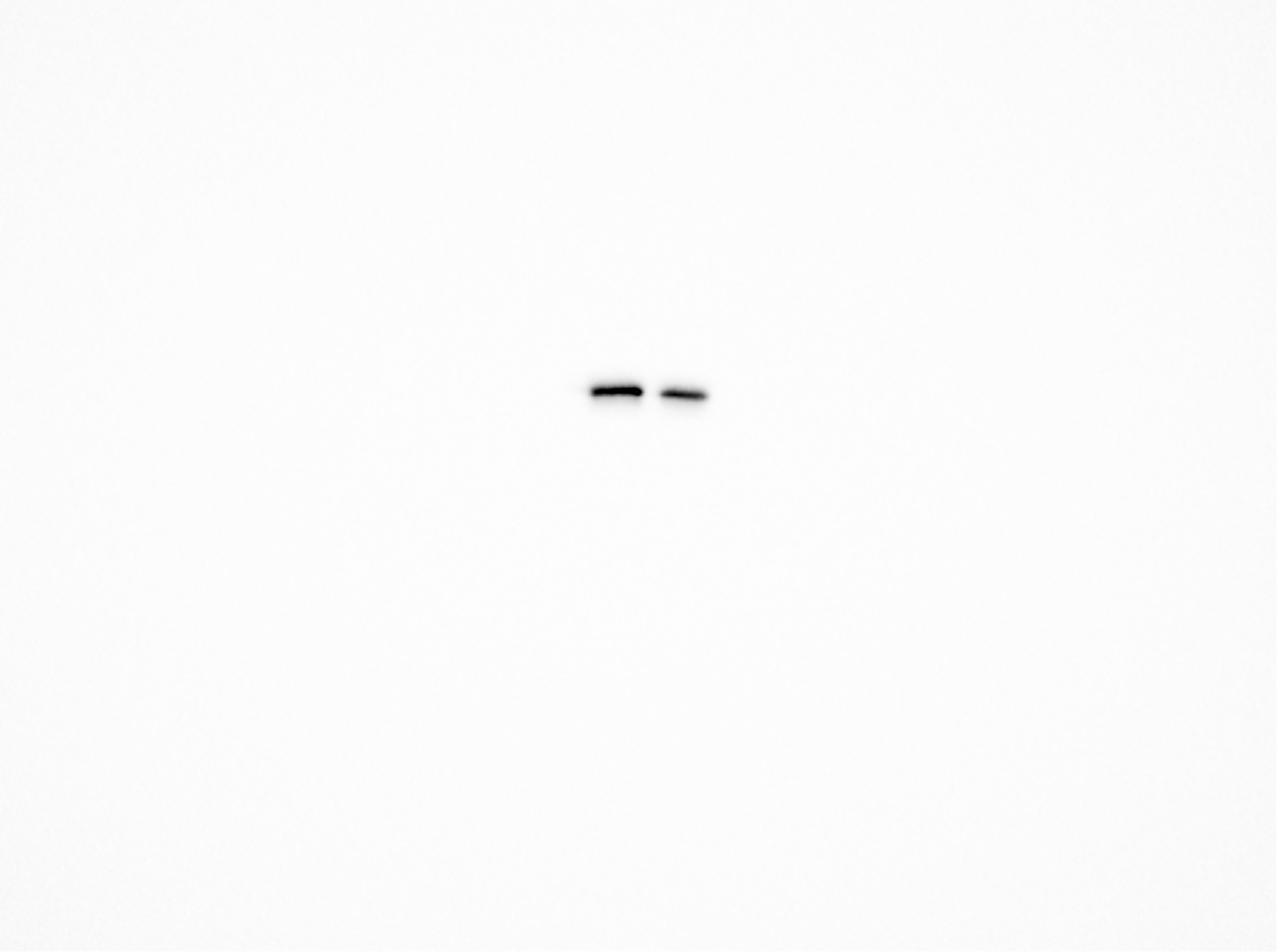


**Figure S4H 3-IL5RA**


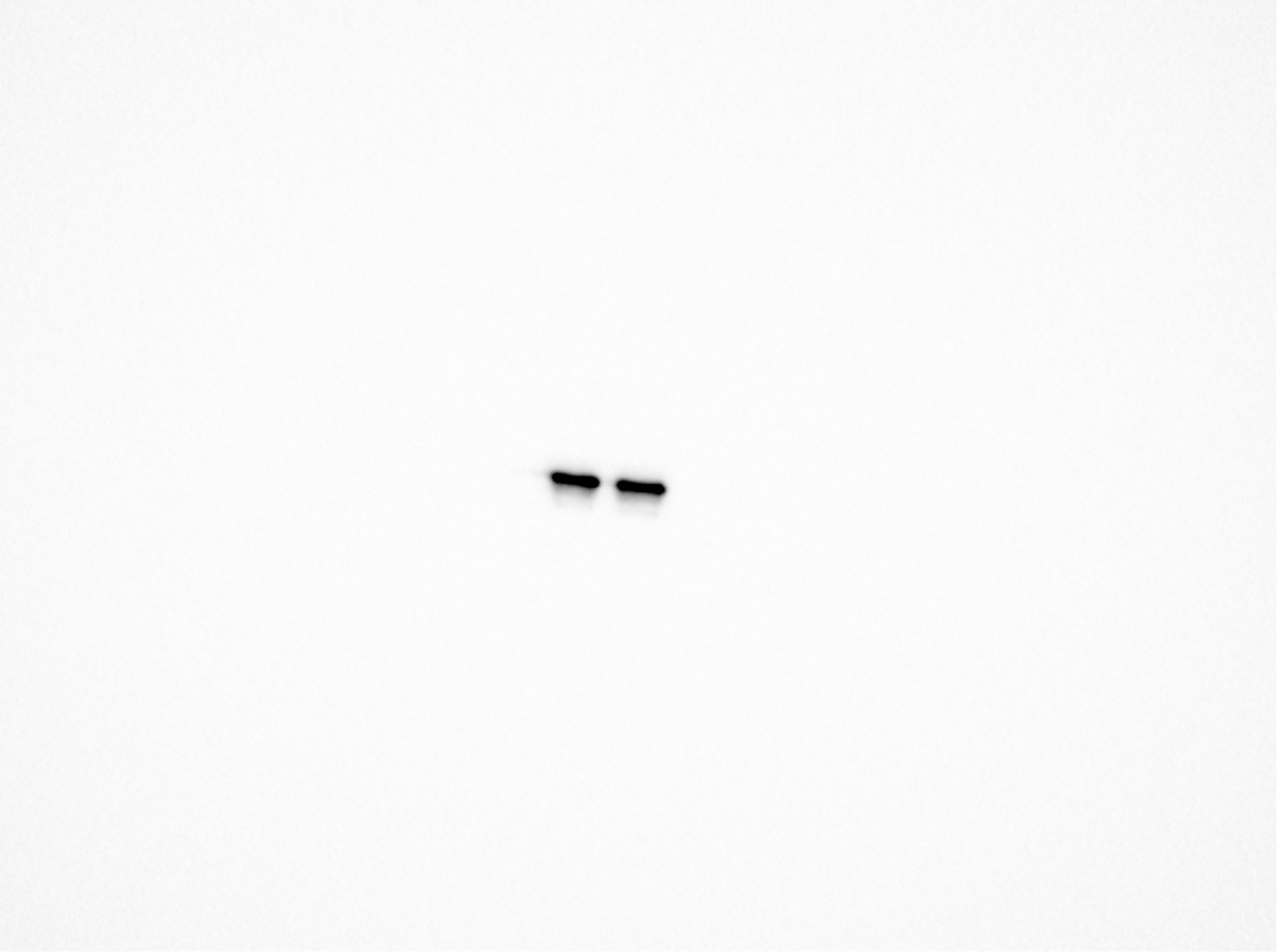


**Figure S4H 4-GAPDH**


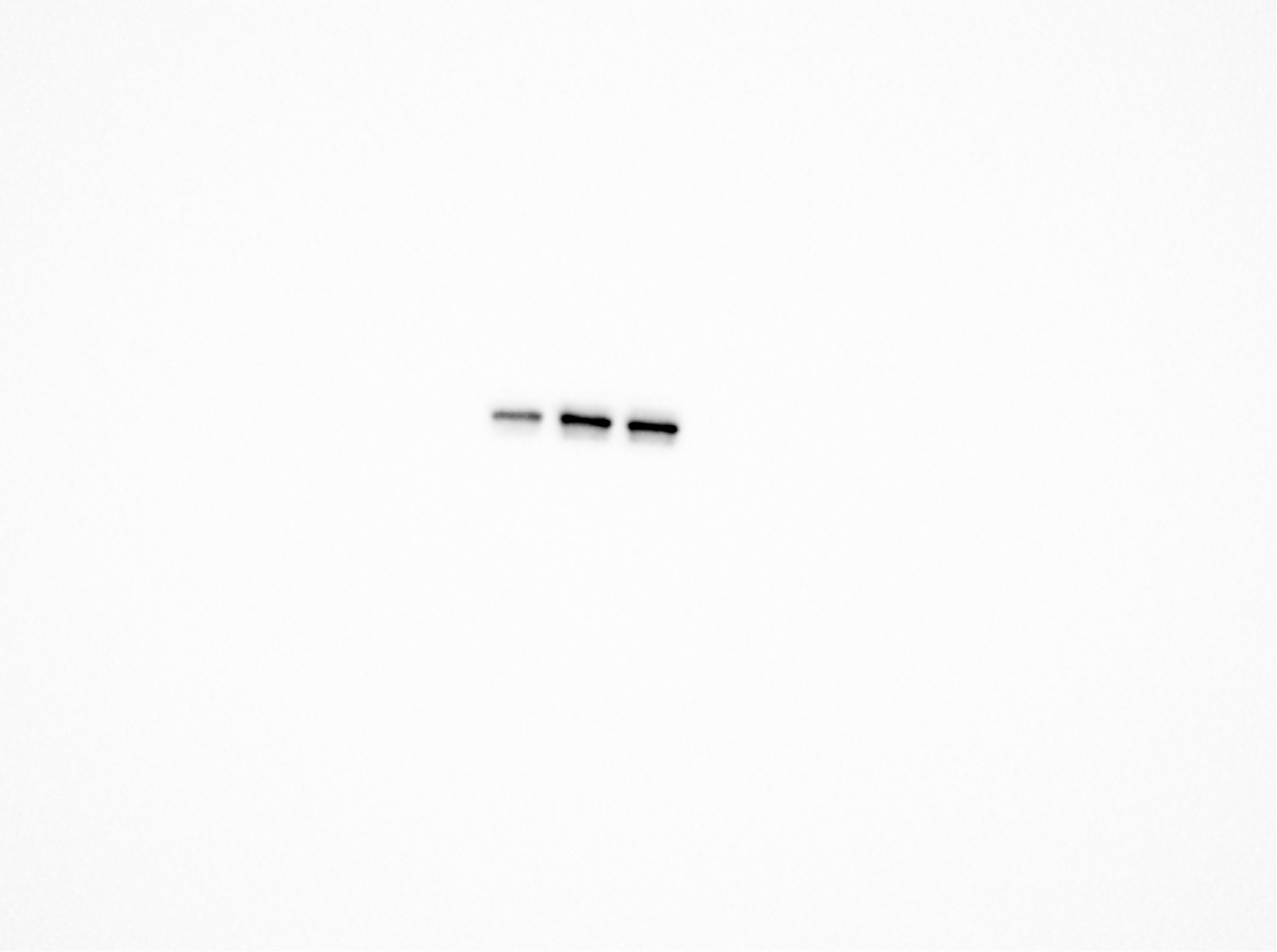


**Figure S4I 1-MDM2**


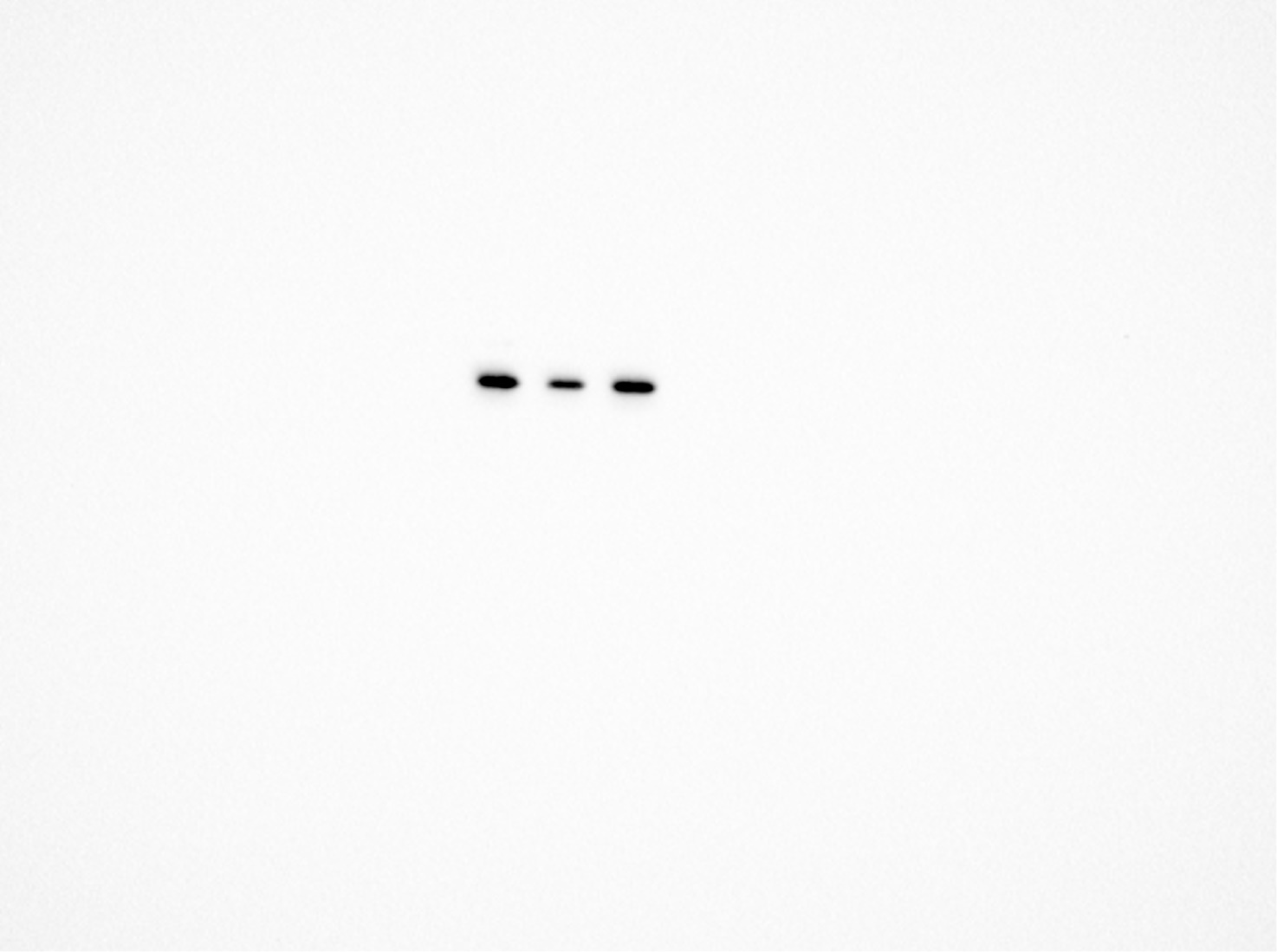


**Figure S4I 2-p53**


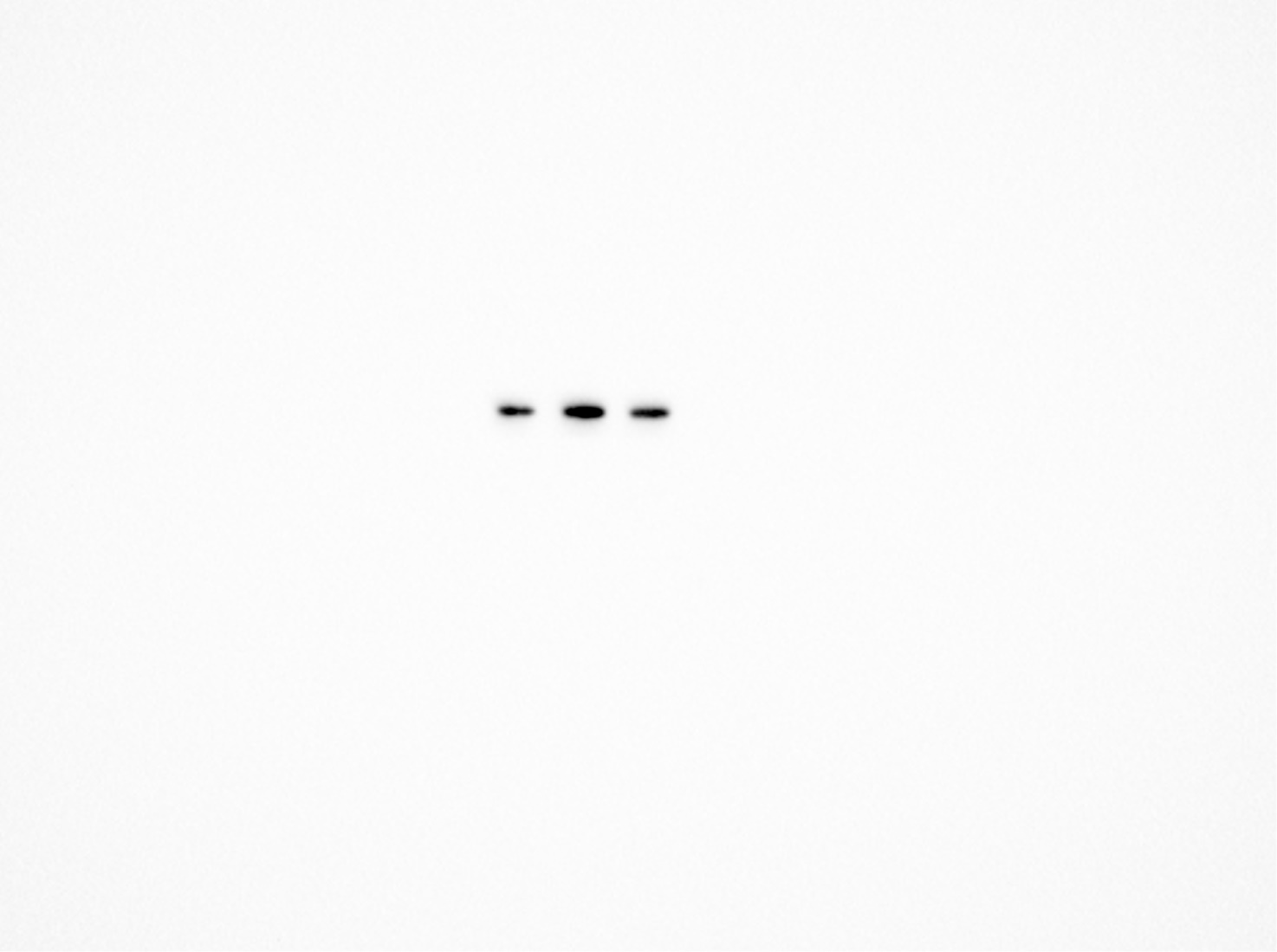


**Figure S4I 3-IL5RA**


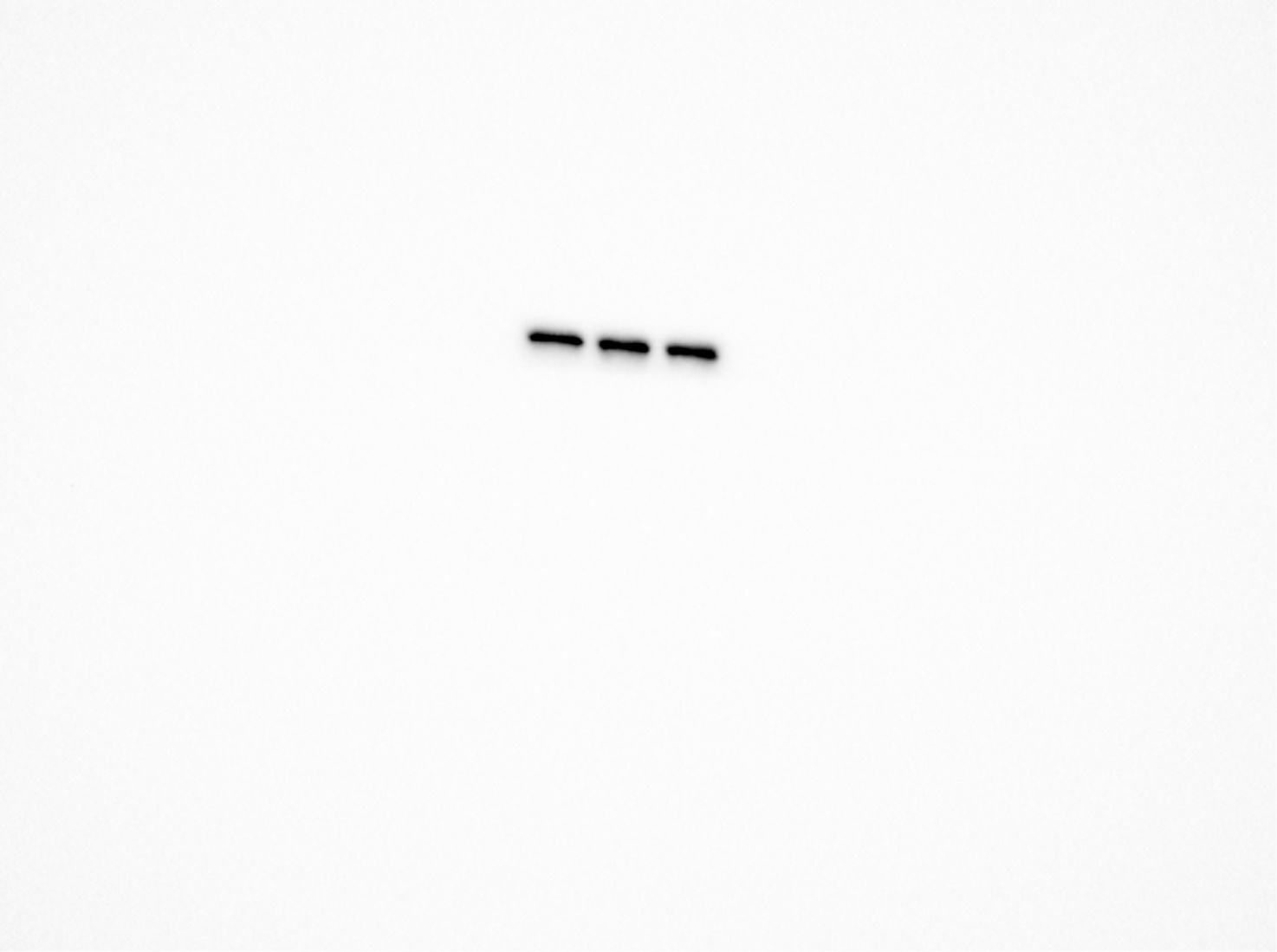


**Figure S4I 4-GAPDH**


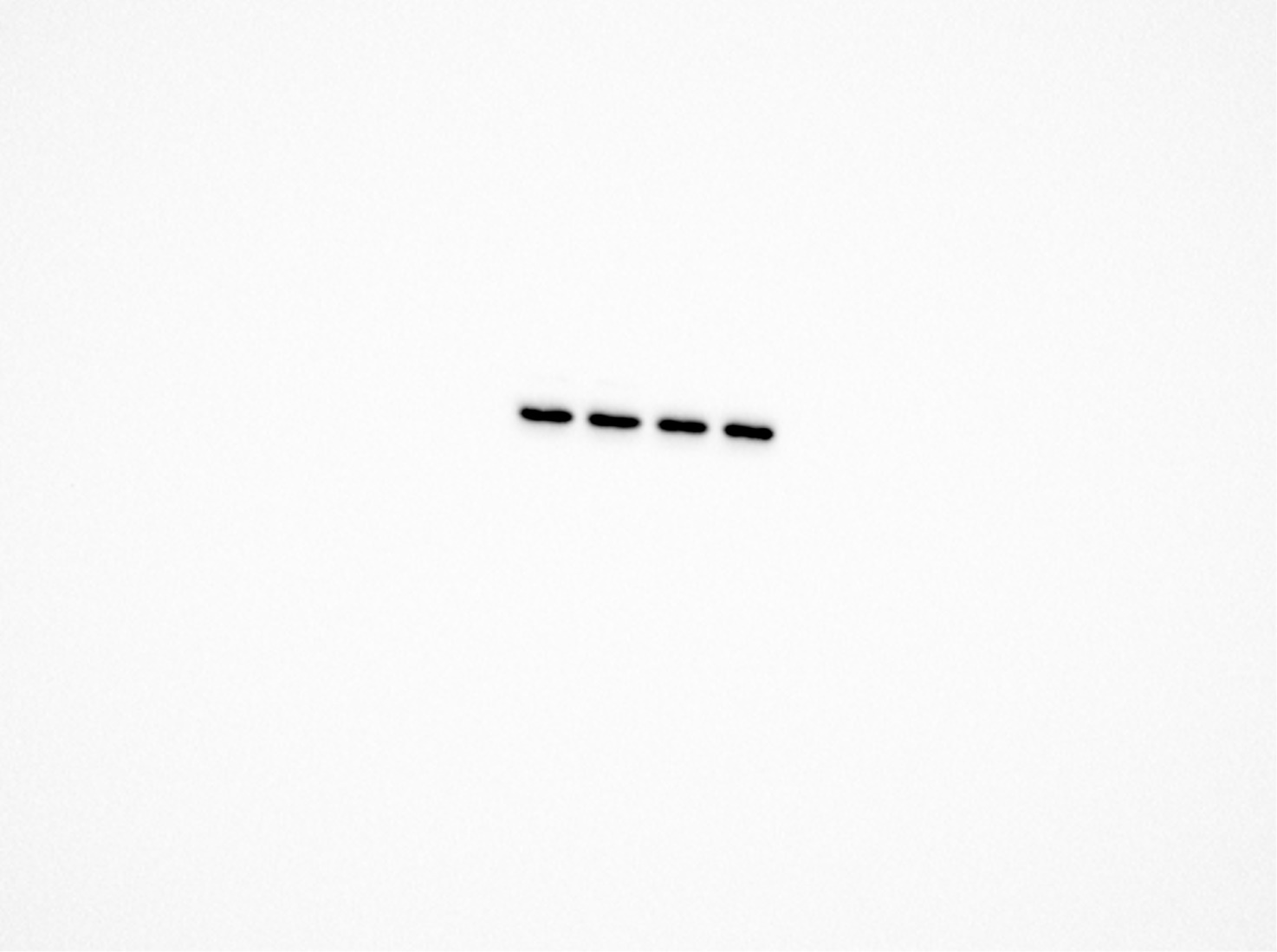


**Figure S4J 1-MDM2**


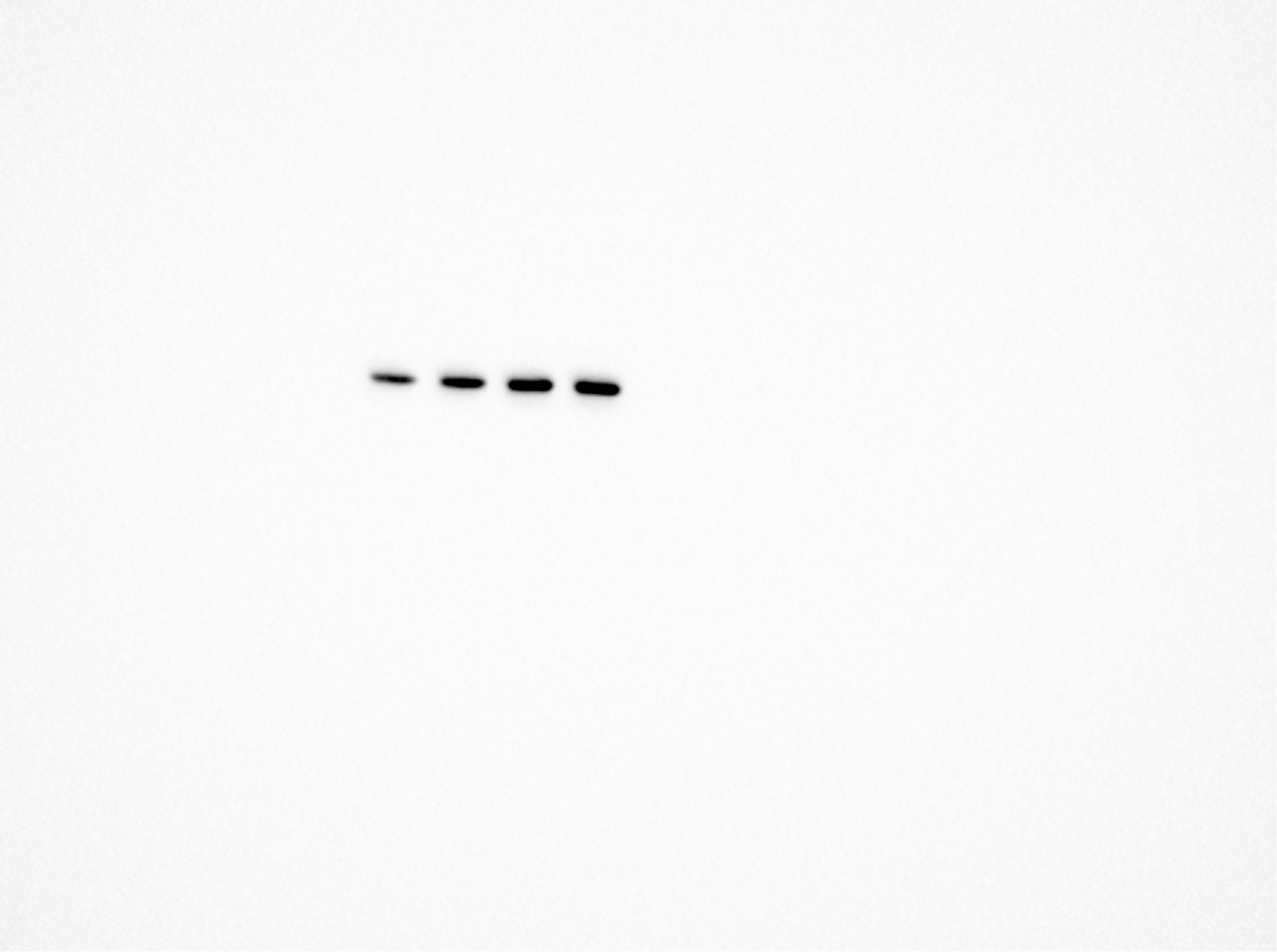


**Figure S4J 2-p53**


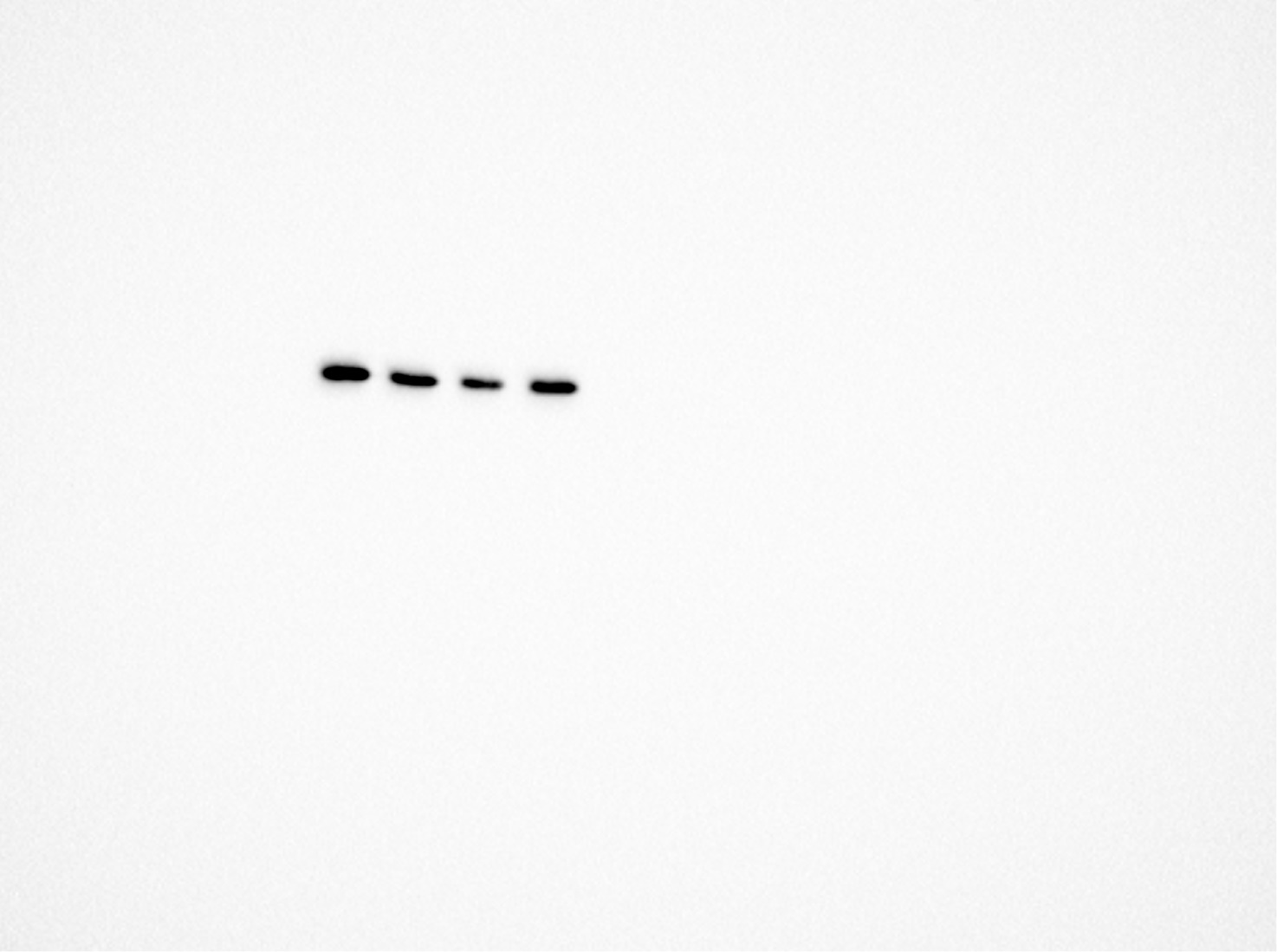


**Figure S4J 3-IL5RA**


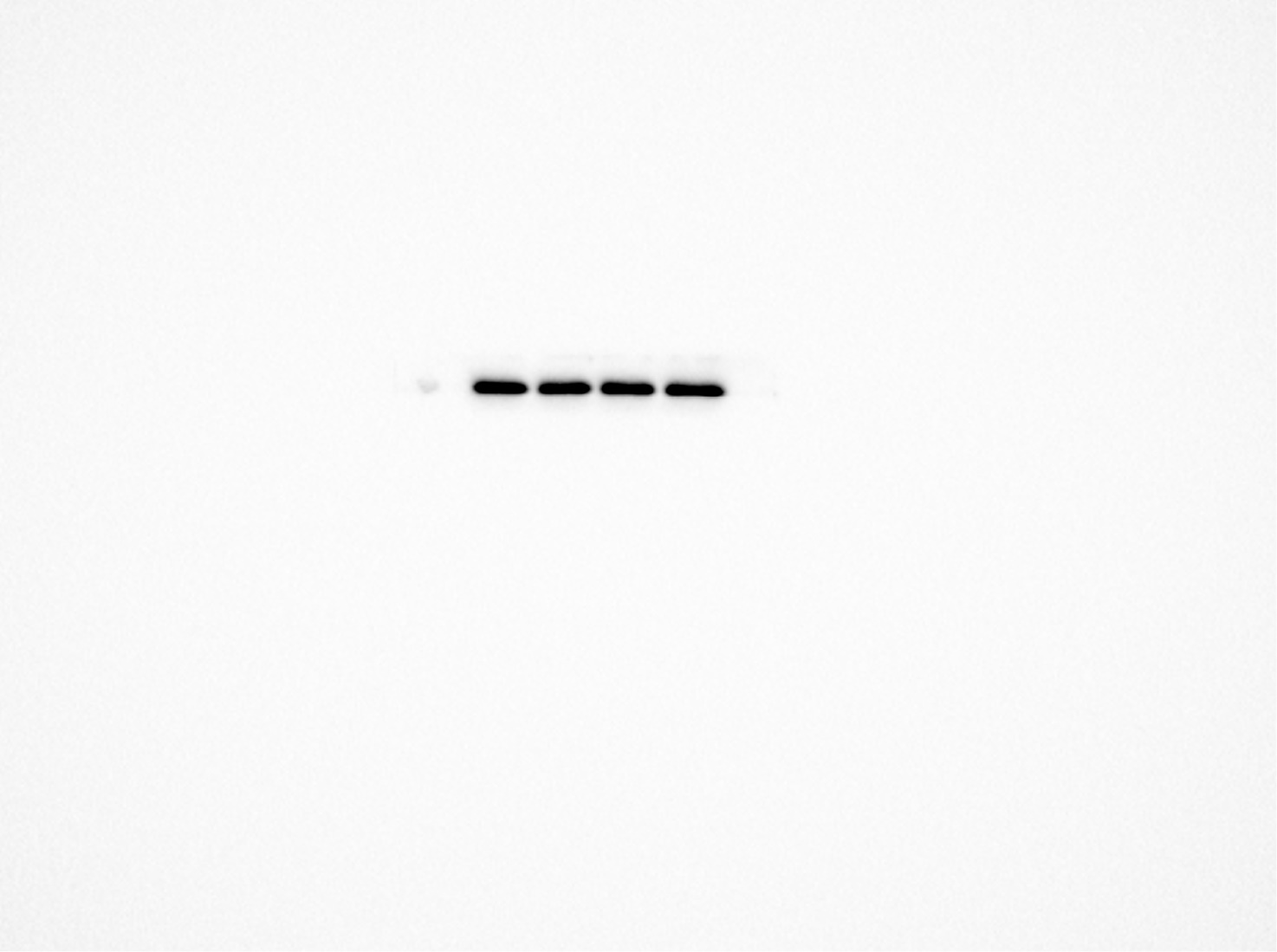


**Figure S4J 4-GAPDH**


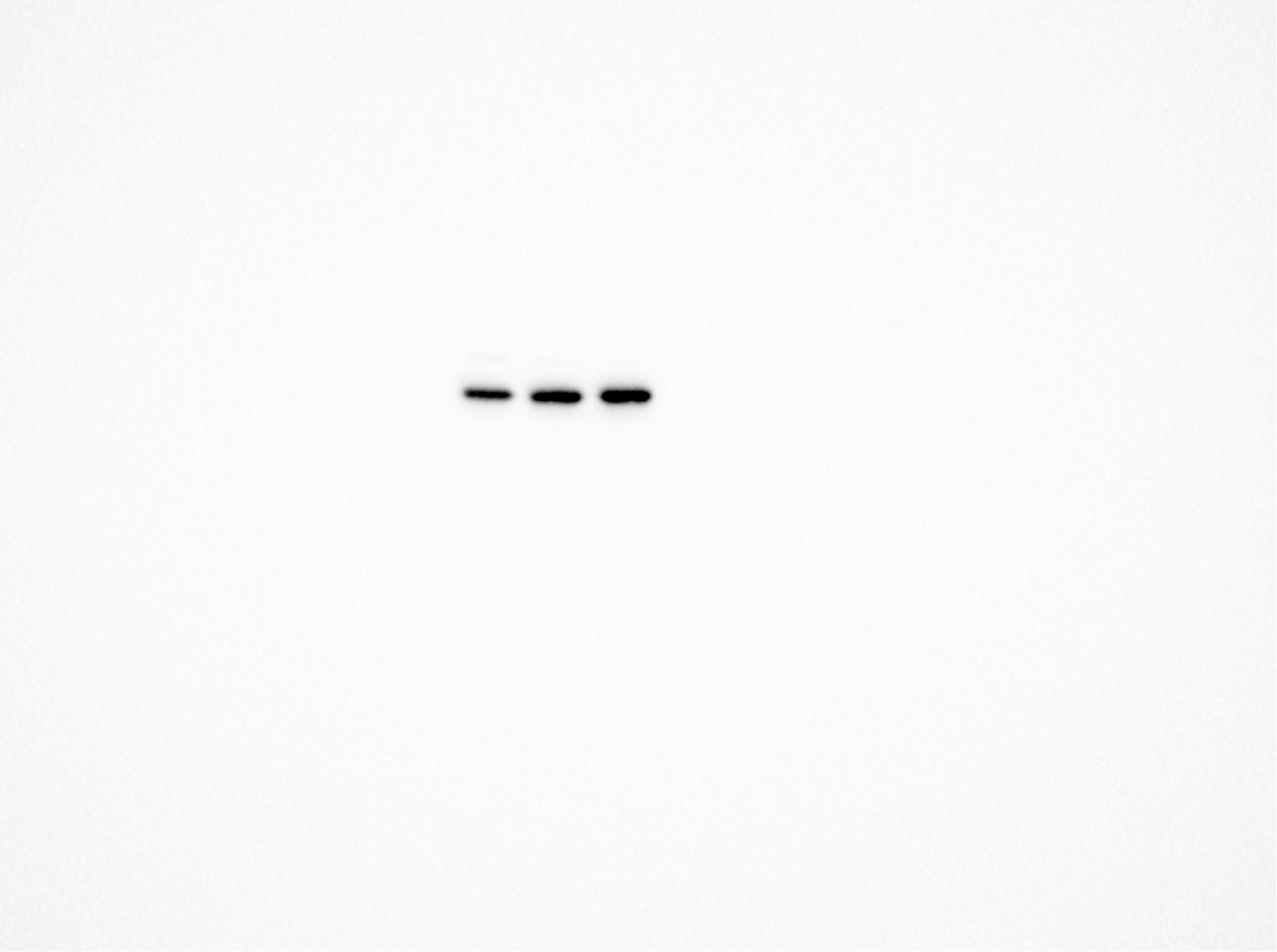


**Figure S4K 1-JMJD2C**


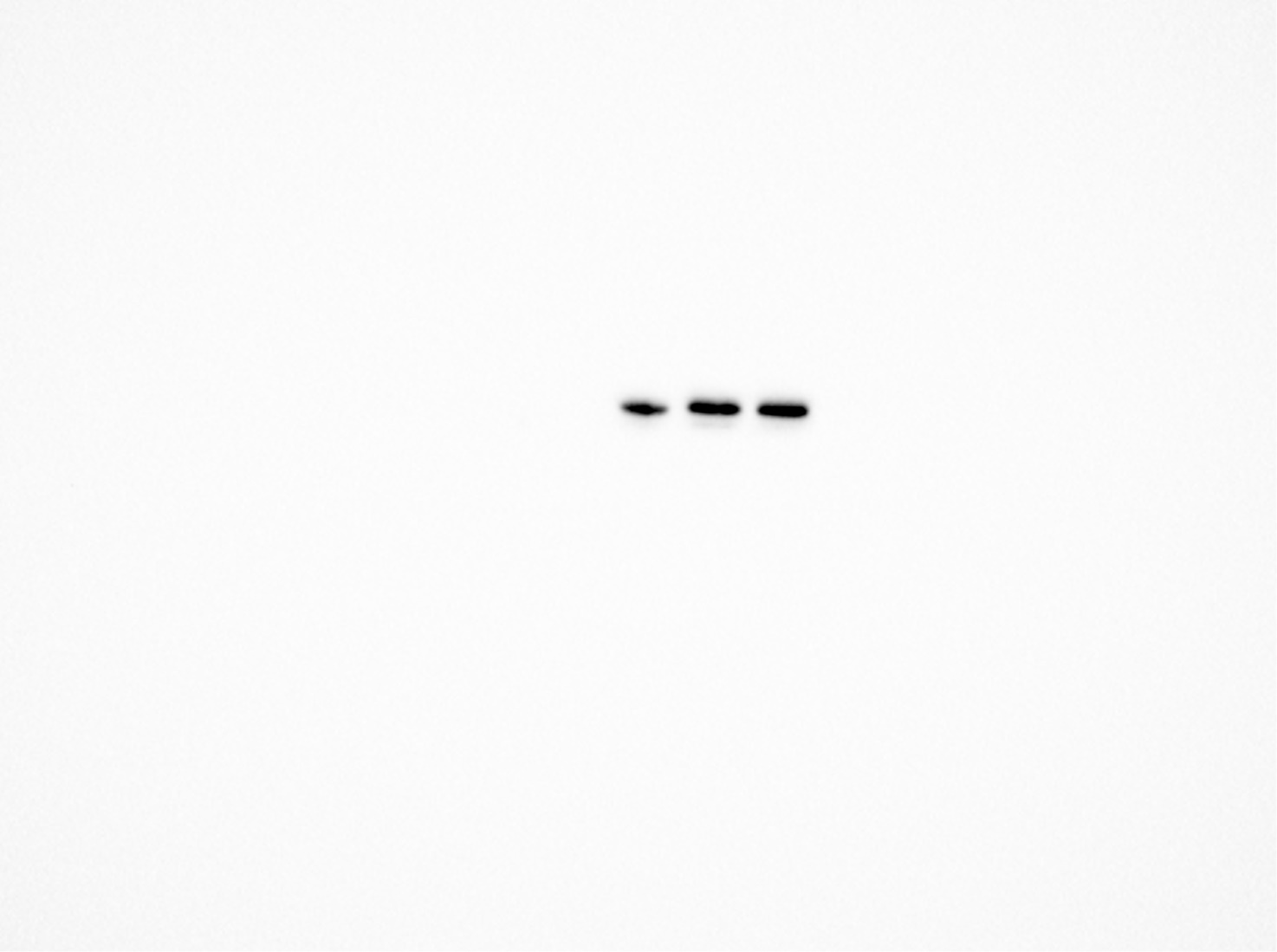


**Figure S4K 2-MDM2**


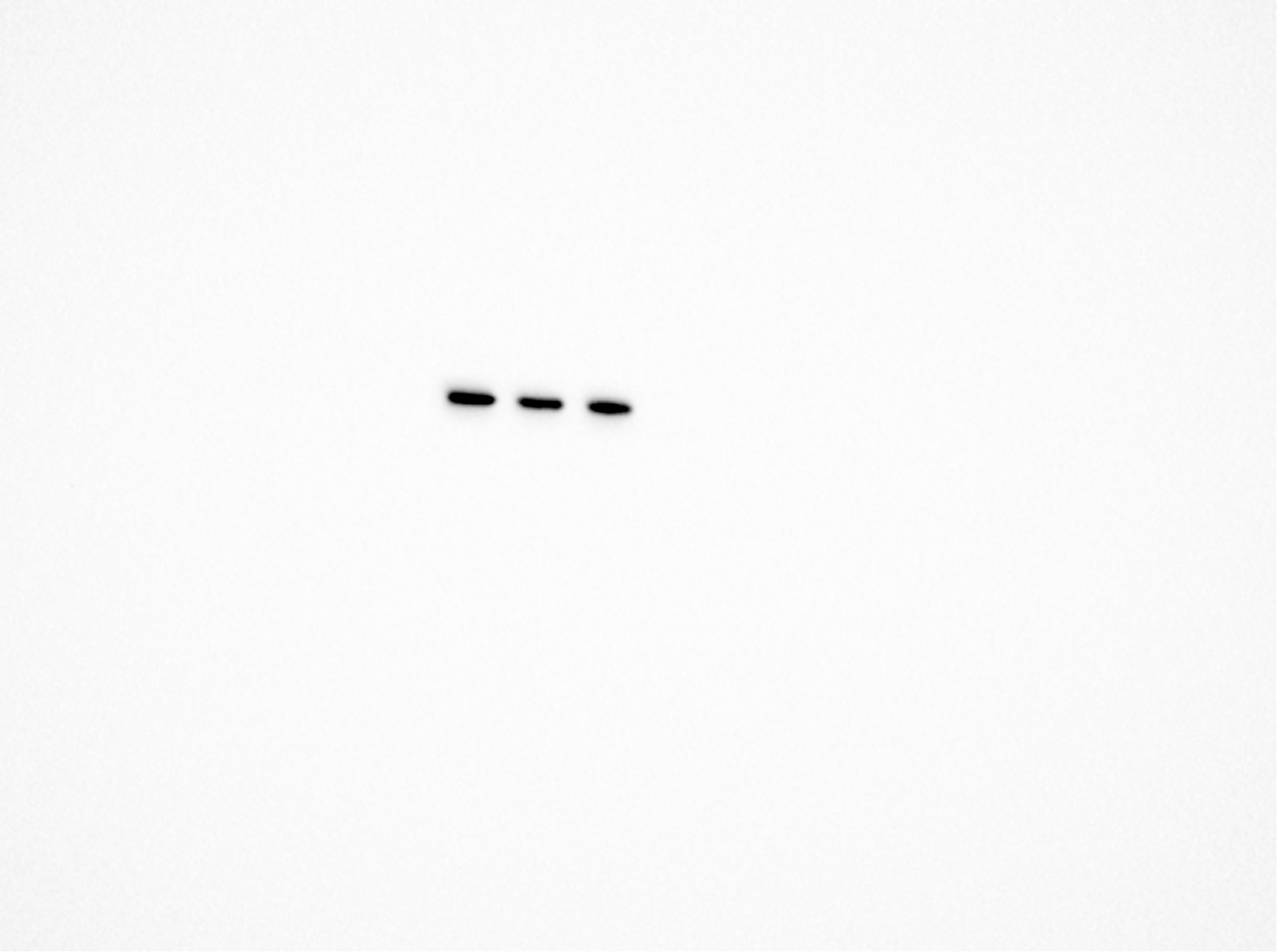


**Figure S4K 3-p53**


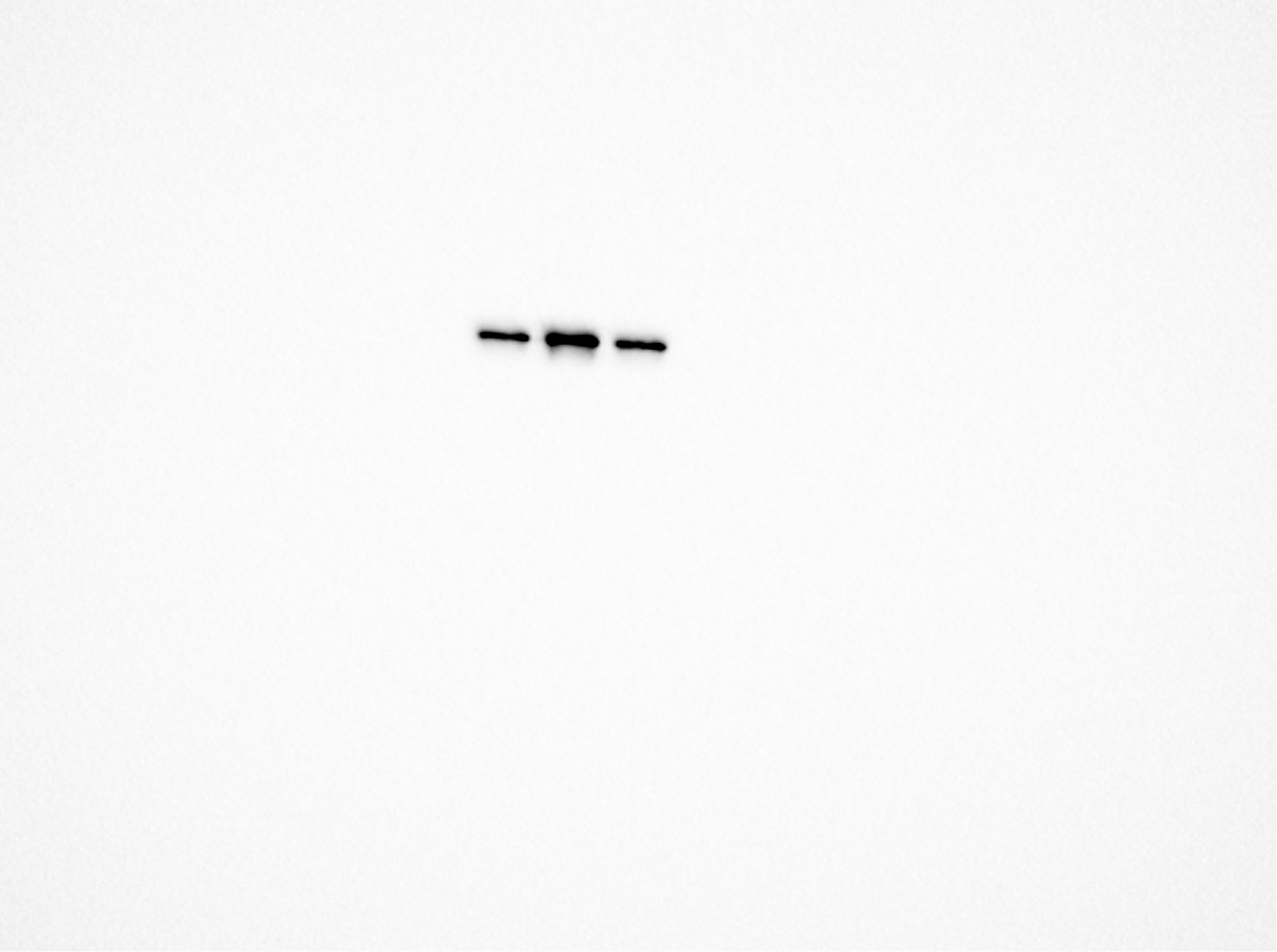


**Figure S4K 4-IL5RA**


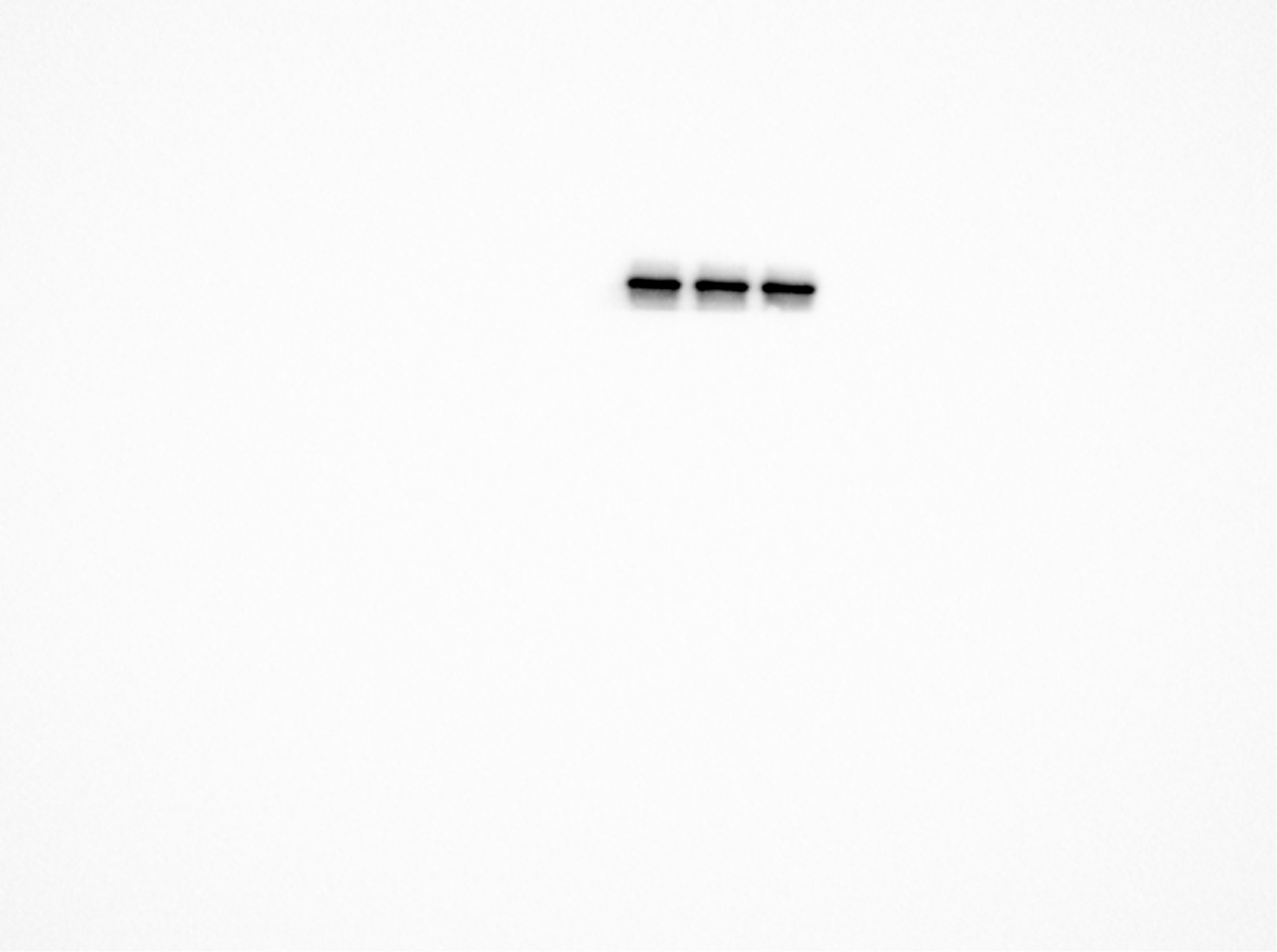


**Figure S4K 5-GAPDH**


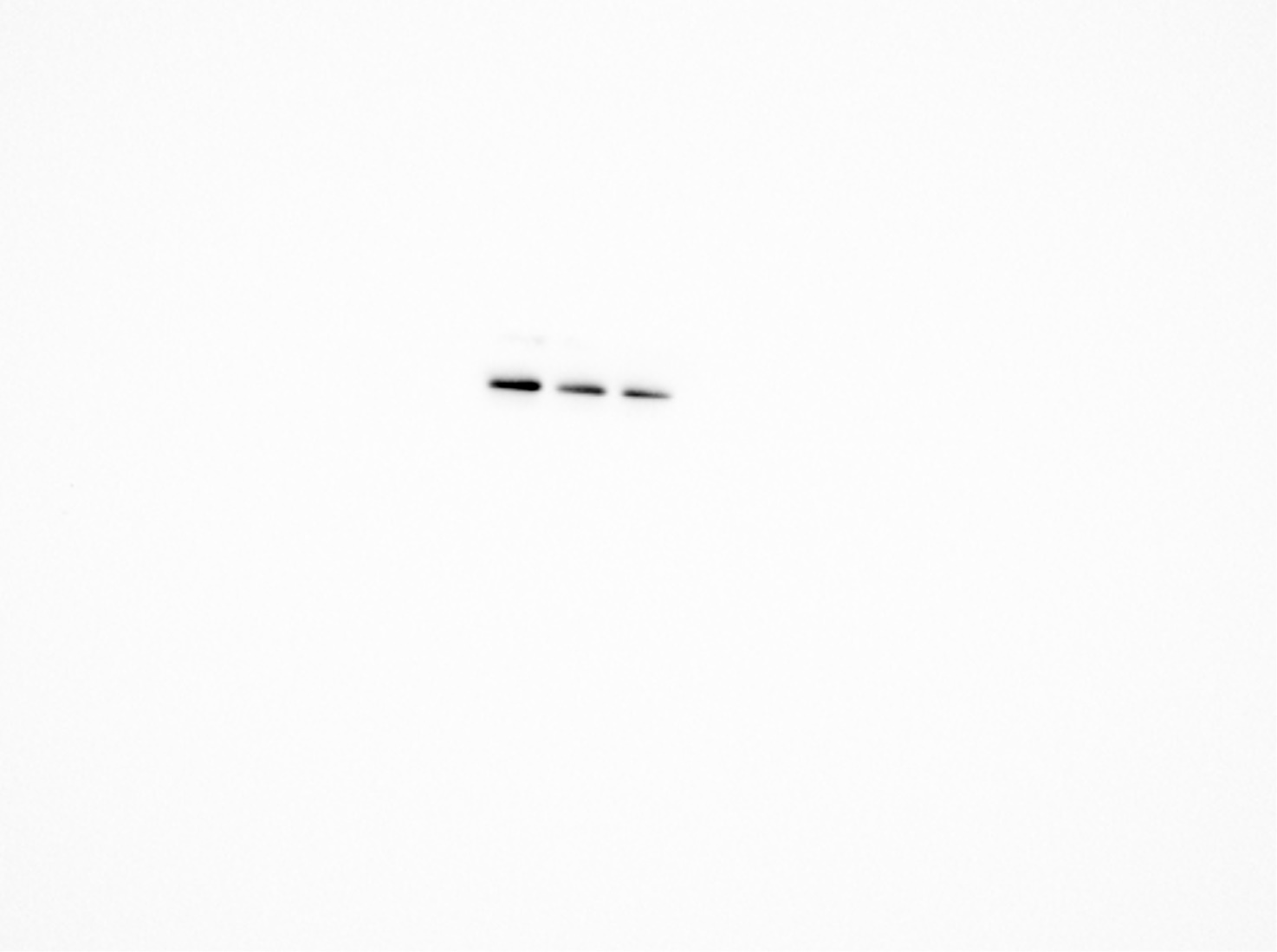


**Figure S4K 6-JMJD2C**


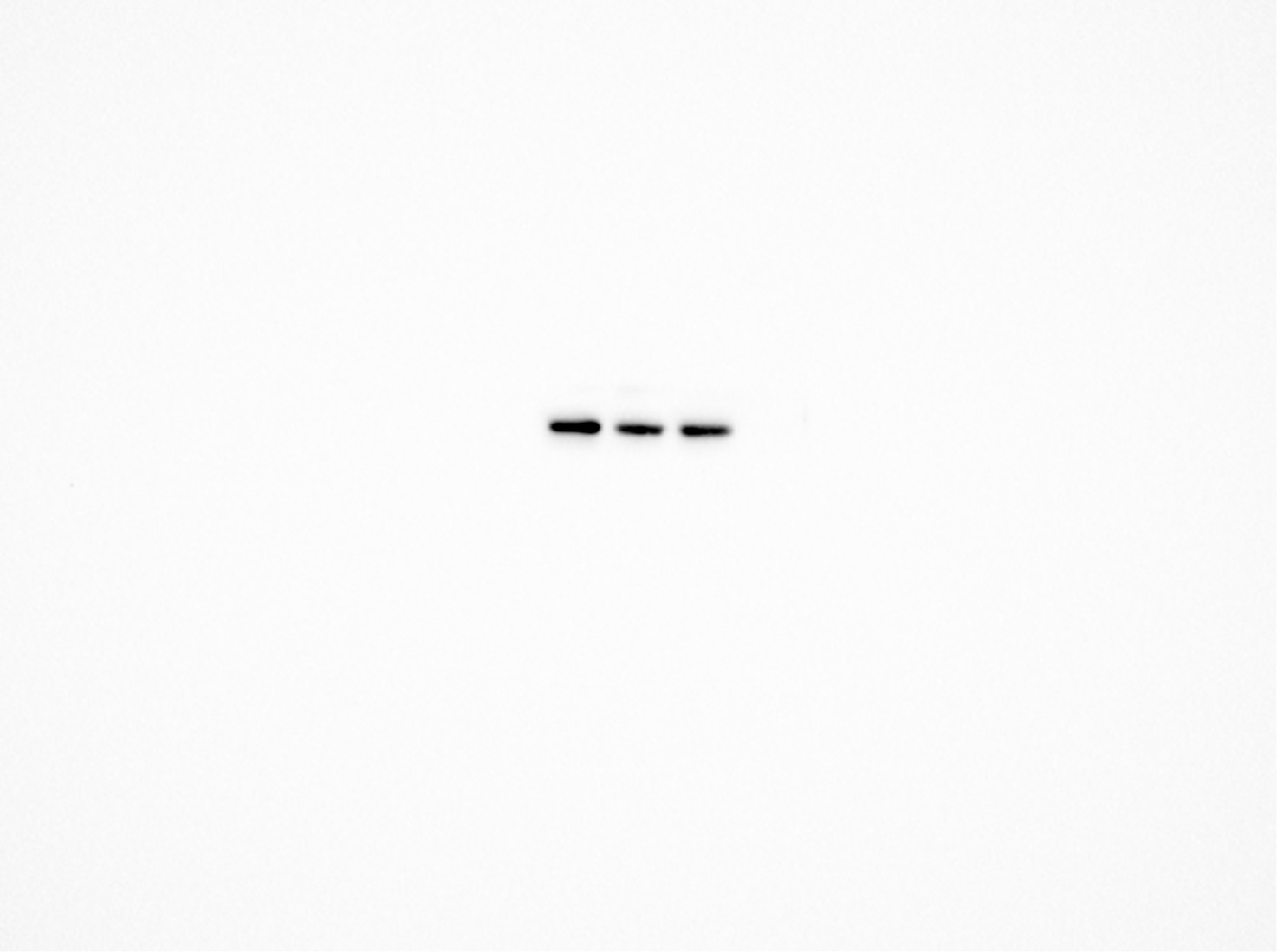


**Figure S4K 7-MDM2**


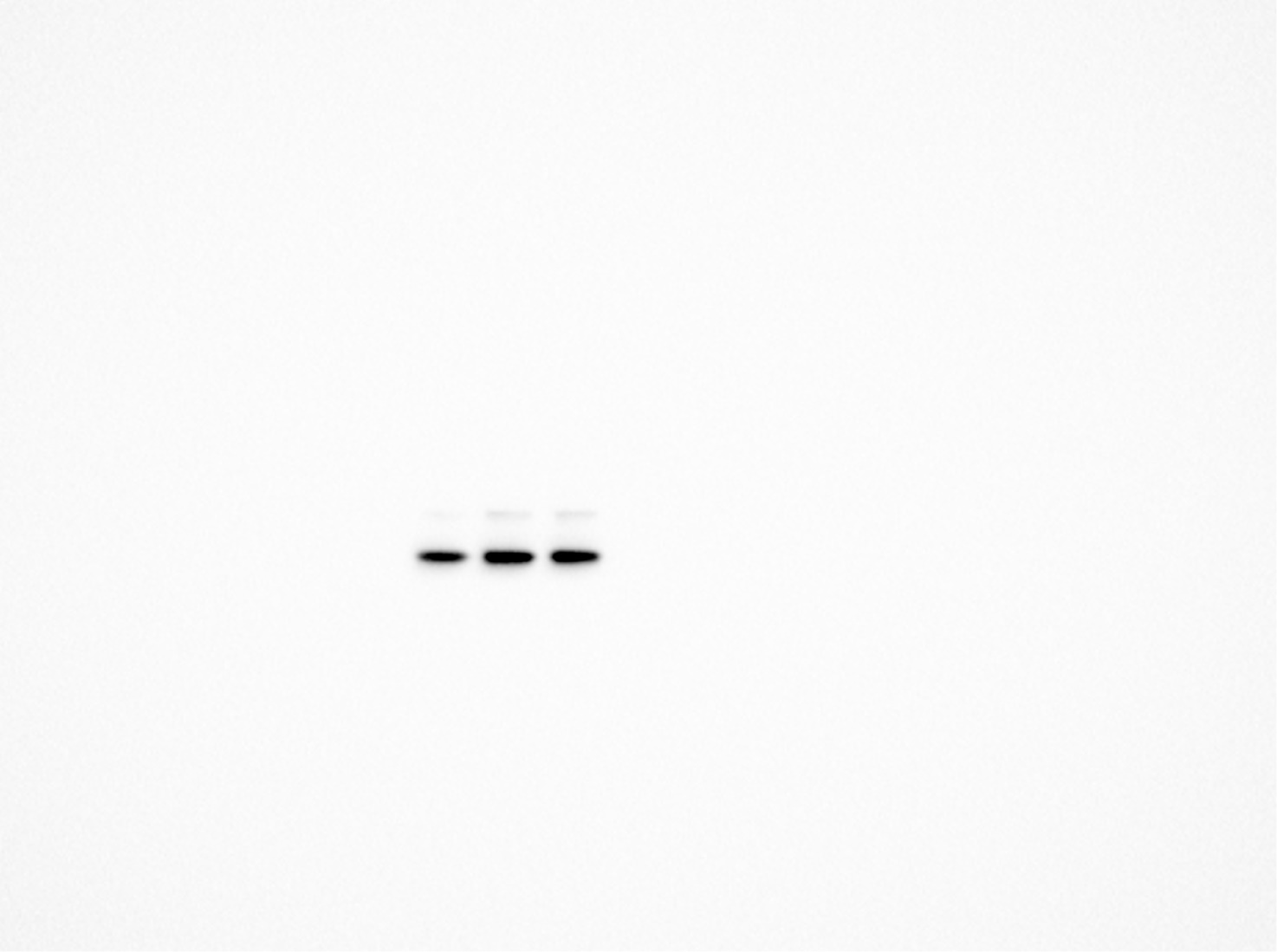


**Figure S4K 8-p53**


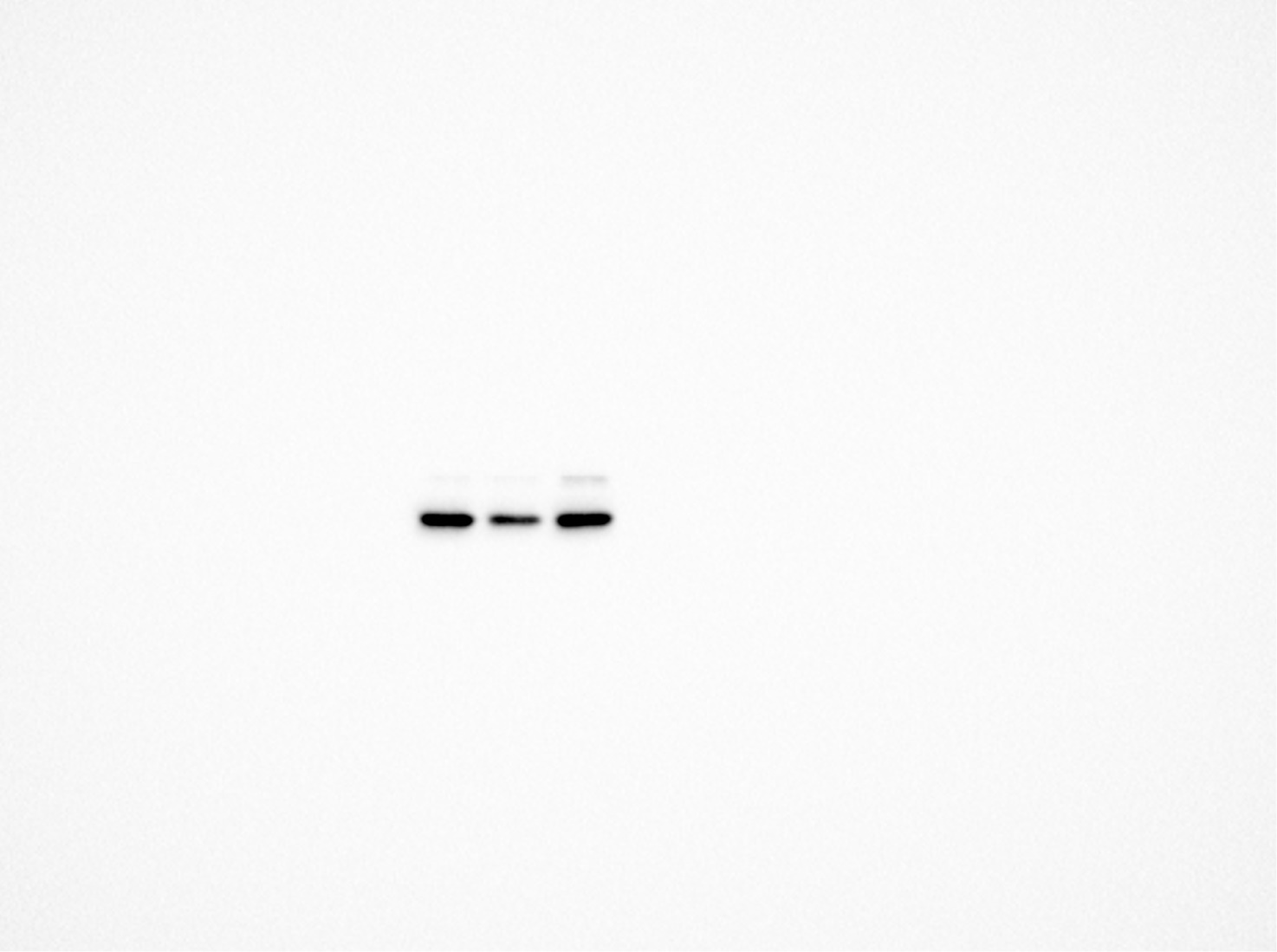


**Figure S4K 9-IL5RA**


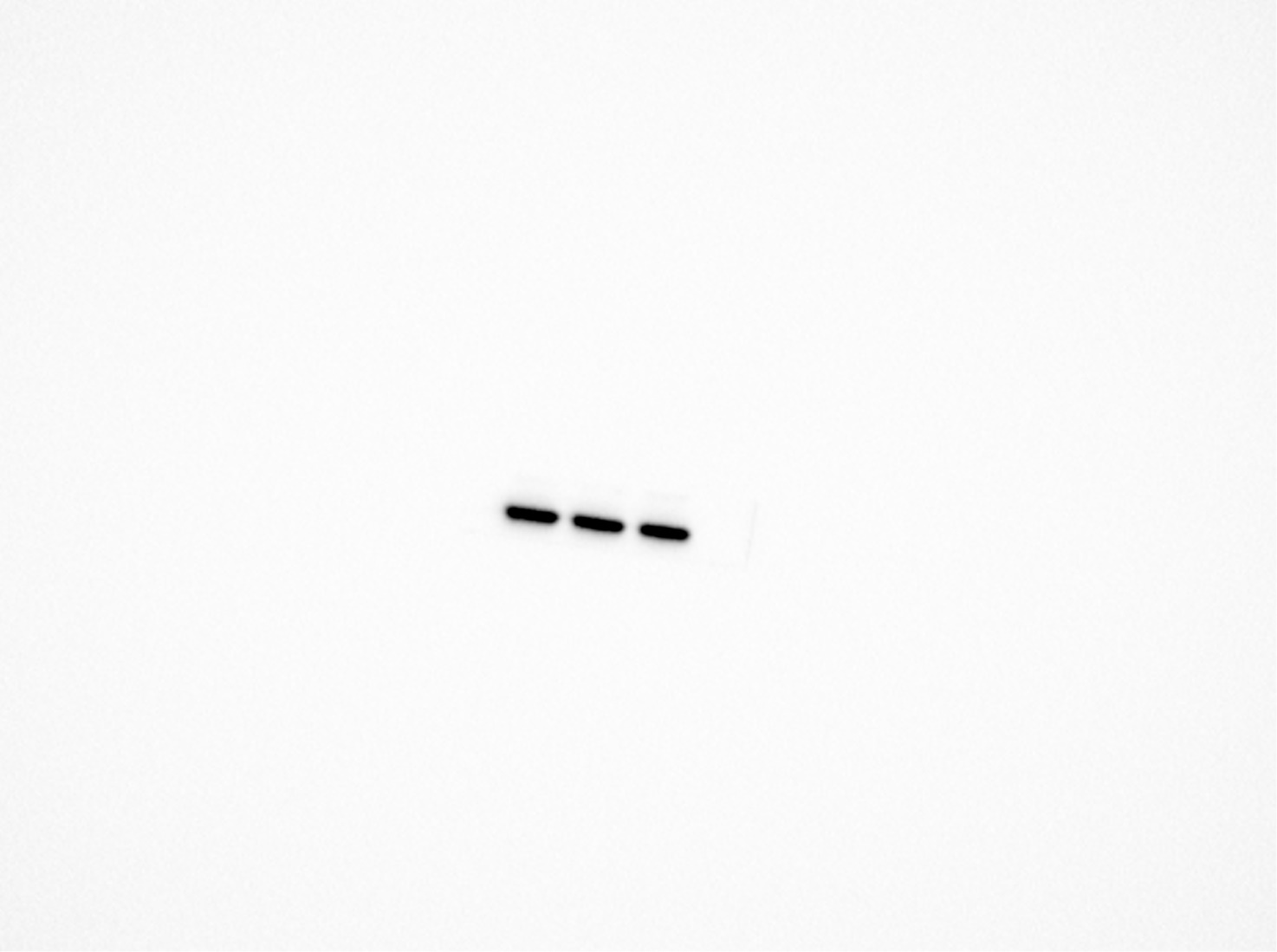


**Figure S4K 10-GAPDH**
