# Supplementary material for: JMJD2C mediates the MDM2/p53/IL5RA axis to promote CDDP resistance in uveal melanoma
Source: Cell Death Discov. 2022 Apr 25;8:227. doi: 10.1038/s41420-022-00949-y (PMC9039082; doi:10.1038/s41420-022-00949-y)
Supplement: Supplementary file 8 — author-contribution [file 41420_2022_949_MOESM8_ESM.pdf]

DECLARATION OF CONTRIBUTIONS TO ARTICLE

ADMC

Manuscript Number: CDDISCOVERY-21-1886

Journal Name: *Cell Death Discovery* (the 'Journal')

Proposed Title of the Contribution:

JMJD2C mediates the MDM2/p53/IL5RA axis to promote CDDP resistance in uveal melanoma (the 'Contribution)

Author(s):

Qi Zhu, Han Chen, Xiaoying Li, Xi Wang, Hongtao Yan (the 'Authors')

For all *CDDis*covery articles, each person named as an author in the published version must be able to show he or she has contributed substantially to the article.

Authorship credit should be based on 1) substantial contributions to conception and design, acquisition of data, or analysis and interpretation of data; 2) drafting the article or revising it critically for important intellectual content; and 3) final approval of the version to be published. Authors should meet conditions 1, 2 and 3.

Any person who cannot be shown to have made a substantial contribution to the article cannot be listed as an author in the final version. The name of any person who is deemed to have made a minor contribution can, however, appear in the Acknowledgments section of the article.

Please complete the table below to indicate the contributions of all named authors to the manuscript.

Author Full Name:	Specification of Contribution to the Manuscript:
Qi Zhu	performed experiments and analyzed data; edited and revised manuscript
Han Chen	prepared figures
Xiaoying Li	drafted manuscript
Xi Wang	conceived and designed research; drafted manuscript
Hongtao Yan	edited and revised manuscript

Please complete the table below to indicate the contributions of all named authors to the figures.

Figure 1:

Qi Zhu, Han Chen, Xiaoying Li, Xi Wang, Hongtao Yan

Figure 2:

Qi Zhu, Han Chen, Xiaoying Li, Xi Wang, Hongtao Yan

Figure 3:

Qi Zhu, Han Chen, Xiaoying Li, Xi Wang, Hongtao Yan

Figure 4:

Qi Zhu, Han Chen, Xiaoying Li, Xi Wang, Hongtao Yan

Figure 5:

Qi Zhu, Han Chen, Xiaoying Li, Xi Wang, Hongtao Yan

Figure 6:

Qi Zhu, Han Chen, Xiaoying Li, Xi Wang, Hongtao Yan

Signed for and on behalf of the Author(s):	Print Name:	Date:
Hongtao Yan	Hongtao Yan	2021/12/20
